# First-line treatment with chemotherapy, surufatinib (an angio-immuno kinase inhibitor), and camrelizumab (an anti-PD-1 antibody) for locally advanced or metastatic pancreatic ductal adenocarcinoma: a phase Ib/II randomized study

**DOI:** 10.1038/s41392-025-02441-2

**Published:** 2025-10-13

**Authors:** Ru Jia, Hai-Yan Si, Meng-Jiao Fan, Nan Zhang, Guo-Chao Deng, Fang-Fang Liu, Lu Han, Miao-Miao Gou, Zhao-Li Tan, Xia Zhang, Yan-Rong Wang, Yue Shi, Yao-Yue Zhang, Yu-Shan Jia, Yu-Qi Wang, Quan-Li Han, Zhi-Kuan Wang, Guang-Hai Dai

**Affiliations:** 1 Senior Department of Oncology, Chinese PLA General Hospital, Beijing, China; 2 Geneseeq Research Institute, Nanjing Geneseeq Technology Inc, Nanjing, China

**Keywords:** Gastrointestinal cancer, Clinical trials

## Abstract

Pancreatic ductal adenocarcinoma (PDAC) has a poor prognosis and limited first-line treatments. This phase Ib/II randomized trial (NCT05218889) investigated the efficacy and safety of surufatinib plus camrelizumab and nab-paclitaxel/S-1 (NASCA) versus nab-paclitaxel and gemcitabine in patients with locally advanced or metastatic PDAC. The primary endpoints were dose-limiting toxicities and the recommended phase II dose (RP2D) of surufatinib in phase Ib, and the objective response rate (ORR) in phase II. Phase Ib used a 3 + 3 dose-escalation design to determine the RP2D of surufatinib in six patients, which was established at 200 mg. In phase II, patients were randomized 1:1 to receive the NASCA (45 patients) or nab-paclitaxel and gemcitabine (45 patients). NASCA group showed an ORR of 51.1% (23/45) versus 24.4% (11/45) in the nab-paclitaxel and gemcitabine group (odds ratio 3.2, 95% CI 1.3–8.2, *p* = 0.01). The median progression-free survival (PFS) was 7.9 vs. 5.3 months (HR 0.63, 95% CI 0.40–0.99, *p* = 0.045). The median overall survival was 13.0 vs. 11.0 months (HR 0.77, 95% CI 0.47–1.28, *p* = 0.318). The most common Grade ≥3 treatment-related adverse event was decreased neutrophil count (33.3% vs. 35.6%). In the NASCA group, enrichment of CD8^+^ and CD8^+^PD-1^+^ cells, a high baseline M1/M2 macrophage ratio, and a reduction in CA19-9 levels at weeks 6 and 12 were associated with improved PFS compared to patients without these features. The NASCA regimen showed promising efficacy with tolerable safety relative to nab-paclitaxel and gemcitabine for locally advanced or metastatic PDAC.

## Introduction

The prognosis for pancreatic ductal adenocarcinoma (PDAC) remains poor, making it a particularly difficult disease to manage. Data from the Global Cancer Statistics 2022 indicate that there were 511,000 newly diagnosed cases and 467,000 deaths, placing this cancer 12th in terms of incidence and 6th for cancer-related deaths globally.^1,2^ As most individuals receive their diagnosis at a late stage, just approximately 12% are anticipated to survive for more than 5 years.^3^ First-line management of metastatic PDAC predominantly involves systemic chemotherapy, with regimens like nab-paclitaxel combined with gemcitabine, FOLFIRINOX (irinotecan, leucovorin, fluorouracil, and oxaliplatin), and NALIRIFOX (folinic acid, liposomal irinotecan, fluorouracil, and oxaliplatin) reporting median overall survival (OS) durations of 8.5, 11.1, and 11.1 months, accordingly.^4–6^ Furthermore, immunotherapy efficacy remains unsatisfactory for most patients, except for a small subgroup (~1%) with MSI-high/dMMR.^7^ For example, treatments involving durvalumab alone or in combination with tremelimumab, as well as nivolumab combined with chemotherapy, have shown only limited responses.^8,9^

Immunotherapy approaches showed limited efficacy in PDAC, potentially owing to the particular properties of the tumor and tumor microenvironment (TME).^10^ The hallmarks of PDAC TME include desmoplastic fibrotic stroma and hypovascularity resulting in increased interstitial pressure, poor tumor perfusion, intratumoral hypoxia, immune suppression, and inadequate delivery of systemic drugs.^10,11^ Addressing the poor vascularization characterized by immature and inefficient blood vessels may offer a novel avenue for PDAC therapy. Vascular normalization with increased pericyte coverage of vessels and improved tumor perfusion in an orthotopic PDAC mouse model resulted in decreased tumor hypoxia and enhanced chemotherapy delivery.^12^ Moreover, normalizing blood vessels with anti-angiogenic drugs with low doses can reduce immunosuppression by restricting the functions of regulatory T (Treg) cells and myeloid-derived suppressor cells (MDSCs). This approach improves T cell activation and infiltration into tumors.^13,14^ Therefore, we hypothesize that using anti-angiogenic agents to promote vascular normalization may enhance chemotherapy delivery, alleviate immune suppression, and improve tumor sensitivity to immunotherapy. Promising findings were reported in a phase II study assessing penpulimab (an anti-PD-1 antibody) plus anlotinib (an inhibitor of angiogenesis), nab-paclitaxel, and gemcitabine, as first-line treatment for metastatic pancreatic cancer. The regimen achieved a 50.0% objective response rate (ORR), 95.5% disease control rate (DCR), and median progression-free survival (PFS) and OS of 8.8 months and 13.7 months, respectively.^15^

S-1, an oral fluoropyrimidine, is better tolerated in East Asian patients due to ethnic differences in drug metabolism and is widely used for gastrointestinal cancers in this population.^16–20^ Its efficacy in PDAC among East Asians is well established. According to the JASPAC01 trial, S-1 demonstrated greater efficacy than gemcitabine in the adjuvant context, and results from the GEST trial indicated that S-1 offers comparable effectiveness to gemcitabine as initial treatment for advanced PDAC.^21,22^ These conclusions were further backed by a network meta-analysis from Japan, which demonstrated that first-line treatment with S-1 yields survival outcomes comparable to those with gemcitabine in metastatic PDAC.^23^ Both trials also reported lower rates of adverse events (AEs), especially hematologic toxicity, with S-1 compared to gemcitabine.^21,22^ A preclinical study in pancreatic cancer suggest that S-1 plus nab-paclitaxel may exert synergistic antitumor effects, potentially through stromal depletion and improved vascularization.^24^ This doublet regimen offers similar efficacy to nab-paclitaxel plus gemcitabine, with better tolerability, and has recently been explored in China, especially at our center.^25–29^ A retrospective study showed that adding PD-1 blockade to this combination did not increase Grade ≥3 AEs, indicating a manageable safety profile.^30^

Building on this rationale, we developed an exploratory multi-modal regimen combining chemotherapy, anti-angiogenic therapy, and immunotherapy to improve antitumor efficacy while maintaining acceptable safety. Given its favorable tolerability profile, S-1 plus nab-paclitaxel was selected as the chemotherapy backbone. To further influence the tumor microenvironment, we introduced surufatinib—a novel small-molecule agent that acts on tumor angiogenesis by inhibiting VEGFRs and FGFR1, and on immune escape mechanisms through CSF1R blockade.^31^ Two randomized phase III trials found that surufatinib provided a significant improvement in PFS for patients with advanced pancreatic and extra-pancreatic neuroendocrine tumors compared relative to placebo.^32,33^ Initial results from a phase I trial evaluating surufatinib in combination with PD-1 inhibition in advanced solid tumors showed anti-tumor activity and good tolerability.^34^ Camrelizumab, a PD-1 blocking antibody, showed clinical benefit at a 200 mg dose when administered alongside chemotherapy and radiotherapy in patients with locally advanced pancreatic adenocarcinoma.^35^ Drawing upon this evidence, we initiated a phase Ib/II randomized study investigating the combination of surufatinib, camrelizumab, nab-paclitaxel, and S-1 as a first-line treatment for patients with locally advanced or metastatic PDAC.

## Results

### Phase Ib study: dose determination

The phase Ib study aimed to identify the recommended phase II dose (RP2D) of surufatinib in combination with camrelizumab, nab-paclitaxel, and S-1. Surufatinib at 200 mg was initially administered to three patients alongside camrelizumab, nab-paclitaxel, and S-1. After one individual developed a dose-limiting toxicity (DLT) with Grade 3 diarrhea, three further patients were treated using the same dose regimen. The following three patients did not develop any DLTs. Among the six patients, AEs observed during the study also included Grade 2 dcreased neutrophil count(n = 3), Grade 3 dcreased neutrophil count(n = 1), Grade 4 dcreased neutrophil count (n = 1; noted as a single-day event and thus not meeting DLT criteria), Grade 3 immune-related hepatitis (n = 2; occurring at Weeks 8 and 18, respectively, both beyond Cycle 1), and Grade 2 nausea (n = 1). The ORR was 66.7% (4/6), with a DCR of 100%. Considering the high antitumor activity and the tolerable safety profile observed at this dose level, it was decided that no further dose escalation would be conducted. On this basis, surufatinib 200 mg was determined to be the RP2D. Surufatinib 200 mg showed manageable safety and high antitumor activity.

### Phase II randomized study: efficacy and safety

#### Baseline characteristics

**Fig. 1:**
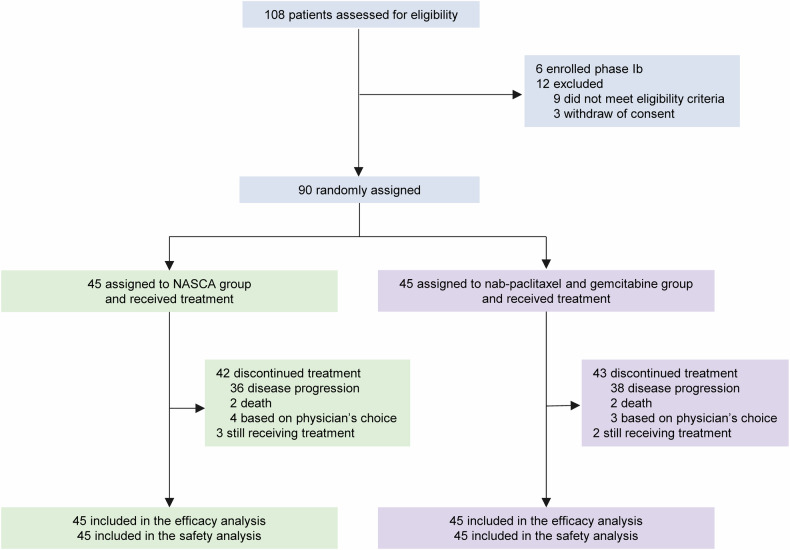
Trial profile. Two deaths occurred in the NASCA group, one due to liver failure and the other due to unspecified causes. In the nab-paclitaxel and gemcitabine group, one death was attributed to an infection and one due to unspecified causes

During the phase II trial period from December 2021 to June 2024, 90 patients were randomly assigned: 45 to receive surufatinib in combination with camrelizumab, nab-paclitaxel, and S-1 (the NASCA group), and 45 to the group treated with nab-paclitaxel and gemcitabine. All randomized subjects were administered their allocated regimens (Fig. 1). Additionally, 35.6% of NASCA and 35.6% of nab-paclitaxel and gemcitabine patients had one metastatic site, and 35.6% and 31.1% had two, respectively. Biliary drainage was performed prior to treatment in seven individuals (15.6%) from the NASCA group and four (8.9%) from the nab-paclitaxel and gemcitabine group. In addition, one patient in the latter group required biliary drainage while on therapy (Table 1). Baseline demographics and clinical features were balanced between groups.

**Table 1:** Baseline characteristics

	NASCA (N = 45)	Nab-paclitaxel and gemcitabine (N = 45)
Sex, n (%)		
Male	30 (66.7)	25 (55.6)
Female	15 (33.3)	20 (44.4)
Age (years)		
Median (IQR)	59.0 (56.0, 61.0)	59.0 (55.0, 66.0)
Range	36.0–75.0	39.0–74.0
ECOG performance status, n (%)		
0	18 (40.0)	14 (31.1)
1	27 (60.0)	31 (68.9)
Differentiation grade, n (%)		
Undifferentiated	11 (24.4)	7 (15.6)
Poorly-differentiated	25 (55.6)	29 (64.4)
Moderately-differentiated	7 (15.6)	9 (20.0)
Well-differentiated	2 (4.4)	0
Primary tumor location, n (%)		
Head	18 (40.0)	19 (42.2)
Body/Tail	27 (60.0)	26 (57.8)
Stage, n (%)		
Metastatic disease	37 (82.2)	36 (80.0)
Locally advanced	8 (17.8)	9 (20.0)
Sites of metastasis, n (%)		
Liver	23 (51.1)	25 (55.6)
lymph nodes	24 (53.3)	20 (44.4)
Peritoneum	9 (20.0)	9 (20.0)
Lung	5 (11.1)	8 (17.8)
Other	3 (6.7)	3 (6.7)
Metastatic sites, n (%)		
1	16 (35.6)	16 (35.6)
2	16 (35.6)	14 (31.1)
3	5 (11.1)	6 (13.3)
Prior resection of primary site		
Yes	6 (13.3)	9 (20.0)
No	39 (86.7)	36 (80.0)
Baseline CA 19-9, U/mL		
Median (IQR)	290.0 (47.4, 1635.0)	518.0 (98.5, 2998.0)
Baseline CEA, ng/mL		
Median (IQR)	4.6 (2.5, 8.1)	3.6 (2.2, 12.6)
Prior biliary drainage, n (%)	7 (15.6)	4 (8.9)

#### Efficacy

At data cutoff on March 3, 2025, patients had been followed for a median of 21.5 months (95% confidence interval [CI]: 17.5–25.9). The ORR was 51.1% (23/45) (95% CI 35.8–66.3) in the NASCA and 24.4% (11/45) (95% CI 12.9–39.5) in the nab-paclitaxel and gemcitabine groups (rate difference: 26.7%, 90% CI 9.9–41.5 and 95% CI 6.6–43.9; odds ratio [OR]: 3.2, 95% CI 1.3–8.2, *p* = 0.01). The DCR reached 91.1% (95% CI 78.8–97.5) and 88.9% (95% CI 75.9–96.3), respectively. Partial response (PR) was achieved by 23 patients and 11, while stable disease (SD) was observed in 18 and 29 patients in the two groups, respectively (Table 2 and supplementary Fig. 1). Patients receiving NASCA had a median duration of response (DOR) of 8.1 months (95% CI: 5.8–13.3), while those treated with nab-paclitaxel and gemcitabine had a median DOR of 6.0 months (95% CI: 2.3–13.0). The median time to response (TTR) for NASCA was 1.6 months (95% CI: 1.5–2.7), notably shorter than the 2.5 months (95% CI: 1.4–3.8) observed in the comparator group (Table 2).

**Table 2:** Tumor response

Tumor response	NASCA (N = 45)	Nab-paclitaxel and gemcitabine (N = 45)
Partial response, n (%)	23 (51.1)	11 (24.4)
Stable disease, n (%)	18 (40.0)	29 (64.4)
Progressive disease, n (%)	4 (8.9)	5 (11.1)
Objective response rate, (95% CI)	51.1% (35.8–66.3)	24.4% (12.9–39.5)
Disease control rate, (95% CI)	91.1% (78.8–97.5)	88.9% (75.9–96.3)
Time to response (months), median (95% CI)	1.6 (1.5–2.7)	2.5 (1.4–3.8)
Duration of response (months), median (95% CI)	8.1 (5.8–13.3)	6.0 (2.3–13.0)

**Fig. 2:**
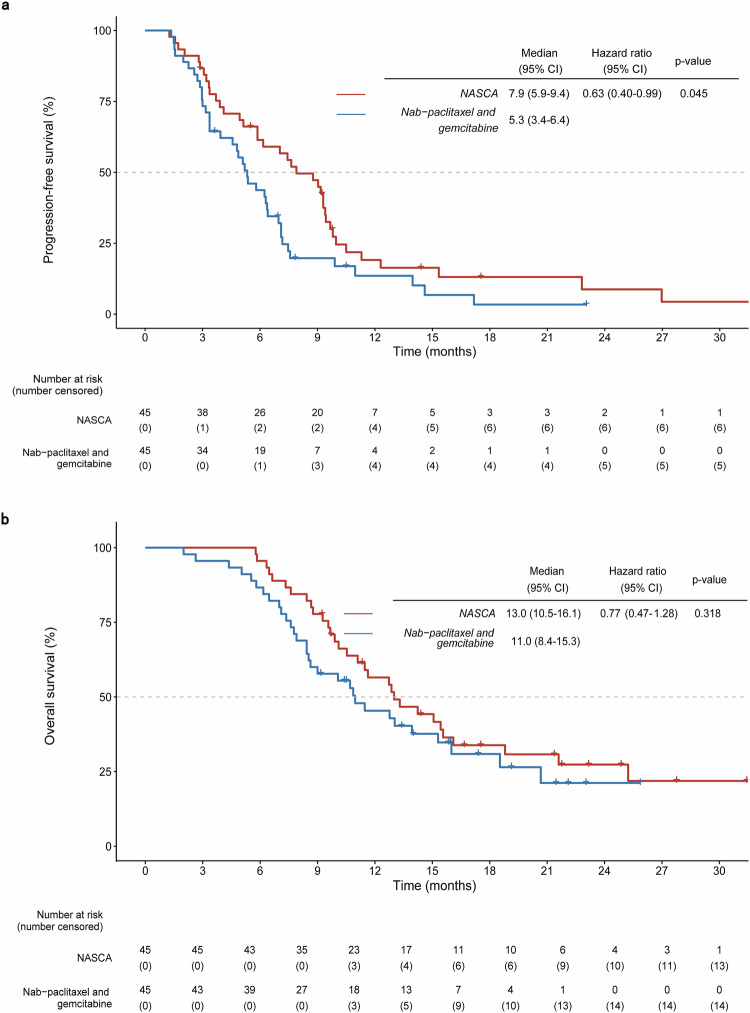
Kaplan‒Meier analyses of survival in the intention-to-treat population as assessed by investigators. **a** Kaplan‒Meier curves of progression-free survival (%). **b** Kaplan‒Meier curves of overall survival

Patients in the NASCA group experienced a median PFS of 7.9 months (95% CI: 5.9–9.4), as opposed to 5.3 months (95% CI: 3.4–6.4) for those receiving nab-paclitaxel and gemcitabine. The calculated hazard ratio (HR) was 0.63 (95% CI: 0.40–0.99; *p* = 0.045). The 6-month and 12-month PFS rates were 61.4% (95% CI 45.4–74.0) versus 43.7% (95% CI 28.9–57.6) and 19.1% (95% CI 8.7–32.6) versus 13.5% (95% CI 5.0–26.3), respectively. Furthermore, the median OS reached 13.0 months (95% CI: 10.5–16.1) for the NASCA and 11.0 months (95% CI: 8.4–15.3) for the nab-paclitaxel and gemcitabine (HR: 0.77; 95% CI: 0.47–1.28; *p* = 0.318). Six- and 12-month OS rates were 95.6% (95% CI: 83.4–98.9) and 56.5% (95% CI: 40.6–69.7) for NASCA, versus 86.7% (95% CI: 72.7–93.8) and 45.4% (95% CI: 30.2–59.4) for the comparator group (Fig. 2). The NASCA regimen achieved a higher ORR and prolonged PFS compared to nab-paclitaxel and gemcitabine group, with a trend toward improved OS.

At the data cutoff, 42 (93.3%) patients in the NASCA group and 43 (95.6%) in the nab-paclitaxel and gemcitabine group had discontinued study treatments. Subsequent therapy was received by 28 (66.7%) and 37 (86.0%) patients, respectively. The most common treatments were chemotherapy, mainly irinotecan (17 [60.7%] vs. 14 [37.8%]) or liposomal irinotecan (10 [35.7%] vs. 16 [43.2%]) and oxaliplatin (19 [67.9%] vs. 27 [73.0%]). Details of other therapies are provided in Supplementary Tables 1 and 2.

#### Safety

To evaluate tolerability, treatment-emergent adverse events (TEAEs) were assessed in both groups. In the NASCA group, 45 patients (100%) experienced TEAEs, compared to 43 patients (95.6%) receiving nab-paclitaxel and gemcitabine. Hematologic AEs, particularly declines in white blood cell, neutrophil, and lymphocyte counts, were the predominant TEAEs in patients treated with NASCA. For those receiving nab-paclitaxel and gemcitabine, decreased white blood cell count, anemia, and dcreased neutrophil count were most frequently reported. In the NASCA group, the most prevalent Grade 3 and 4 TEAEs were declines in white blood cell, neutrophil, and lymphocyte counts, whereas decreased white blood cell and neutrophil counts were prominent in the nab-paclitaxel and gemcitabine group (Table 3). Additionally, immune-related adverse events (irAEs) were documented in ten (22.2%) patients in the NASCA group, including three cases of Grade 2 hepatitis, one case of Grade 3 hepatitis, one case of Grade 2 colitis, two cases of Grade 2 hypothyroidism, two cases of Grade 2 hypophysitis, and one case of Grade 3 rash. Dose reductions due to TEAEs were required in seven patients (15.6%) in the NASCA and nine (20.0%) in the nab-paclitaxel and gemcitabine groups. Treatment discontinuation of any medication occurred in 15 patients (33.3%) receiving NASCA and 10 patients (22.2%) receiving nab-paclitaxel and gemcitabine. Two deaths occurred in the NASCA group, one due to liver failure not related to treatment and one from unspecified causes. For treatment with nab-paclitaxel and gemcitabine, one death was attributed to a non-treatment-related infection and one from unspecified causes. NASCA treatment was associated with manageable TEAEs, with no new safety signals.

**Table 3:** Treatment-emergent adverse event in the safety population

	NASCA group				Nab-paclitaxel and gemcitabine group			
	(N = 45)				(N = 45)			
	All grades	Grade 1–2	Grade 3	Grade 4	All grades	Grade 1–2	Grade 3	Grade 4
Decreased white blood cell count	34 (75.5%)	20 (44.4%)	10 (22.2%)	4 (8.9%)	33 (73.3%)	21 (46.7%)	8 (17.8%)	4 (8.9%)
Decreased neutrophil count	33 (73.3%)	18 (40.0%)	9 (20.0%)	6 (13.3%)	27 (60.0%)	11 (24.4%)	12 (26.7%)	4 (8.9%)
Decreased lymphocyte count	22 (48.9%)	13 (28.9%)	8 (17.8%)	1 (2.2%)	14 (31.1%)	11 (24.4%)	3 (6.7%)	0
Anemia	21 (46.7%)	20 (44.4%)	1 (2.2%)	0	31 (68.9%)	30 (66.7%)	1 (2.2%)	0
Decreased platelet count	21 (46.7%)	20 (44.4%)	1 (2.2%)	0	20 (44.4%)	16 (35.6%)	3 (6.7%)	1 (2.2%)
Alopecia	21 (46.7%)	21 (46.7%)	0	0	18 (40.0%)	18 (40.0%)	0	0
Peripheral neuropathy	20 (44.4%)	15 (33.3%)	4 (8.9%)	1 (2.2%)	9 (20.0%)	9 (20.0%)	0	0
Anorexia	20 (44.4%)	20 (44.4%)	0	0	18 (40.0%)	18 (40.0%)	0	0
Nausea	19 (42.2%)	19 (42.2%)	0	0	22 (48.9%)	22 (48.9%)	0	0
Vomiting	13 (28.9%)	13 (28.9%)	0	0	11 (24.4%)	11 (24.4%)	0	0
Increased alanine aminotransferase	16 (35.6%)	15 (33.3%)	1 (2.2%)	0	16 (35.6%)	15 (33.3%)	1 (2.2%)	0
Increased aspartate aminotransferase	16 (35.6%)	13 (28.9%)	3 (6.7%)	0	17 (37.8%)	16 (35.6%)	1 (2.2%)	0
Elevated bilirubin	14 (31.1%)	9 (20.0%)	5 (11.1%)	0	7 (15.6%)	5 (11.1%)	1 (2.2%)	1 (2.2%)
Fatigue	11 (24.4%)	11 (24.4%)	0	0	9 (20.0%)	7 (15.6%)	2 (4.4%)	0
Diarrhea	6 (13.3%)	5 (11.1%)	1 (2.2%)	0	4 (8.9%)	4 (8.9%)	0	0
Rash	5 (11.1%)	4 (8.9%)	1 (2.2%)	0	3 (6.7%)	3 (6.7%)	0	0
Abdominal pain	2 (4.4%)	2 (4.4%)	0	0	3 (6.7%)	3 (6.7%)	0	0
Albuminuria	2 (4.4%)	2 (4.4%)	0	0	0	0	0	0
Hypothyroidism	2 (4.4%)	2 (4.4%)	0	0	0	0	0	0

#### Subgroup and biomarker analyses

Subgroup analyses for both PFS and OS demonstrated consistent patterns with the results observed in the primary analyses (Supplementary Figs. 2–4). In both the NASCA and nab-paclitaxel and gemcitabine groups, patients with a CA19-9 decrease at week 6 or week 12 had longer median PFS compared to those without a decrease (Supplementary Table 3).

**Fig. 3:**
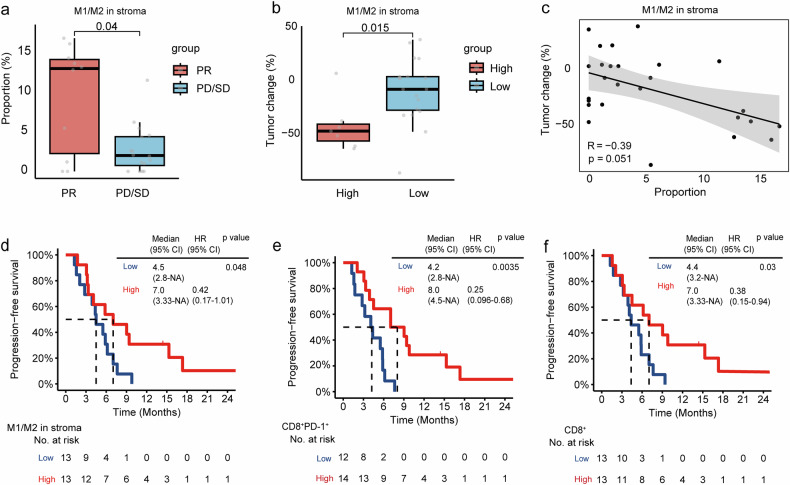
Fluorescent multiplex immunohistochemistry analysis of baseline tissue samples from 26 patients who received NASCA treatment. **a** Comparison of the ratio of M1/M2 macrophage percentages in the stroma between patients with partial response (PR) and those with stable disease (SD) or progressive disease (PD). **b** Tumor shrinkage in patients with a higher ratio of M1/M2 macrophage percentages in the stroma compared to those with a lower ratio, using a cutoff value of 10. **c** Correlation between the ratio of M1/M2 macrophage percentages in the stroma and tumor shrinkage. **d** Progression-free survival was analyzed according to the ratio of M1 to M2 macrophage percentages, with patients divided into high and low groups based on the median value. **e** Progression-free survival was analyzed based on the levels of CD8^+^ cells, using the median as the cutoff to define high and low groups. **f** Progression-free survival was analyzed in relation to the levels of CD8^+^PD-1^+^ cells, stratified by the median value. HR Hazard Ratio, NA not available

Multiplex immunohistochemistry (mIHC) was performed on baseline tissue samples of 26 patients who received NASCA treatment. The proportion of M1 to M2 macrophages within the stroma was markedly elevated in individuals achieving PR relative to those with SD and progressive disease (PD) (*p* = 0.04) (Fig. 3a). Using an M1/M2 cutoff of 10, patients with higher M1/M2 ratios exhibited greater tumor shrinkage compared to those with lower ratios (Fig. 3b). Additionally, stromal M1/M2 cell percentages showed a correlation with the magnitude of tumor shrinkage. (r = −0.39, *p* = 0.051) (Fig. 3c). Using the median as a cutoff, patients with higher levels of M1/M2 (*p* = 0.048), CD8^+^ (*p* = 0.03), and CD8^+^PD-1^+^ cells (*p* = 0.0035) in stroma had longer PFS compared to those with lower levels (Fig. 3d–f). Immune microenvironment features, including M1/M2 ratio and CD8^+^ infiltration, may serve as predictive biomarkers for NASCA benefit.

## Discussion

Management of advanced pancreatic cancer continues to be challenging, largely owing to scarce treatment choices and dismal outcomes. Our study evaluates a new first-line regimen, combining surufatinib, camrelizumab, nab-paclitaxel, and S-1, and confirms its efficacy and safety in patients with metastatic pancreatic adenocarcinoma. The NASCA group achieved an ORR of 51.1%, significantly exceeding that of the standard nab-paclitaxel and gemcitabine regimen (24.4%), thereby meeting the primary endpoint. Additionally, the NASCA group showed a longer median PFS (7.9 months) relative to the nab-paclitaxel and gemcitabine group (5.3 months). Notably, this study represents the first randomized trial comparing anti-PD-1 therapy combined with anti-angiogenic therapy and chemotherapy versus chemotherapy alone in this setting. Our findings align with those from a similar phase II study evaluating anti-PD-1 therapy and angiogenesis inhibitors with chemotherapy in metastatic pancreatic cancer. Penpulimab with anlotinib and gemcitabine and nab-paclitaxel achieved an ORR of 50.0%, with a median PFS and OS of 8.8 and 13.7 months, respectively.^15^ Additionally, our ORR and PFS values were numerically higher than those observed with nivolumab and modified FOLFIRINOX according to phase II trial data, which reported an ORR of 32.3% and a median PFS of 7.4 months.^9^ The NASCA group also showed higher ORR and PFS compared to the gemcitabine and nab-paclitaxel in the MPACT and NAPOLI-3 studies, which reported ORRs of 23% and 36%, with a median PFS of 5.5 and 5.6 months, respectively.^4,6^ Nonetheless, it is essential to approach these cross-study comparisons cautiously, as differences in study designs and patient populations limit direct comparisons.

Mechanistically, these results of surufatinib may be attributed to its ability to improve tumor vasculature normalization at a relatively low dose.^13,14^ This improvement facilitates better drug delivery and mitigates hypoxia-induced chemotherapy resistance.^11,36^ Surufatinib, a VEGFR tyrosine kinase inhibitor, also targets CSF1R. Blocking CSF1R signaling decreases the infiltration of tumor-associated macrophages and limits their polarization toward the M2 phenotype.^37^ Notably, in vitro studies have shown that surufatinib plus an anti-PD-1 antibody and nab-paclitaxel and gemcitabine downregulates CSF1R, CD163 (an M2 macrophage marker), and PD-L1.^38^ Through its influence on the CSF-1/CSF-1R signaling axis, surufatinib could contribute to reshaping the immune contexture within tumors, which may improve responses to combination therapies. This study found that a higher baseline M1/M2 macrophage ratio was significantly associated with a higher response rate, deeper responses, as well as improved PFS. Moreover, CA19-9 has been recognized as a biomarker for predicting treatment response in several large phase III trials.^39^ In this trial, patients with any decrease in CA19-9 at weeks 6 and 12 had a better PFS than those with no decrease, regardless of whether they received the NASCA or nab-paclitaxel and gemcitabine.

In the MPACT and NAPOLI-3 phase III trials, median OS for patients treated with nab-paclitaxel and gemcitabine was 8.5 and 9.2 months, respectively,^4,6^ while this study demonstrated a median OS of 11.0 months. The longer OS observed in this study may be attributed to the availability of subsequent therapies. Specifically, 86.0% of patients treated with the nab-paclitaxel and gemcitabine group and 66.7% treated with NASCA received subsequent therapy. At our center, we improved the availability of irinotecan-based regimens for second-line therapy, guided by findings from the phase III HR-IRI-APC trial in which liposomal irinotecan plus 5-fluorouracil and leucovorin as second-line treatment yielded a median OS of 7.39 months.^40^ Safety profile of the nab-paclitaxel and gemcitabine in this study aligned with data from the MPACT and NAPOLI-3 trials.^4,6^ Moreover, the addition of surufatinib and camrelizumab to the nab-paclitaxel and S-1 backbone reported a controllable safety profile. The administration of two oral drugs led to more pronounced gastrointestinal discomfort in the NASCA group. Furthermore, the incidences of Grade 3 increased aspartate aminotransferase and elevated bilirubin were higher in the NASCA group, which may be attributable to surufatinib and camrelizumab. Notably, around 11.1% of patients receiving NASCA developed Grade 3 or 4 peripheral neuropathy, an adverse effect that was not reported in those given nab-paclitaxel and gemcitabine. Although this difference may reflect regimen-specific toxicity, the small sample size warrants cautious interpretation.

According to the NCCN guidelines, first-line treatment options for locally advanced PDAC include enrollment in clinical trials, systemic therapy, chemoradiotherapy, or stereotactic body radiation therapy. Among this study participants, locally advanced disease was observed in 20.0% of those receiving nab-paclitaxel and gemcitabine and in 17.8% of those treated with the NASCA regimen. Although the proportion of these patients was relatively small and balanced between arms, their inclusion may have introduced clinical heterogeneity, as locally advanced and metastatic PDAC differ in prognosis and treatment response. This heterogeneity could potentially influence the interpretation and generalizability of the efficacy outcomes. Notably, patients with locally advanced disease who received the NASCA regimen appeared to have improved survival outcomes. Nevertheless, the reliability of this observation is constrained by the small number of patients in this subgroup, underscoring the need for further research. Additionally, this investigation was limited to a single center with a modest sample size, involving only Chinese participants, which may affect both the statistical robustness and the broader applicability of the results. Although the control group received the standard regimen of nab-paclitaxel and gemcitabine, the experimental group did not simply build upon this backbone by adding surufatinib and camrelizumab. The decision to use S-1 instead of gemcitabine with nab-paclitaxel was guided by the demonstrated tolerability of the nab-paclitaxel plus S-1 regimen, in addition to our center’s prior successful experience with this combination. This design complicates the attribution of the observed clinical benefit specifically to surufatinib and camrelizumab. Moreover, the findings remain preliminary and are not definitive. Nevertheless, this exploratory study provides an early signal that the addition of a PD-1 inhibitor and an anti-angiogenic agent to chemotherapy may offer therapeutic benefit in PDAC, in contrast to the limited efficacy reported in prior studies of chemoimmunotherapy. A phase II/III randomized controlled trial is currently being conducted to examine the combined use of surufatinib, camrelizumab, nab-paclitaxel, and gemcitabine for additional validation of these findings.

In conclusion, the NASCA regimen demonstrated superior outcomes compared to the nab-paclitaxel and gemcitabine regimen regarding ORR, TTR, and PFS, while maintaining an acceptable safety profile in the management of PDAC. Enrichment of CD8^+^ and CD8^+^PD-1^+^ cells, a high M1/M2 macrophage ratio at baseline, and an early reduction in CA19-9 levels served as valuable predictive biomarkers for PFS, underscoring their potential utility in clinical practice.

## Materials and methods

### Study design and participants

Ethical approval for the protocol was granted by the Ethics Committee of the Chinese PLA General Hospital (S2021-228-03), and the study is registered on ClinicalTrials.gov (NCT05218889). Written informed consent was obtained from all subjects prior to participation. The trial was conducted in accordance with the Declaration of Helsinki and Good Clinical Practice recommendations.

This investigation was designed as a phase Ib/II randomized clinical trial. Eligible individuals ranged from 18 to 75 years old and had pathologically or cytologically confirmed unresectable, locally advanced, or metastatic PDAC without previous systemic treatment for advanced disease. Principal inclusion criteria involved an Eastern Cooperative Oncology Group (ECOG) performance status between 0 and 1, at least one target lesion assessable by RECIST version 1.1, sufficient function of vital organs, and a projected life expectancy of at least 3 months. Exclusion parameters encompassed a history of treatment with VEGFR inhibitors or prior administration of immune checkpoint blockade.

### Procedures

During the phase Ib, dose-escalation portion of the study, surufatinib was given orally once daily at increasing dose levels of 200, 250, or 300 mg. This agent was administered in combination with camrelizumab, nab-paclitaxel, and S-1. Camrelizumab was dosed intravenously (IV) at 200 mg on day 1 of each 21-day treatment cycle. Nab-paclitaxel was infused IV at 125 mg/m^2^ on days 1 and 8, while S-1 was taken orally at 40 mg twice daily from days 1 to 14 of each three-week cycle.

Dose escalation followed a 3 + 3 design based on DLTs observed in the first treatment cycle. Non-hematologic DLTs were defined as Grade ≥3 toxicities, except for certain conditions that were excluded: Grade 3 nausea, vomiting, diarrhea, or constipation resolving within one week after supportive care; Grade 3 fatigue lasting ≤7 days; Grade 3 hypertension controllable within one week; alopecia, fever, or alkaline phosphatase elevation attributable to tumor or infection; and Grade ≥3 decrease in ventricular ejection fraction. Hematologic DLTs included Grade 4 neutropenia, thrombocytopenia, or hemoglobin decrease confirmed by at least two tests within 2 days; Grade 3 thrombocytopenia with bleeding tendency confirmed by at least 2 tests within 2 days; and Grade 3 dcreased neutrophil count with fever (ANC < 1.0 × 10^9^/L and body temperature ≥ 38.5 °C), confirmed at least twice within 2 days despite supportive care.

In the phase II segment, participants were allocated in a 1:1 ratio to receive either the NASCA regimen or nab-paclitaxel in combination with gemcitabine, utilizing block randomization with a block size of four and no stratification parameters. The random allocation sequence was generated by an independent biostatistician using R software (v4.4.1), and both the randomization sequence and block size were concealed from investigators to ensure allocation concealment until intervention assignment. Randomization was performed immediately prior to treatment initiation, and once assigned, randomization numbers were irrevocable and not reassigned.

Patients in the NASCA group received surufatinib at the RP2D in conjunction with camrelizumab, nab-paclitaxel, and S-1 (same regimens as phase Ib) for eight cycles. Patients demonstrating a complete response (CR), PR, or SD then received maintenance therapy consisting of surufatinib (RP2D), S-1, and camrelizumab (same regimens as phase Ib). In the nab-paclitaxel and gemcitabine cohort, treatment involved nab-paclitaxel at 125 mg/m^2^ IV on days 1 and 8, together with gemcitabine at 1000 mg/m^2^ IV over 30 min on the same days, repeated every 21 days. Therapy in both study arms continued until radiographic progression, unacceptable toxicity, consent withdrawal, or patient death. Tumor evaluations were performed every 6 weeks according to RECIST version 1.1 guidelines. AEs were categorized based on the National Cancer Institute Common Terminology Criteria for Adverse Events (NCI-CTCAE), version 5.0. Survival status was monitored every three months in the first year after study drug discontinuation, and followed thereafter until death, loss to follow-up, or withdrawal of consent. Methodological details regarding fluorescent multiplex immunohistochemistry and imaging techniques are provided in the supplementary appendix.

### Outcomes

The primary endpoints were DLTs and the RP2D of surufatinib in phase Ib, and ORR in phase II. Secondary endpoints included DCR, DOR, TTR, PFS, OS, and safety. Efficacy was analyzed in the intention-to-treat population, which encompassed all patients who were randomized. For the safety analysis, all individuals who were randomized and received at least one dose of a study medication were included. As per RECIST version 1.1 criteria, ORR was defined as the percentage of subjects with either CR or PR, while DCR included those with CR, PR, or SD. DOR represented the interval from the initial CR or PR documentation until disease progression or death. TTR was measured from randomization to the earliest achievement of CR or PR. PFS was calculated from randomization to the point of disease progression or death, and OS was measured from randomization until death due to any cause.

### Statistical analysis

Sample size estimation was based on an anticipated ORR of 0.49 for the NASCA group and 0.23 for the nab-paclitaxel and gemcitabine group, with a superiority margin set at 0. The calculation assumed a one-sided alpha level of 0.05 and a beta of 0.2. With equal randomization (1:1 ratio) between the two treatment groups, 40 participants per group were needed, equating to a total of 80 subjects. Accounting for a projected dropout rate of 10%, the final required sample size was determined to be 90 patients.

The median duration of follow-up was estimated using the reverse Kaplan–Meier approach. For the calculation of 95% CIs for ORR and DCR, the Clopper–Pearson exact method was applied. To determine the difference in rates between the experimental and control arms, along with associated two-sided 90% CIs, the Newcombe-Wilson method was used. Superiority was concluded if the lower limit of the 90% CI for the rate difference was greater than zero. OR was calculated by univariate logistic regression analysis. Median PFS and OS were estimated using the Kaplan–Meier technique, while survival differences between groups were assessed with the log-rank test. HRs and their 95% CIs were calculated using the Cox proportional hazards regression model. To identify the optimal threshold of serum CA 19-9 for predicting 6-month survival, a time-dependent ROC curve analysis was conducted. A 6-week decrease or a 12-week decrease of CA19-9 levels were analyzed according to the optimal cutoff values. Subgroup analyses were conducted based on baseline variables and CA19-9 levels (by decrease using the optimal cutoff value). The evaluations employed the Cox proportional hazards method and were summarized graphically using forest plots. Differences in immune cell populations among patients showing PR versus those with SD or PD were assessed using the Wilcoxon rank-sum test. The Spearman correlation was assessed between immune cell subsets and the best tumor shrinkage. All statistical analyses were conducted utilizing R software (version 4.4.1).

## Supplementary information

Supplementary information

Supplementary information

Supplementary information

Supplementary information

Supplementary information

## Data Availability

The protocol, statistical analysis plan, and other relevant study materials are publicly available online. The datasets generated and analyzed during the current study are not publicly available due to ethical and regulatory restrictions but may be made available from the corresponding authors upon reasonable request. De-identified participant data may be shared following approval of a proposal that includes a detailed description of study objectives and a statistical analysis plan, which will be reviewed by all corresponding authors.
