# Supplementary material for: First-line treatment with chemotherapy, surufatinib (an angio-immuno kinase inhibitor), and camrelizumab (an anti-PD-1 antibody) for locally advanced or metastatic pancreatic ductal adenocarcinoma: a phase Ib/II randomized study
Source: Signal Transduct Target Ther. 2025 Oct 13;10:339. doi: 10.1038/s41392-025-02441-2 (PMC12515981; doi:10.1038/s41392-025-02441-2)
Supplement: Supplementary file 1 — Supplementary information [file 41392_2025_2441_MOESM1_ESM.docx]

Supplementary Materials for

First-line treatment with chemotherapy, surufatinib (an angio-immuno kinase inhibitor), and camrelizumab (an anti-PD-1 antibody) for locally advanced or metastatic pancreatic ductal adenocarcinoma: a phase Ib/II randomized study

Ru Jia, Hai-Yan Si, Meng-Jiao Fan, Nan Zhang, Guo-Chao Deng, Fang-Fang Liu, Lu Han, Miao-Miao Gou, Zhao-Li Tan, Xia Zhang, Yan-Rong Wang, Yue Shi, Yao-Yue Zhang, Yu-Shan Jia, Yu-Qi Wang, Quan-Li Han, Zhi-Kuan Wang, Guang-Hai Dai

Correspondence to: daigh301@vip.sina.com; [wangzkme@sohu.com](mailto:wangzkme@sohu.com); hanquanli@aliyun.com

**This PDF file includes:**

Materials and Methods

Figures. S1 to S4

Tables S1 to S4

**Fluorescent Multiplex Immunohistochemistry Staining and Imaging**

Primary antibodies targeting CD163, CD68, PD-1, PD-L1, CD3, CD4, CD8, CD56, CD20, Foxp3, and pan-cytokeratin (pan-CK) were sequentially applied to formalin-fixed, paraffin-embedded tissue sections (supplemental Table 3). After incubation with secondary antibodies and corresponding Opal fluorophores, as well as DAPI, the stained slides were scanned using the Vectra Polaris Quantitative Pathology Imaging System (Akoya Biosciences, USA). Image analysis was conducted with APTIME software by 3D Medicines. Tumor parenchyma and stroma were differentiated using CK staining.

The density of immune cell subsets was quantified as the number of positively stained cells per mm². Subsets in both tumor and stromal regions were identified by detecting specific marker signals, including CD3^+^, CD3^+^CD4^+^, CD8^+^, Foxp3^+^, PD-1^+^CD8^+^, CD68^+^CD163^-^ (M1 macrophages), CD68^+^CD163^+^ (M2 macrophages), PD-L1^+^CD68^+^, CD56 bright NK cells, and CD56 dim NK cells. The co-localization of CD3^+^ T cells and CD20^+^ B cells indicated the presence of tertiary lymphoid structures.


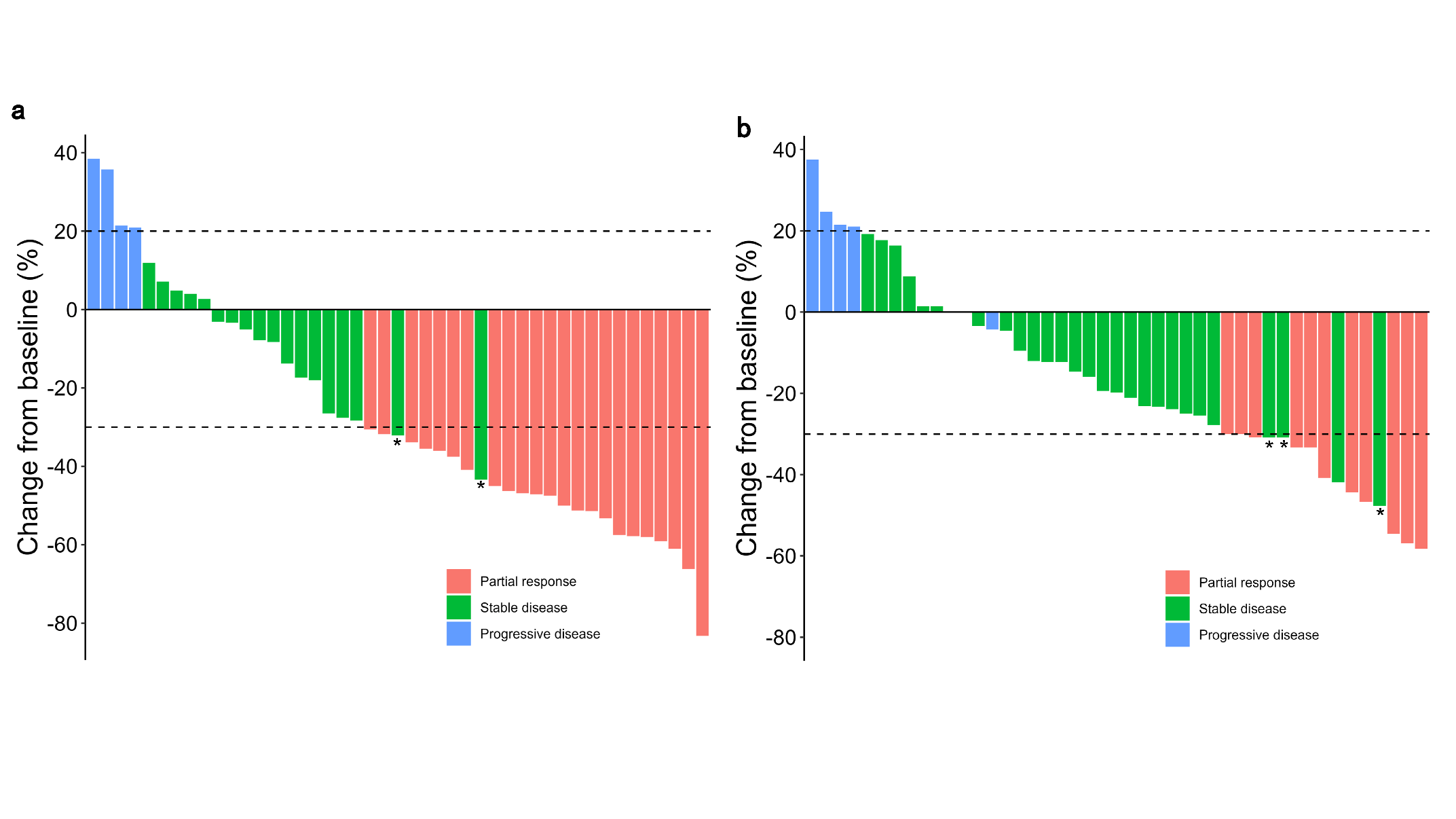
**Figure. S1.**

**Waterfall plot of tumor response in NASCA (a) and nab-paclitaxcel and gemcitabine (b).**

^*^Patients with partial response were not confirmed and were deemed to have stable disease.


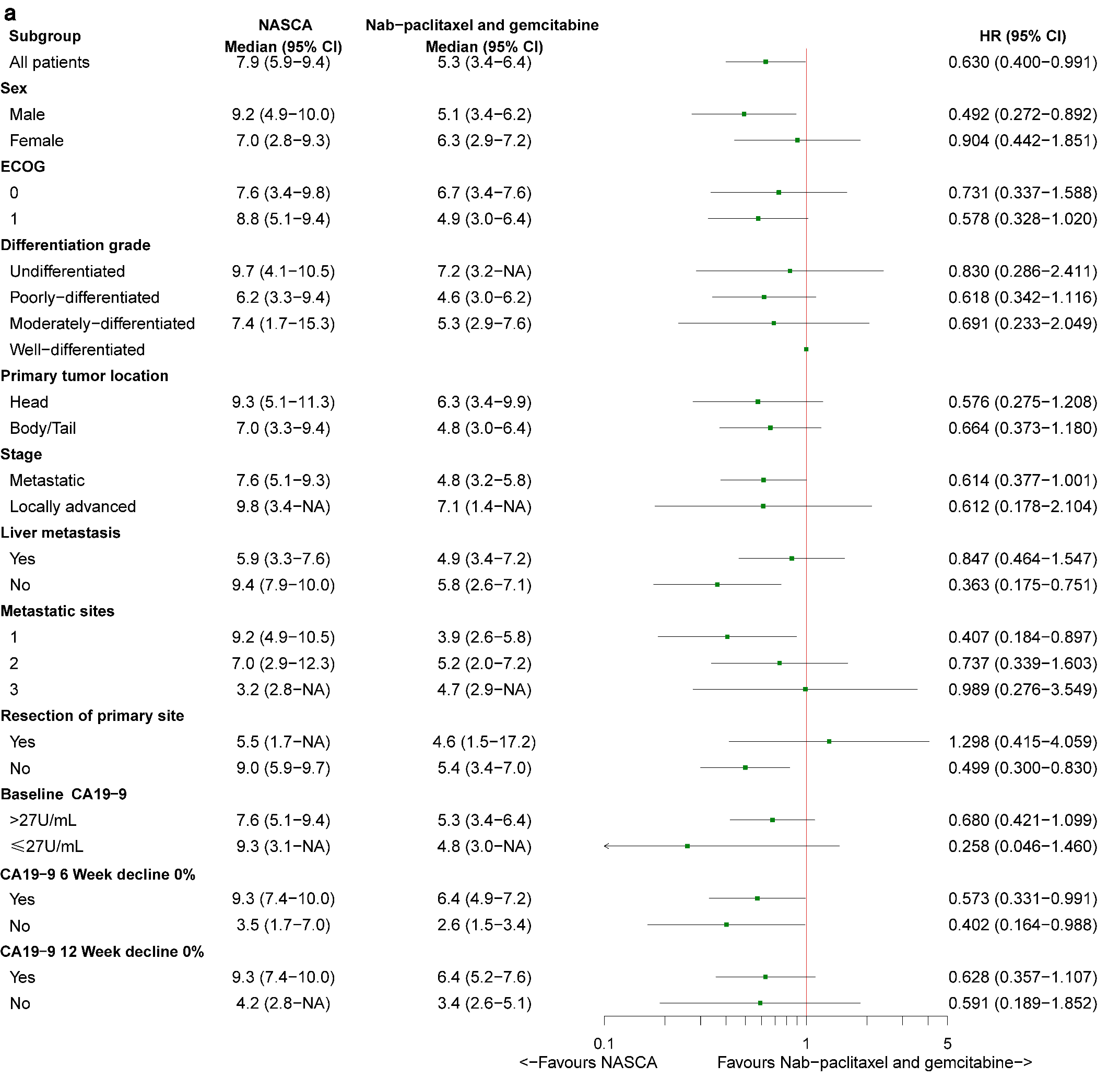


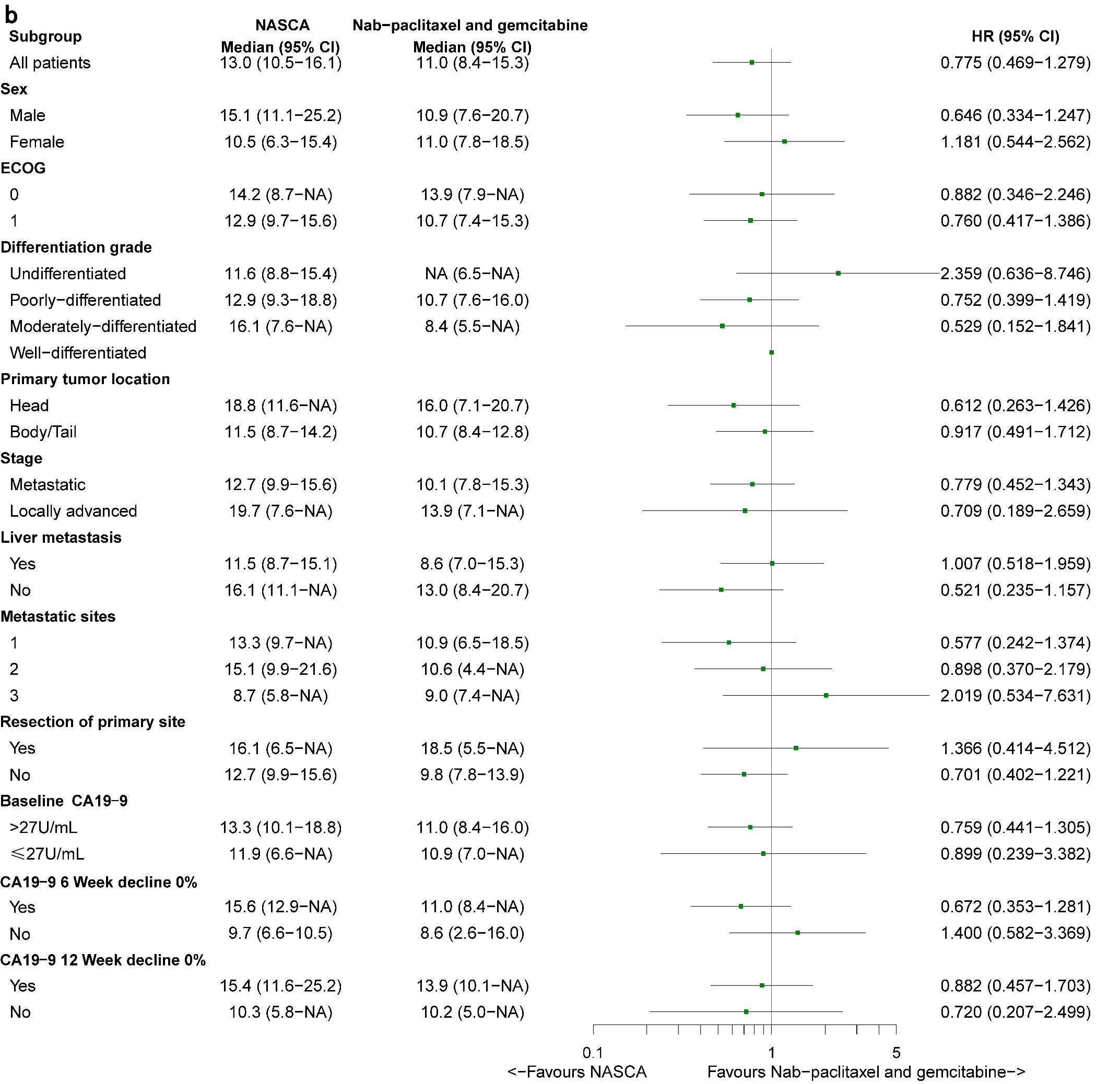


**Figure. S2.**

**Forest plot of progression-free survival (a) and overall survival (b).**

^*^Our center's CA19-9 normal value cutoff was 27 U/mL. *NA* not available.


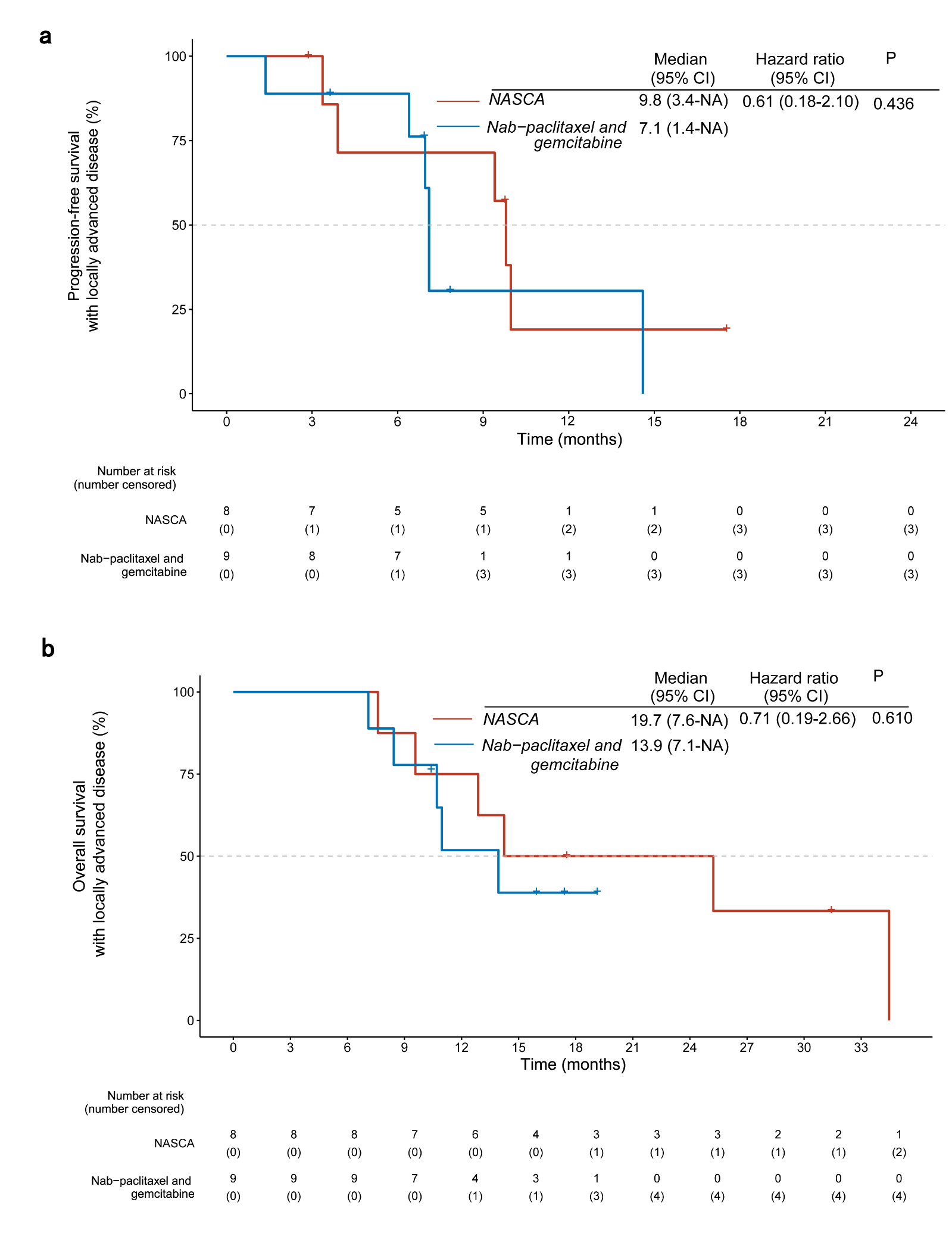


**Figure. S3.**

**Kaplan-Meier estimates of progression-free survival (a) and overall survival (b) in patients with locally advanced disease.**

*NA* not available.


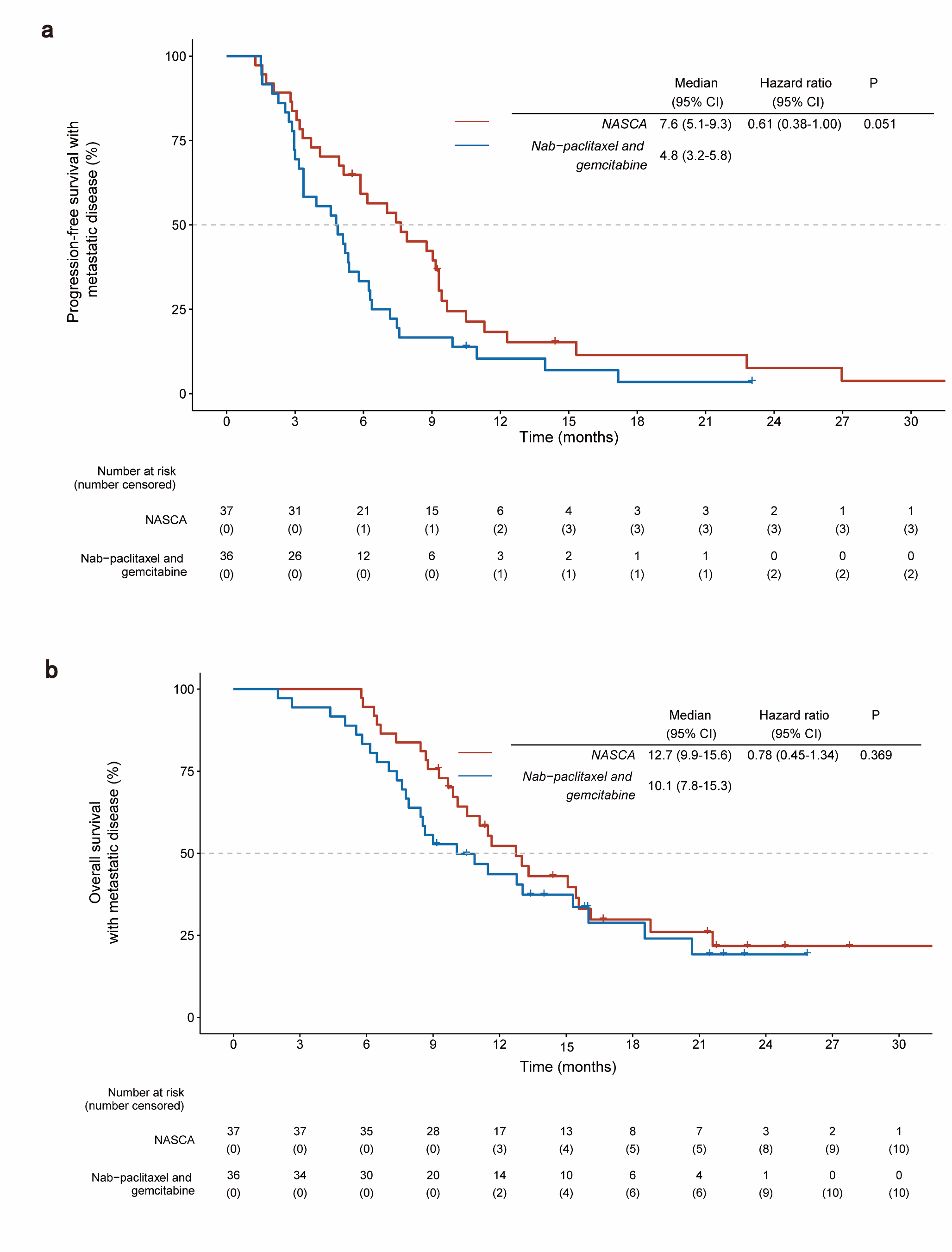
**Figure. S4.**

**Kaplan-Meier estimates of progression-free survival (a) and overall survival (b) in patients with metastatic disease.Table S1. Subsequent anti-cancer therapy.**

| Subsequent anti-cancer therapy, n (%) | NASCA (N=28) | Nab-paclitaxel and gemcitabine (N=37) |
| --- | --- | --- |
| **Chemotherapy** | 26 (92.9%) | 33 (89.2%) |
| Irinotecan | 17 (60.7%) | 14 (37.8%) |
| liposomal irinotecan | 10 (35.7%) | 16 (43.2%) |
| Oxaliplatin | 19 (67.9%) | 27 (73.0%) |
| Gemcitabine | 11 (39.3%) | 1 (2.7%) |
| Other | 14 (50.0%) | 18 (48.6%) |
| **Targeted therapy** |  |  |
| Nimotuzumab | 2 (7.1%) | 1 (2.7%) |
| Bevacizumab | 17 (60.7%) | 22 (59.5%) |
| PARPi | 2 (7.1%) | 2 (5.4%) |
| **Anti-PD-1 antibody** | 6 (21.4%) | 7 (18.9%) |
| **Radiotherapy** | 7 (25.0%) | 5 (13.5%) |

Table S2. Second-line chemotherapy treatment.

|  | NASCA (N=26) | | Nab-paclitaxel and gemcitabine (N=33) | |
| --- | --- | --- | --- | --- |
| **Second-line treatment regimen**, n (%) | |  | |  |
| Irinotecan + oxaliplatin | | 16 (61.5%) | | 11 (33.3%) |
| Liposomal irinotecan + oxaliplatin | | 3 (11.5%) | | 8 (24.2%) |
| Liposomal irinotecan ± 5-FU | | 2 (7.7%) | | 5 (15.2%) |
| Platinum + fluoropyrimidines | | 1 (3.8%) | | 5 (15.2%) |
| Nab-paclitaxel based therapy | | 1 (3.8%) | | 2 (6.1%) |
| Gemcitabine based therapy | | 3 (11.5%) | | 1 (3.0%) |
| S-1 | | 0 | | 1 (3.0%) |
| **Efficacy** | |  | |  |
| Partial response, n (%) | | 1 (3.8%) | | 4 (12.1%) |
| Stable disease, n (%) | | 17 (65.4%) | | 14 (42.4%) |
| Progressive disease, n (%) | | 3 (11.5%) | | 5 (15.2%) |
| Not available, n (%) | | 5 (19.2%) | | 10 (30.3%) |
| Objective response rate (%) | | 3.8% | | 12.1% |
| Disease control rate (%) | | 69.2% | | 54.5% |
| Median treatment cycles (range) | | 4 (1-13) | | 4 (1-14) |
| Median of progression-free survival (95% CI), months | | 4.1 (2.4-5.8) | | 4.1 (3.4-4.8) |
| Median treatment duration (range), months | | 2.1 (0.5-7.6) | | 2.0 (0.5-10.7) |

Table S3. Progression-free survival (PFS) stratified by CA19-9 levels categorized by any decrease.

| Change in CA19-9 from baseline |  | NASCA (N=45) |  |  | Nab-paclitaxel and gemcitabine  (N=45) | | | |
| --- | --- | --- | --- | --- | --- | --- | --- | --- |
|  | Patients with any decrease | Patients without decrease | P | HR  (95% CI) | Patients with any decrease | Patients without decrease | P | HR  (95% CI) |
| **Any decrease**  **at week 6** | 31 | 14 |  |  | 34 | 11 |  |  |
| Median PFS  (95% CI), months | 9.3  (7.4-10.0) | 3.5  (1.7-7.0) | 0.002 | 0.33 (0.16-0.67) | 6.4  (4.9-7.2) | 2.6  (1.5-3.4) | <0.001 | 0.15 (0.07-0.35) |
| **Any decrease at week 12** | 30 | 6 |  |  | 30 | 8 |  |  |
| Median PFS  (95% CI), months | 9.3  (7.4-10.0) | 4.2  (2.8-NA) | 0.033 | 0.34 (0.12-0.92) | 6.4  (5.2-7.6) | 3.4  (2.6-5.1) | 0.001 | 0.23 (0.10-0.56) |

NA, not available.**Table S4. Antibodies used in the study.**

|  | Target | Source | Dilution | Opal fluorophores | Color |
| --- | --- | --- | --- | --- | --- |
| Panel 1 | CD163 | Abcam, ab182422 | 1:500 | Opal 620 | Red |
|  | CD8 | Abcam, ab178089 | 1:200 | Opal 690 | Magenta |
|  | CD68 | Abcam, ab213363 | 1:1000 | Opal 480 | Cyan |
|  | PD-1 | CST, D4W2J, 86163S | 1:200 | Opal 520 | Green |
|  | PD-L1 | CST, E1L3N, 13684S | 1:400 | Opal 570 | Yellow |
| Panel 2 | CD20 | DAKO, L26, IR604 | 1:1 | Opal 620 | Green |
|  | CD3 | DAKO, A0452 | 1:1 | Opal 690 | Magenta |
|  | CD56 | Abcam, ab75813 | 1:1000 | Opal 480 | Cyan |
|  | CD4 | Abcam, ab133616 | 1:100 | Opal 520 | Red |
|  | FOXP3 | Abcam, ab20034 | 1:100 | Opal 570 | Yellow |
| Detection in common | pan-CK | Abcam, ab7753 | 1:100 | Opal 780 | White |
