# Supplementary material for: First-line treatment with chemotherapy, surufatinib (an angio-immuno kinase inhibitor), and camrelizumab (an anti-PD-1 antibody) for locally advanced or metastatic pancreatic ductal adenocarcinoma: a phase Ib/II randomized study
Source: Signal Transduct Target Ther. 2025 Oct 13;10:339. doi: 10.1038/s41392-025-02441-2 (PMC12515981; doi:10.1038/s41392-025-02441-2)
Supplement: Supplementary file 3 — Supplementary information [file 41392_2025_2441_MOESM3_ESM.docx]

A Randomized, Controlled Phase Ib/II Study of Surufatinib Combined with Camrelizumab and the AS Regimen as First-Line Treatment for Advanced Pancreatic Carcinoma Metastatic

| Protocol No.: | HMPL-012-SPRING-P101 |
| --- | --- |
| Study Phase: | Phase Ib/II |
| Clinical Trial Institution: | Chinese PLA General Hospital |
| Principal Investigator: | Guanghai Dai / Quanli Han |
| Version No.: | 3.0 |
| Version Date: | March 7, 2022 |

**Protocol Signature Page of Sponsor**

We have read and confirmed this clinical trial protocol (Protocol No.: HMPL-012-SPRING-P101, Version: 3.0, Version Date: March 7, 2022). We agree to perform relevant duties in accordance with Chinese law, the Declaration of Helsinki, ICH-GCP, and this study protocol, and to implement only after obtaining approval from the Ethics Committee (EC), unless measures must be taken to protect the safety, rights, and interests of the subjects.

**Sponsor:** Chinese PLA General Hospital

| Guanghai Dai |  |  |
| --- | --- | --- |
| Principal Investigator (Print) | Principal Investigator (Signature) | Date of Signature (MM/DD/YYYY) |

| Quanli Han |  |  |
| --- | --- | --- |
| Principal Investigator (Print) | Principal Investigator (Signature) | Date of Signature (MM/DD/YYYY) |

**Protocol Signature Page of Principal Investigator**

I will diligently perform the duties of an investigator in accordance with Chinese GCP regulations, personally participate in or directly supervise this clinical study. I have received the Investigator's Brochure for the investigational product of this clinical trial; I am aware of and have read the preclinical study information of the investigational product and the protocol of this clinical trial. I agree to perform relevant duties in accordance with Chinese law, the Declaration of Helsinki, Chinese GCP, and this study protocol. I will be responsible for making clinically relevant medical decisions, ensuring that subjects receive timely and appropriate treatment in the event of adverse events (AEs) during the study, and recording and reporting these AEs in accordance with national regulations. I assure that the data will be recorded truthfully, accurately, completely, and timely. I will accept institutional supervision and inspection throughout the clinical study, conduct regular self-inspections of the clinical study I am responsible for, report relevant matters timely and truthfully, and ensure the quality of the clinical trial. I commit to keeping the personal information of subjects and related matters confidential. I agree to disclose my full name and profession to the sponsor, agree to disclose expenses related to the clinical study upon request, and agree to prohibit commercial or economic activities related to this trial. I agree that the study results may be published publicly.

**Study Institution:** Chinese PLA General Hospital

| Guanghai Dai |  |  |
| --- | --- | --- |
| Principal Investigator (Print) | Principal Investigator (Signature) | Date of Signature (MM/DD/YYYY) |

| Quanli Han |  |  |
| --- | --- | --- |
| Principal Investigator (Print) | Principal Investigator (Signature) | Date of Signature (MM/DD/YYYY) |

Table of Contents

[Table of Contents 1](#_Toc184889595)

[**Table 1 Study Flowchart** 11](#_Toc184889596)

[1. Introduction: Study Background and Scientific Rationale 18](#_Toc184889597)

[1.1. Study Background 18](#_Toc184889598)

[1.2. Physicochemical Properties and Pharmaceutical Characteristics of the Investigational Product 22](#_Toc184889599)

[1.3. Preclinical Study Summary 23](#_Toc184889600)

[1.3.1. Preclinical Pharmacodynamic Studies 23](#_Toc184889601)

[1.3.2. Preclinical Toxicology Studies 24](#_Toc184889602)

[1.4. Study Rationale 27](#_Toc184889603)

[2. Study Endpoints 29](#_Toc184889604)

[2.1. Primary Study Endpoints 29](#_Toc184889605)

[2.2. Secondary Study Endpoints 30](#_Toc184889606)

[3. Study Design 30](#_Toc184889607)

[3.1. Overall Design 30](#_Toc184889608)

[4. Subject Selection and Withdrawal 31](#_Toc184889609)

[4.1. Inclusion Criteria 31](#_Toc184889610)

[4.2. Exclusion Criteria 32](#_Toc184889611)

[4.3. Subject Completes Study Treatment, Withdraws from Study, or Discontinues Study Treatment 35](#_Toc184889612)

[4.3.1. Criteria for Completion of Study Treatment: 35](#_Toc184889613)

[4.3.2. Withdrawal from the Study 36](#_Toc184889614)

[4.3.3. Discontinuation Criteria 36](#_Toc184889615)

[4.3.4. Procedures for Withdrawal from the Study or Discontinuation of Study Treatment 37](#_Toc184889616)

[4.4. Dropout Criteria 37](#_Toc184889617)

[4.5. Restrictions during the Study 38](#_Toc184889618)

[4.6. Early Discontinuation or Suspension of the Study 38](#_Toc184889619)

[5. Treatment Regimen 39](#_Toc184889620)

[5.1. Name and Source of Investigational Product 39](#_Toc184889621)

[5.1.1. Drug Source 39](#_Toc184889622)

[5.1.2. Drug Dosage Form, Strength and Storage Conditions 39](#_Toc184889623)

[5.1.3. Investigational Product Randomization/Supply Scheme 39](#_Toc184889624)

[5.1.4. Drug Management 40](#_Toc184889625)

[5.2. Method of Administration 40](#_Toc184889626)

[5.2.1. Dosing Regimen and Dosing Period 40](#_Toc184889627)

[5.2.2. Method of Administration 41](#_Toc184889628)

[5.3. Precautions 41](#_Toc184889629)

[5.4. Dose Adjustment 46](#_Toc184889630)

[5.4.1. Principles of Dose Adjustment 46](#_Toc184889631)

[5.4.2. Detailed Procedures for Dose Adjustment 46](#_Toc184889632)

[5.5. Concomitant and Excluded Medications 51](#_Toc184889633)

[5.5.1. Concomitant Medications 51](#_Toc184889634)

[5.5.2. Excluded Medications 52](#_Toc184889635)

[5.5.3. Drug Interaction 52](#_Toc184889636)

[5.6. Subject Compliance 53](#_Toc184889637)

[5.7. Study Procedures 53](#_Toc184889638)

[5.8. Screening 53](#_Toc184889639)

[5.9. Enrollment 54](#_Toc184889640)

[5.10. Treatment Period 54](#_Toc184889641)

[5.11. Follow-up Period 55](#_Toc184889642)

[5.12. Continuation of Medication after the End of the Study 55](#_Toc184889643)

[6. Evaluation 56](#_Toc184889644)

[6.1. Efficacy Evaluation 56](#_Toc184889645)

[6.2. Safety Evaluation 57](#_Toc184889646)

[6.3. European Organisation for Research and Treatment of Cancer (EORTC) Quality of Life Questionnaire 59](#_Toc184889647)

[7. AE Report 60](#_Toc184889648)

[7.1. AE 60](#_Toc184889649)

[7.1.1. Definition of AE 60](#_Toc184889650)

[7.1.2. Criteria for AE Severity 60](#_Toc184889651)

[7.1.3. Determination of Causality between AE and Investigational Product 61](#_Toc184889652)

[7.2. SAE 61](#_Toc184889653)

[7.2.1. Definition of SAE 61](#_Toc184889654)

[7.2.2. Hospitalisation 62](#_Toc184889655)

[7.2.3. Disease Progression and Death 63](#_Toc184889656)

[7.2.4. Other Anti-tumor Therapies 64](#_Toc184889657)

[7.2.5. SAE Reporting System 64](#_Toc184889658)

[7.3. Pregnancy 65](#_Toc184889659)

[7.4. Follow-up for AEs/SAEs 66](#_Toc184889660)

[8. Statistical Methods and Analysis 67](#_Toc184889661)

[8.1. Analysis Population 67](#_Toc184889662)

[8.2. Efficacy Analysis 67](#_Toc184889663)

[8.2.1. Primary Efficacy Analysis 67](#_Toc184889664)

[8.2.2. Secondary Efficacy Analysis 67](#_Toc184889665)

[8.2.3. Quality of Life Scores and Tumor Biomarkers 68](#_Toc184889666)

[8.3. Safety Analysis 68](#_Toc184889667)

[8.4. Sample Size Determination 69](#_Toc184889668)

[9. Data Management Methods 69](#_Toc184889669)

[9.1. Data Recording 69](#_Toc184889670)

[9.1.1. Completion of Original Medical Records 69](#_Toc184889671)

[9.1.2. eCRF Completion 69](#_Toc184889672)

[9.1.3. eCRF Review 70](#_Toc184889673)

[9.2. Data Management 70](#_Toc184889674)

[9.2.1. Establishment of EDC Database 70](#_Toc184889675)

[9.2.2. Data Review and Database Lock 70](#_Toc184889676)

[9.2.3. Data Archiving 70](#_Toc184889677)

[10. Source Data and Source Documents 71](#_Toc184889678)

[11. Quality Assurance and Quality Control 71](#_Toc184889679)

[12. Ethics 72](#_Toc184889680)

[12.1. Ethical Codes 72](#_Toc184889681)

[12.2. Independent EC 73](#_Toc184889682)

[12.3. Informed Consent 73](#_Toc184889683)

[12.3.1. Informed Consent Form and Other Written Information Required by Subjects 73](#_Toc184889684)

[12.3.2. Informed Consent Process and Records 73](#_Toc184889685)

[12.4. Confidentiality of Subject Information 74](#_Toc184889686)

[12.4.1. Study Use of Samples, Specimens, or Data 74](#_Toc184889687)

[13. Publication of Study Results 74](#_Toc184889688)

[13.1. Utilization of Study Data 74](#_Toc184889689)

[13.2. Publication 75](#_Toc184889690)

[14. References 75](#_Toc184889691)

[15. Appendix 78](#_Toc184889692)

[**Appendix 1: Guideline on Potential Interactions of Surufatinib with Concomitant Medications** 78](#_Toc184889693)

[**Appendix 2: Traditional Chinese Medicines Prohibited During the Study** 81](#_Toc184889694)

**Protocol Summary**

| **Study Title** | A Randomized, Controlled Phase Ib/II Study of Surufatinib Combined with Camrelizumab and the AS Regimen as First-Line Treatment for Advanced Pancreatic Carcinoma Metastatic |
| --- | --- |
| **Protocol No.** | HMPL-012-SPRING-P101 |
| **Version No.** | 3.0 |
| **Version Date** | March 7, 2022 |
| **Investigational Product** | Surufatinib, camrelizumab, tegafur, nab-paclitaxel |
| **Sponsor** | Chinese PLA General Hospital |
| **Principal Investigator** | Guanghai Dai / Quanli Han |
| **Study Endpoints** | **Primary Study Endpoints**   - Phase Ib: dose-limiting toxicities (DLTs), recommended Phase II dose (RP2D) of surufatinib; - Phase II: objective response rate (ORR)   **Secondary Study Endpoints**   - Progression-free survival (PFS) - Disease control rate (DCR) - Overall survival (OS) - Duration of response (DOR) - Time to response (TTR) - Safety |
| **Study Population** | This study enrolls patients with unresectable locally advanced or pancreatic carcinoma metastatic who have not previously received systemic therapy. |
| **Study Design** | This is a single-center, randomized, controlled, Phase Ib/II study. |
| **Method of Administration** | - Phase Ib dose-exploration stage (9-15 patients):  This study enrolls patients with advanced pancreatic carcinoma metastatic who have not previously received systemic therapy for advanced disease; in Phase Ib, 9 to 15 subjects will be recruited to explore the DLTs of surufatinib combined with camrelizumab and the AS regimen, and to determine the recommended Phase II dose (RP2D) of surufatinib;  Medication regimen: a 3+3 dose escalation scheme is adopted. Surufatinib will undergo dose escalation from low to high according to the following doses: L1: 200 mg/d, L2: 250 mg/d, L3: 300 mg/d, administered continuously as QD po; combined with camrelizumab: 200 mg, intravenous (IV) infusion, q3w, and the 3-week regimen of nab-paclitaxel combined with tegafur: nab-paclitaxel: 125 mg/m^2^, IV, d1, d8, q3w; tegafur: 40 mg, BID po, d1-14, q3w. Three weeks of combination therapy constitute one treatment cycle. DLTs will be recorded during the first cycle of administration.  Phase II plans to enroll 90 patients, randomly assigned 1:1 into the test group (45 cases) or the control group (45 cases), to receive the following treatment regimens:  Test group:  In combination with camrelizumab, nab-paclitaxel, and tegafur (administration regimen is the same as the dose in the Phase Ib exploration stage) based on the RP2D of surufatinib determined in Phase Ib, until intolerable toxicity or disease progression, death, or reaching other criteria specified in the protocol for discontinuation of study treatment.  Control group: GN 3-week regimen (nab-paclitaxel: 125 mg/m^2^, IV, d1, d8, q3w; gemcitabine: 1000 mg/m^2^, IV infusion over 30 minutes, d1, d8, q3w), until intolerable toxicity or disease progression, death, or reaching other criteria specified in the protocol for discontinuation of study treatment.  This study is divided into three phases: screening phase, treatment phase, and follow-up phase. Every 6 weeks (±2 days), imaging methods will be used to assess the tumor status until disease progression (RECIST 1.1), death (during the patient’s treatment), or intolerable toxicity occurs, and record the tumor treatment and survival status after disease progression.  Safety indicators include: AE, laboratory test, vital signs, and changes in electrocardiogram and cardiac ultrasound. |
| **Inclusion Criteria** | Patients must meet all the following criteria to be enrolled in this study:   1. Have fully understood this study and voluntarily signed the informed consent form; 2. Patients with histologically or cytologically confirmed unresectable, locally advanced, or metastatic pancreatic ductal adenocarcinoma; 3. Age 18-75 years old (inclusive); 4. No prior systemic therapy for advanced pancreatic carcinoma; 5. ECOG PS 0-1; 6. Must have at least one measurable lesion (according to Response Evaluation Criteria in Solid Tumors, version 1.1 [RECIST 1.1]); 7. Expected survival ≥ 3 months; 8. The functions of vital organs meet the following requirements (the use of any blood components and cell growth factors within *14 days before enrollment is not allowed):   Absolute neutrophil count (ANC) ≥1.5×10^9^/L;  Platelet count ≥100×10^9^/L;  Haemoglobin ≥90 g/L;  Total bilirubin < 1.5 × ULN;  ALT and/or AST < 1.5 × ULN ( < 3 × ULN for patients with metastases to liver);  Serum creatinine < 1.5 × ULN;  Endogenous creatinine clearance ≥50 mL/min;   1. Women of childbearing potential must use effective contraceptive measures; 2. Good compliance and cooperative with follow-up. |
| **Exclusion Criteria** | Patients who meet any of the following criteria will not be enrolled in this study:   1. Unable to comply with the study protocol or study procedures; 2. Previously received treatment with vascular endothelial growth factor receptor (VEGFR) inhibitors, or previously used immune checkpoint inhibitor (ICI) treatment; 3. Patients carrying germline *BRCA1/2* mutations; 4. Participating in or having participated in other drug clinical trials within 4 weeks prior to enrollment; 5. Have received transfusion therapy, blood products, and hematopoietic factors, such as albumin and granulocyte colony-stimulating factor (G-CSF), within 14 days prior to enrollment; 6. Brachytherapy (radioactive seed implantation) within 60 days prior to enrollment; 7. Have received other systemic anti-tumor treatments within 4 weeks prior to enrollment, including chemotherapy, signal transduction inhibitors, hormone therapy, and immunotherapy; 8. Have received any surgery or invasive treatment or procedure within 4 weeks prior to enrollment (excluding intravenous catheterization, paracentesis drainage, etc.); 9. Undergone major surgery within 60 days prior to enrollment or the surgical incision has not completely healed; 10. Have received local anti-tumor treatments within 4 weeks prior to enrollment, such as hepatic arterial interventional embolism, cryoablation or radiofrequency ablation of metastases to liver; 11. The patient currently has hypertension uncontrolled by medication, defined as: blood pressure systolic ≥140 mmHg and/or blood pressure diastolic ≥90 mmHg; 12. Protein urine ≥2+, or 24-hour protein urine amount ≥1.0 g on urinalysis; 13. Uncontrollable malignant ascites (defined as ascites that cannot be controlled by diuretics or paracentesis as judged by the investigator); 14. Clinically significant electrolyte abnormalities as judged by the investigator; 15. Liver metastases accounted for half or more of the total liver volume as determined by the investigator; 16. Clinically significant cardiovascular disorders, including but not limited to acute myocardial infarction within 6 months prior to enrollment, severe/unstable angina pectoris, or coronary artery bypass surgery; cardiac failure congestive with New York Heart Association (NYHA) classification > Class II; ventricular arrhythmia requiring drug therapy; LVEF (left ventricular ejection fraction) <50%; 17. Hemorrhage events of ≥ Grade 3 occurring within 4 weeks prior to enrollment; 18. Patients who, within 3 months prior to enrollment, have clear evidence or history of haemorrhagic tendency (hemorrhage >30 mL within 3 months, occurrence of haematemesis, melena, haematochezia), haemoptysis (fresh blood >5 mL within 4 weeks), or have experienced thromboembolic events within 12 months (including stroke events and/or transient ischaemic attack); 19. International Normalized Ratio (INR) > 1.5 or activated partial thromboplastin time (APTT) > 1.5×ULN, or the patient is currently taking anticoagulants; 20. Currently, the patient has poorly controlled diabetes mellitus (after standard treatment, fasting glucose concentration ≥ CTCAE Grade 2); 21. The patient currently has any disease or condition that affects drug absorption, or the patient is unable to take surufatinib orally; 22. Active or uncontrolled severe infection (≥CTCAE Grade 2 infection); 23. Known human immunodeficiency virus (HIV) infection; 24. Known history of clinically significant liver disease, including hepatitis viral [subjects known to be carriers of hepatitis b virus (HBV) must be excluded if they have active HBV infection, i.e., HBV DNA positive (>1×10^4^ copies/mL or >2000 IU/mL); known hepatitis c virus (HCV) infection with HCV RNA positive (>1×10^3^ copies/mL)], or other hepatitis, hepatic cirrhosis; 25. The patient currently has central nervous system (CNS) metastasis or a history of brain metastasis; 26. Patients who currently have gastrointestinal diseases such as active gastric and duodenal ulcer, colitis ulcerative, or active hemorrhage in unresected tumor, or other conditions that may cause gastrointestinal hemorrhage or perforation as determined by the investigator; 27. Unresolved toxicities higher than CTCAE Grade 1 caused by any prior anti-cancer treatments, excluding alopecia and ≤ Grade 2 neurotoxicity caused by oxaliplatin; 28. Patients with a known or suspected allergy to the investigational product or drugs of the same class; 29. Pregnant (positive pregnancy test before medication) or breastfeeding women; 30. Drug abuse, medical, psychological, or social conditions may affect patient enrollment and the evaluation of experimental results; 31. Having other untreated or concomitant tumors, except cervical carcinoma in situ, treated basal cell carcinoma, or superficial bladder tumors. Patients can be enrolled if the tumor has been radically resected and there is no evidence of disease for more than 3 years. Treatment for all other tumors must have been completed at least 3 years prior to enrollment; 32. Patients considered by the investigator to be inappropriate for enrollment in this study. |
| **Criteria for Subjects to Discontinue the Study Treatment** | **Discontinuation of Investigational Product:**  **Criteria for completion of study treatment** (any of the following criteria is met):   1. Disease progression (including clinical progression); 2. Subject death; 3. The entire study is completed.   Criteria for early discontinuation of study treatment (any of the following criteria is met):   1. Unable to continue study treatment due to toxicity of the investigational product; 2. The subject or their legally authorized representative withdraws informed consent and requests withdrawal from the study; 3. The investigator believes that the study treatment should be discontinued in the best benefit of the subject; 4. Pregnancy during the study; 5. Poor subject compliance, unable to adhere to the study protocol for medication and follow-up; 6. Simultaneously received other anti-tumor drugs during the study, including chemotherapy, targeted therapy, and immunotherapy; 7. Loss to follow-up. |
| **Study Discontinuation Criteria** | 1. The study will be completed when study objectives are archived, or; 2. The number and severity of AEs in this study suggest that it is not appropriate to continue the clinical study. |
| **Dropout Criteria** | All subjects who have signed the informed consent form and passed screening to enter the study have the right to withdraw from this study at any time.   1. For subjects who have only signed the informed consent form and passed screening but have not been enrolled, they will not be considered as dropouts. 2. After enrollment, any subject who does not complete two cycles of the trial and therefore cannot be evaluated for efficacy will be considered as a dropout. Except for the following reasons:    1. After enrollment, subjects who experience disease progression with clear medical evidence are not considered as dropouts; however, radiological evidence must be provided;    2. Subjects who discontinue participation due to pregnancy or intolerable toxicity should not be considered as dropouts;    3. The investigator determines that treatment should be discontinued in the best benefit of the subject;    4. Death after enrollment.   For dropout subjects, the investigator must record the reason for dropout, complete the relevant tumor assessments as much as possible, and record the circumstances of the last visit. |
| **Sample Size Determination** | According to literature results, the rate in the test group is 0.49, and the rate in the control group is 0.23. With a superiority margin of 0, a one-sided alpha of 0.05, beta of 0.2, and a sample size ratio of 1 between the two groups (test group: control group), the calculated sample sizes are 40 cases in the test group and 40 cases in the control group, totaling 80 cases. Considering a 10% dropout rate, the total required sample size is 90 cases. |
| **Data Analysis and Statistical Methods** | Progression-Free Survival (PFS) is defined as the time from randomization to disease progression or death. For patients who have not reported death at the time of analysis, the date of their last known survival will be taken as the censoring date. The comparison of Objective Response Rate (ORR) between treatment groups will be based on the Intent-to-Treat (ITT) population. The ITT population is defined as all randomized subjects.  The Kaplan-Meier method will be used to estimate the median survival time of each treatment group, and Kaplan-Meier curves will be plotted to provide a visually intuitive description of the differences between treatment groups. The evaluation of efficacy will be displayed through hazard ratios (HR) estimated by the Cox regression model and their 95% confidence intervals. At the same time, the log-rank test will be used to compare parameters such as PFS and OS between the two groups. |
| **Study Progress** | Estimated enrollment of the first subject: August 2021  Estimated enrollment of the last subject: August 2024  Estimated end of study: August 2025 (including follow-up, data cleaning, and data summary report writing) |

**Table 1 Study Flowchart**

| **Study Period** | **Screening^[1]^** | | **Treatment Period** | | | | | **Follow-up Period** | |
| --- | --- | --- | --- | --- | --- | --- | --- | --- | --- |
|  |  |  | **Cycle 1** | | **Cycle 2** | **Subsequent Cycles** | **End of Treatment/Withdrawal from Study** | **Safety Follow-up**^[17]^ | **Survival Follow-up** |
|  | **Screening 1** | **Screening 2** | **D1** | **D21** | **D21** | **D21** | **EOT** | **Within 30 days after EOT** | **Every three months for one year after EOT, and every 6 months thereafter** |
| **Window Period** | **Within 21 days prior to dosing** | **Within 7 days prior to dosing** | **Day 0** | **±2 days** | **±2 days** | **±2 days** | **±3 days** | **±7 days** | **±7 days** |
| Signing informed consent^[1]^ | X |  |  |  |  |  |  |  |  |
| Demographic data | X |  |  |  |  |  |  |  |  |
| Medical history^[2]^ | X |  |  |  |  |  |  |  |  |
| BRCA1/2 gene testing | X |  |  |  |  |  |  |  |  |
| ECOG score |  | X |  | X | X | X | X | X |  |
| Physical examination^[3]^ |  | X |  | X | X | X | X | X |  |
| EORTC QLQ-C30 quality of life questionnaire |  | X | X, it is to be performed at the Day 1 visit of each treatment cycle from Cycle 2 onwards until the end of treatment. It should be completed before various clinical examinations and clinical assessments are performed and before being informed of any new disease information and initiation of any new treatment. | | | | |  |  |
| Vital signs |  | X |  | X | X | X | X | X |  |
| Blood pressure (recorded in the subject's diary card) |  | X |  | X | X | X | X | X |  |
| **Study Period** | **Screening^[1]^** | | **Treatment Period** | | | | | **Follow-up Period** | |
|  |  |  | **Cycle 1** | | **Cycle 2** | **Subsequent Cycles** | **End of Treatment/Withdrawal from Study** | **Safety Follow-up**^[17]^ | **Survival Follow-up** |
|  | **Screening 1** | **Screening 2** | **D1** | **D21** | **D21** | **D21** | **EOT** | **Within 30 days after EOT** | **Every three months for one year after EOT, and every 6 months thereafter** |
| **Window Period** | **Within 21 days prior to dosing** | **Within 7 days prior to dosing** | **Day 0** | **±2 days** | **±2 days** | **±2 days** | **±3 days** | **±7 days** | **±7 days** |
| Stool routine + fecal occult blood |  | X |  | X | X | X | X | X |  |
| Immunology test |  | X |  | X | X | X | X | X |  |
| Hepatitis B and C testing^[4]^ | X |  |  |  |  |  |  |  |  |
| HIV testing^[5]^ | X |  |  |  |  |  |  |  |  |
| Blood pregnancy^[6]^ |  | X |  |  |  |  |  | X |  |
| Hematology^[7]^ |  | X |  | X | X | X | X | X |  |
| Blood chemistry^[8]^ |  | X |  | X | X | X | X | X |  |
| Coagulation^[9]^ |  | X |  | X | X | X | X | X |  |
| Thyroid function^[10]^ |  | X |  | X | X | X | X | X |  |
| Urinalysis^[11]^ |  | X |  | X | X | X | X | X |  |
| Echocardiogram |  | X | X, left ventricular ejection fraction (LVEF) will be evaluated every 6 weeks ± 2 days from start of treatment. | | | | | X |  |
| 12-lead electrocardiogram^[12]^ |  | X |  | X | X | X | X | X |  |
| Tumor imaging^[13]^ |  | X | X, tumor imaging evaluation (CT or MRI) will be performed every 6 weeks ± 2 days after the start of treatment until disease progression, death, or withdrawal of consent, whichever occurs first. If there is early withdrawal or withdrawal due to non-disease progression factors, it is recommended to continue evaluation until disease progression occurs. Unscheduled tumor assessments may be performed by the investigator as clinically indicated. | | | | |  |  |
| **Study Period** | **Screening^[1]^** | | **Treatment Period** | | | | | **Follow-up Period** | |
|  |  |  | **Cycle 1** | | **Cycle 2** | **Subsequent Cycles** | **End of Treatment/Withdrawal from Study** | **Safety Follow-up**^[17]^ | **Survival Follow-up** |
|  | **Screening 1** | **Screening 2** | **D1** | **D21** | **D21** | **D21** | **EOT** | **Within 30 days after EOT** | **Every three months for one year after EOT, and every 6 months thereafter** |
| **Window Period** | **Within 21 days prior to dosing** | **Within 7 days prior to dosing** | **Day 0** | **±2 days** | **±2 days** | **±2 days** | **±3 days** | **±7 days** | **±7 days** |
| Tumor marker testing |  | X | X (at each tumor assessment visit) | | | | | X |  |
| Tumor specimen collection |  | X |  | | | | |  |  |
| Drug dispensing/recovery^[14]^ |  | X |  | X | X | X | X |  |  |
| Concomitant medication/treatment^[15]^ | X |  | X | X | X | X | X | X |  |
| Record AE^[16]^ | X |  | X | X | X | X | X | X |  |
| Survival follow-up^[18]^ |  |  |  |  |  |  |  | X | X |

Note: in addition to the test items and time points listed in the table, the investigator may add items that are necessary at any time, and fill out in the CRF’s Additional Examinations at Visits.

1. Informed consent must be obtained before all study-specific procedures in this study, which can be signed before the 21-day window period of screening. The subject who fails screening may be re-screened once, and need to re-sign the ICF and obtain a new subject number.
2. The time of the last dose of prior anti-tumor therapy must be recorded.
3. Physical examination: physical examination included height (at screening only), weight, head, eyes, ears, nose, throat, neck, heart, chest (including lungs), abdomen, extremities, skin, lymph nodes, neurological status, and general condition. Once at screening and every 3 weeks (± 2 days) during dosing.
4. All subjects will undergo HCV antibody, hepatitis B five items (HBsAg, HBsAb, HBcAb, HBeAg and HBeAb) and HBV-DNA tests at screening at each local laboratory. HCV RNA test will be performed in HCV antibody positive subjects.
5. HIV antibody screening is performed at screening, and tests performed prior to screening are also acceptable and do not need to be repeated at screening. If HIV antibody screening prior to or during the screening is positive, enrollment is not allowed.
6. Blood pregnancy tests will be performed at screening and at the 30-day safety follow-up visit. Female subjects of childbearing potential should complete the test within 7 days prior to the first dose of the investigational product. Women of childbearing potential must undergo a serum pregnancy test, and enrolled subjects must have a negative serum test result. Female subjects are still considered to have childbearing potential if they are postmenopausal but have not reached postmenopausal status (menopausal for a period of ≥ 12 consecutive months, for no other reason other than menopause) and have not undergone sterilization (removal of ovaries and/or uterus), and should have the serum pregnancy test.
7. Hematology: complete blood count (absolute), including red blood cell count, white blood cell count with differential (neutrophils, lymphocytes, monocytes, eosinophils and basophils), platelet count, hemoglobin, and hematocrit, should be performed within 7 days prior to the first dose of the investigational product. It should be performed within 2 days prior to dosing on Day 1 of each cycle from Cycle 2 onwards and during the safety follow-up period. Unscheduled tests may be performed as clinically indicated.
8. Clinical chemistry includes ALT, AST, total bilirubin, BUN (urea nitrogen) or urea, Cr (blood creatinine), blood glucose, potassium, sodium, chloride, calcium, phosphorus, magnesium, total protein, albumin, albumin/globulin ratio, LDH (lactate dehydrogenase), ALP (alkaline phosphatase), AFP (α-fetoprotein), amylase, lipase, triglycerides and total cholesterol, and should be performed within 7 days prior to the first dose of the investigational product. It should be performed within 2 days prior to dosing on Day 1 of each cycle from Cycle 2 onwards and during the safety follow-up period. Unscheduled tests may be performed as clinically indicated.
9. Coagulation function includes prothrombin time (PT), activated partial thrombin time (APTT) and INR. It should be performed within 7 days prior to the first dose of the investigational product. It should be performed within 2 days prior to dosing on Day 1 of each cycle from Cycle 2 onwards and during the safety follow-up period. Unscheduled tests may be performed as clinically indicated.
10. Thyroid function: including serum free triiodothyronine (FT3), serum free thyroxine (FT4), and thyroid stimulating hormone (TSH), to be performed within 7 days prior to the first dose of the investigational product. It should be performed within 2 days prior to dosing on Day 1 of each cycle from Cycle 2 onwards and during the safety follow-up period. Unscheduled tests may be performed as clinically indicated.
11. Urinalysis: including specific gravity, pH, white blood cells, red blood cells, protein, glucose, ketone bodies and casts, to be performed within 7 days prior to the first dose. It is recommended to perform 24-hour urine protein quantification as soon as possible (e.g., within 72 h) if it shows urinary protein++ in two consecutive urinalyses.
12. Cardiac monitoring: left ventricular ejection fraction (LVEF) will be evaluated by echocardiography at screening, every 6 weeks ± 2 days after the start of treatment, and within 30 days after the end of treatment; 12-lead electrocardiogram will be performed at screening, within 2 days prior to dosing on Day 1 of each treatment cycle starting from Cycle 2, and within 30 days after the end of treatment.
13. Baseline tumor assessment will be performed within 21 days before the first dose of the investigational product. Baseline tumor evaluation will be performed according to RECIST v1.1. Imaging (CT or MRI) examinations of the chest and abdomen may be performed, and if clinically indicated, any other known or suspected sites of disease (e.g., pelvis) may be performed using appropriate methods (CT scan or MRI). The presence or absence of a primary lesion, the presence or absence of measurable disease/or the non-measurable disease will be recorded. Baseline and post-treatment response assessments will be performed using the same method and by the same investigator if possible (RECIST 1.1 criteria); tumor imaging assessments (CT or MRI), will be performed every 6 weeks (+/-2 days), after the start of treatment until disease progression, death, or withdrawal of consent, whichever occurs first. Unscheduled tumor assessments may be performed by the investigator as clinically indicated. At the same time, BIRC will be established for real-time acquisition and blinded independent central assessment of tumor imaging data (RECIST v1.1 criteria).
14. Drug dispensing/recovery: surufatinib is taken orally once daily, 21 days per cycle, dispensed once per cycle. Unused study medication should be returned to the study site at the end of each treatment cycle or at the beginning of the next treatment cycle, and a safety visit must be completed before dispensing the product.
15. Concomitant medications include any prescription and over-the-counter drugs. All medications taken by the patient between 21 days prior to the first dose and the 30-day safety visit should be recorded, including: generic name and daily dose of the product; reason for use of the product; and the start and end dates of use. Only subsequent anti-tumor therapies will be recorded after the 30-day safety visit.
16. AEs will be collected from the time of signing the informed consent form until 30 days after the last dose of the investigational product. Serious adverse events (SAEs) will be collected from the time of signing the informed consent form until 30 days after the last dose or the start of new anti-tumor therapy, and only SAEs related to the investigational product will be collected 30 days after the last dose. AEs related to investigational product will be followed until they recover to Grade 0 - 1 or to baseline, or assessed by the investigator as reaching a stable state, or confirmed unrelated to study treatment, or until the subject starts a new anti-tumor therapy, is lost to follow-up, or withdraws consent.
17. Subjects who complete or early discontinue the investigational product treatment should return to the study site for safety assessment 30 (± 7) days after the last dose or before the initiation of other anti-tumor therapy, whichever occurs first. SAEs occurring within 30 days after the last treatment should be followed up to 30 (+/-7) days after the last treatment if possible or until the initiation of another anti-tumor therapy, whichever occurs first. Continuation of tumor imaging assessments at this visit is recommended for subjects who have completed treatment but have not been assessed as disease progression.
18. Survival follow-up: survival follow-up will be performed every 3 months for one year after the end of treatment, and every 6 months after one year. Subsequent anti-tumor therapy and study-related SAEs will be collected. All surviving subjects will be followed for survival once before the end of the entire study.

# Introduction: Study Background and Scientific Rationale

## Study Background

Pancreatic carcinoma, known as the king of cancers, is one of the malignant tumors of the digestive system with extremely high mortality. The global overall incidence and death rates are increasing year by year, resulting in 466,000 deaths worldwide in 2020^[1]^. In our country, the mortality rate of pancreatic carcinoma ranks sixth among malignant tumors. In 2020, there were 125,000 new cases and 122,000 deaths, characterized by high malignancy and poor prognosis.

The main treatment methods for pancreatic carcinoma include surgical resection, chemotherapy, radiotherapy, and biological therapy, but the therapeutic effects are not ideal; it is one of the malignant tumors with the poorest treatment outcomes. The 5-year survival rate for patients with pancreatic carcinoma is only 7.2%, the lowest among malignant tumors^[2]^; it is predicted that by 2030, the mortality of pancreatic carcinoma will be second only to lung cancer^[3]^. At present, surgery is still the only method by which pancreatic carcinoma patients can achieve long-term survival. However, more than 80% of advanced pancreatic carcinoma patients have lost the possibility of surgery, making aggressive medical treatment particularly important. The overall treatment efficacy for locally advanced pancreatic carcinoma or pancreatic carcinoma combined with distant metastasis is poor; it is recommended to carry out relevant clinical studies. For patients with advanced pancreatic carcinoma who have no progression after first-line chemotherapy and have good performance status, maintenance therapy can be considered. The choice of drugs and duration of therapy for maintenance treatment require further clinical exploration and verification.

At present, the treatment of pancreatic carcinoma metastatic still mainly relies on chemotherapy. For patients with pancreatic carcinoma metastatic who have lost the opportunity for surgery and have KPS≥70, standard treatment generally involves combination chemotherapy. Among them, there are currently two internationally recognized standard first-line chemotherapy regimens: the three-drug regimen of oxaliplatin + irinotecan + 5-FU/leucovorin (FOLFIRINOX), and the two-drug regimen of nab-paclitaxel + gemcitabine (AG). The expected response rate of the FOLFIRINOX regimen is 30%, with an expected survival of 11-12 months; the expected response rate of the AG regimen is 25%-30%, with an expected survival of 8.5-11 months^[4-7]^. The AG regimen and the FOLFIRINOX regimen can serve interchangeably as first-line and second-line treatments; that is, after patients develop drug resistance to the first-line treatment, they can switch to the other regimen for continued therapy. Retrospective studies of the two regimens indicate that the sequence of use does not affect patient prognosis^[8]^.

A randomized controlled phase II study presented at the 2020 ASCO GI further evaluated the efficacy and safety of nab-paclitaxel (nab-P) combined with tegafur (S-1) compared to nab-paclitaxel combined with gemcitabine (Gem) in the treatment of advanced ductal adenocarcinoma of pancreas. The results showed that compared to the nab-P/Gem group, the nab-P/S-1 group had higher ORR, primary lesion ORR, and longer PFS, without statistical significance. The nab-P/S-1 regimen may have lower hematological toxicity. The survival status of patients is currently under further follow-up. The results suggest that the efficacy of the AS regimen (nab-P/S-1) is similar to that of the AG regimen (nab-P/Gem) and may be considered as a substitute for the AG regimen in advanced chemotherapy^[9]^. A recent retrospective study published by our site on 322 patients with advanced pancreatic carcinoma from four hospitals in China showed that among 232 patients receiving the AS regimen, 79 patients receiving the AG regimen, and 11 patients receiving the FOLFIRINOX regimen, the ORRs were 46.9%, 18.7%, and 0%, respectively; the DCRs were 87.2%, 69.3%, and 63.6%, respectively; the incidence rates of Grade 3/4 AEs were 29.9%, 25%, and 36.4%, respectively. The ORR and DCR of the AS regimen were higher than those of the other two regimens, and the incidence of AEs was lower^[10]^.

The traditional treatment regimen for pancreatic carcinoma is gemcitabine-based chemotherapy, but in recent years, multiple studies have been conducted on targeted therapy and immunotherapy.

ICIs have become a novel therapeutic approach for certain tumors, but early study results of ipilimumab or PD-L1 monoclonal antibodies in the treatment of advanced pancreatic carcinoma are not ideal^[11]^. Pancreatic carcinoma usually has a low tumor mutational burden (TMB) (1 mt/Mb), while high TMB is usually associated with high response rates to immunotherapy. However, the combination therapy of ICIs with radiotherapy and/or chemotherapy has shown encouraging study results. Therefore, it is important to select an appropriate target population, rational immunotherapy combinations (e.g., combination radiotherapy, chemotherapy, targeted therapies, etc., and combinations of multiple immunotherapies).

An initial analysis of a phase Ib clinical study of ipilimumab combined with gemcitabine in the treatment of locally advanced/unresectable/pancreatic carcinoma metastatic included 16 patients, and the results showed that the median progression-free survival (PFS) of the combination therapy was 2.5 months, and the median overall survival (OS) reached 8.5 months. Among 11 evaluable patients, 2 patients achieved partial response (PR), 5 patients achieved stable disease (SD), and the most common AEs were mainly hematologic toxicities^[12]^. In a phase I study of tremelimumab combined with gemcitabine in the treatment of advanced pancreatic carcinoma (untreated patients), the median OS reached 7.4 months, and among 28 evaluable patients, 2 patients achieved PR, and 7 patients achieved SD^[13]^. An Ib/II phase study evaluated the efficacy and safety of pembrolizumab combined with gemcitabine and nab-paclitaxel in the treatment of advanced or pancreatic carcinoma metastatic (untreated), enrolling 12 patients. The results showed that 25% of patients achieved PR and 67% achieved SD; PFS and OS were 9.1 months and 15.0 months, respectively, demonstrating promising efficacy, and Grade 3 AEs occurred in only 53% of patients^[14]^. In a phase I study of nivolumab combined with nab-paclitaxel and gemcitabine as first-line treatment for locally advanced or pancreatic carcinoma metastatic, among 50 patients, 2% achieved complete response (CR), 16% achieved PR, and 46% achieved SD; PFS and OS were 5.5 and 9.9 months, respectively^[15]^. At the 2020 ESMO conference, a randomized phase II clinical trial was reported on gemcitabine + nab-paclitaxel + durvalumab (D) + tremelimumab (T) as first-line treatment for ductal adenocarcinoma of pancreas metastatic. The mOS times for the AG group and the AG+D+T group were 8.8 months and 9.8 months, respectively; the mPFS times were 5.4 months and 5.5 months, and the ORR were 23.0% and 30.3%, respectively. There were no significant statistical differences in these three parameters. The DCRs of the AG group and the AG+D+T group were 57.4% and 70.6%^[16]^. Although adding D+T on the basis of the AG regimen did not significantly improve patients' OS, PFS, and ORR, the DCR of the AG+D+T group showed a trend of improvement. At the 2020 CSCO academic conference, Professor Liwei Wang's team presented the preliminary study results of a prospective single-arm exploratory clinical study on the efficacy and safety of camrelizumab combined with nab-paclitaxel and gemcitabine as first-line treatment for pancreatic carcinoma metastatic, introducing the phased study outcomes of camrelizumab combined with chemotherapy as first-line treatment for pancreatic carcinoma metastatic. For 12 patients who had undergone at least one imaging assessment, preliminary results showed that the objective response rate (ORR) reached 75%, and the disease control rate (DCR) reached 83.3%^[17]^. ICIs combined with chemotherapy have shown promising efficacy in the treatment of pancreatic carcinoma, but current studies are still mainly small-sample studies, and more clinical study is needed to verify efficacy and safety.

Patients with advanced pancreatic carcinoma who have not progressed after first-line treatment (including ① pancreatic carcinoma metastatic after systemic chemotherapy or ② locally advanced pancreatic carcinoma after concurrent chemoradiotherapy), may consider maintenance therapy if their performance status is good. Olaparib has been approved by the U.S. Food and Drug Administration (FDA) for maintenance therapy in patients with pancreatic carcinoma metastatic carrying germline *BRCA* mutations, who have not progressed after receiving at least 16 weeks of first-line platinum-based chemotherapy. In addition, other maintenance regimens that can be tried clinically include: (1) FOLFIRI or FOLFOX regimen for maintenance therapy after the FOLFIRINOX regimen; (2) 5-FU/LV or capecitabine for maintenance therapy after the FOLFIRINOX regimen; (3) single-agent GEM for maintenance therapy after GEM combined with nab-paclitaxel; (4) S-1 for maintenance therapy after nab-paclitaxel combined with S-1. However, the above regimens still require validation by high-level clinical trial results.

## Physicochemical Properties and Pharmaceutical Characteristics of the Investigational Product

Camrelizumab (R & D code: SHR-1210) is a humanized anti-programmed cell death receptor 1 (PD-1) antibody. It is an original drug independently developed by Jiangsu Hengrui Pharmaceuticals Co., Ltd., and Hengrui holds the global development rights to Camrelizumab.

English Generic Name: Camrelizumab for Injection

Laboratory Code: SHR-1210

Molecular Formula: C_6390_H_9838_N_1678_O_2004_S_46_

Molecular Weight: 143,708

**Physical Properties:** Camrelizumab for clinical use is a white or off-white lyophilized powder, used after reconstitution. When reconstituted with sterile water for injection, the preparation has a pH value of 5.0 to 5.8 and an osmotic pressure of 270 mOsm/kg to 330 mOsm/kg.

**Chemical Properties:** Camrelizumab is a humanized monoclonal antibody (IgG4, IgK) expressed in Chinese hamster ovary (CHO) cell lines using recombinant technology. Camrelizumab contains 1,314 amino acids, with a molecular weight of 143,708 Daltons, and a molecular formula of C_6390_H_9838_N_1678_O_2004_S_46_. Its molecule is a heterotetramer containing two identical heavy chains and two identical light chains, covalently linked via intra-chain and inter-chain disulfide bonds. Each heavy chain consists of 443 amino acids, and each light chain consists of 214 amino acids. Each heavy chain is linked via N-glycosylation at the typical glycosylation site of asparagine at position 293, and is predominantly a C-terminal lysine residue.

Camrelizumab preparation is a white or off-white lyophilized powder with a dosage of 200 mg per vial, packaged in 20 mL USP Type I glass vials, and the concentration of the active pharmaceutical ingredient is 40 mg/mL (the buffer consists of 10 mM sodium acetate, 9% (w/v) α,α-trehalose dihydrate, and 0.02% (w/v) polysorbate 20, with a pH value of 5.0 to 5.8). Each vial of preparation is reconstituted with 5.0 mL of sterile water for injection, resulting in a concentration of 40 mg/mL after dissolution. The reconstituted preparation is further diluted in an infusion bag with an intravenous injection mixture containing 5% glucose or 0.9% sodium chloride to a final concentration of 0.5 mg/mL to 10 mg/mL.

Surufatinib is a small molecule kinase inhibitor that primarily acts on vascular endothelial growth factor receptors (VEGFR1, 2, 3), fibroblast growth factor receptor (FGFR1), and colony-stimulating factor 1 receptor (CSF1R). It is a patented product exclusively developed by Hutchison Whampoa Pharmaceuticals (Shanghai) Co., Ltd.

English Generic Name: Surufatinib Capsules

Laboratory Code: HMPL-012

Chemical Name:

N-(2-(dimethylamino)ethyl)-1-(3-(4-(2-methyl-1H-indol-5-yloxy)pyrimidin-2-ylami no)phenyl)methanesulfonamide

Molecular Formula: C_24_H_28_N_6_O_3_S

Molecular Weight: 480.59

This product is in capsule form, and the strength is 50 mg, containing white or off-white powder; odorless. The active ingredient of this product is insoluble in water, slightly soluble in methanol and ethanol.

The melting point of the active ingredient is 162-170 °C.

## Preclinical Study Summary

### Preclinical Pharmacodynamic Studies

The study results on the inhibitory activity of surufatinib against 67 kinases showed that surufatinib had strong inhibitory effects on tumor angiogenesis kinases VEGFR1, VEGFR2, VEGFR3, FGFR1, and CSF1R, with half-maximal inhibitory concentrations of 2, 24, 1, 15, and 4 nM, respectively. The inhibition against the other kinases was relatively weak, with most half-maximal inhibitory concentrations greater than 100 nM, demonstrating good selectivity.

At the cellular level, surufatinib significantly inhibited the phosphorylation of VEGFR2 after VEGF stimulation and the proliferation of VEGF-dependent HUVEC cells, with half-maximal inhibitory concentrations of 3 nM and 16 nM, respectively; whereas its direct cytotoxicity toward HUVEC cells and other tested human tumor cells was relatively weak, with half-maximal inhibitory concentrations all greater than 5000 nM. At the ex vivo organ tissue level, surufatinib also inhibited the formation of microvessels in rat aortic ring assays, with an IC50 of 192 nM.

In vivo studies have shown that surufatinib can inhibit VEGFR2 phosphorylation in the vasculature of pulmonary tissue stimulated by VEGF with dose dependence. Plasma concentrations greater than 181 ng/mL can completely inhibit VEGFR2 phosphorylation, and the inhibitory effect of 20 mg/kg can last for 4 hours. Therefore, a twice-daily dosing regimen was adopted in the pharmacodynamic studies of tumor inhibition in nude mice.

In subcutaneous tumor transplant models in nude mice of human gastric cancer BGC-823, colon cancer HT-29, non-small cell lung cancer H460 and renal cancer Caki-1, surufatinib can inhibit tumor growth with dose dependence, with a minimum effective dose of 20 mg/kg administered twice daily. Pharmacokinetic and pharmacodynamic correlation studies also indicated that the tumor inhibitory effect of surufatinib increased with increased drug exposure. In the most sensitive tumor strain, gastric cancer BGC-823, at high doses, most animals exhibited a decrease in tumor volume (a mean volume reduction of 20% compared to the initial measurement). In the inhibited tumor tissues, surufatinib reduced the area of CD31-positive cells, which were vascular endothelium-specific surface markers, with dose dependence, suggesting that angiogenesis in tumor tissues was inhibited.

These studies suggested that surufatinib was promising for clinical use in the treatment of various solid tumors.

### Preclinical Toxicology Studies

1.3.2.1 Safety Pharmacology

In hERG assays, five points were tested at concentrations ranging from 0.003 to 30 μM, with 2-3 cells per point. The results showed that the IC_50_ of surufatinib for inhibition of the hERG channel was approximately 4.9-6.8 μM, indicating no significant inhibitory effect on the hERG channel.

Safety pharmacology studies found that surufatinib had no significant effects on the respiratory and cardiovascular systems of anesthetized Beagle dogs; it also had no impact on motor coordination and behavioral activities in ICR mice.

1.3.2.2 Acute Toxicity Study

An acute toxicity study in rats was conducted according to the "Fixed Dose Method"; 20 SD rats were randomly divided into 2 groups of 10 rats each, with equal numbers of males and females. Surufatinib was administered orally once at doses of 0 (0.5% CMC) and 2000 mg/kg. The main toxic symptoms were diarrhoea and slow weight gain in male rats; no deaths occurred, and no abnormalities were observed upon gross necropsy. The maximum tolerated dose (MTD) was 2000 mg/kg.

An acute toxicity study was conducted in 2 Beagle dogs (1 male and 1 female), with a single oral administration of surufatinib

at a dose of 2000 mg/kg. Animals were closely observed for 6 hours post-dosing, followed by continuous observation for 14 days. The main toxic reactions were diarrhoea, vomiting, and reduced food intake; no deaths occurred, and no abnormalities were observed upon gross necropsy. The MTD was 2000 mg/kg.

1.3.2.3 A Two-Week Chronic Toxicity Study of Surufatinib in Rats with a Four-Week Recovery Period

Four dose groups of 0, 20, 60 and 120 mg/kg were set up in the chronic toxicity study in rats, with 20 animals in each main test group and 6 animals (half males and half females) in the satellite TK test group; 5/10 animals of each sex in each group were necropsied at the end of the dosing period (except 5/9 females in the 60 mg/kg group, 2/5 females and 3/3 males in the 120 mg/kg group), and the remaining animals in each group were necropsied at the end of the recovery period. The main toxicities were: the test article at doses above 60 mg/kg caused hunched posture, muzzle contamination, slowed or even decreased weight gain, decreased activity and food intake, diarrhoea, liver and kidney injury, bile duct dilatation, thymus atrophy, gastrointestinal hemorrhage, and slight decreases in platelets, which resulted in animal death. The test article at dose 120 mg/kg caused decreased weight and anemia in females; slow body weight gain, liver and kidney injury, thymus atrophy and intestinal hemorrhage were still observed after 2 weeks of treatment withdrawal, and broken teeth was observed in males. The main target organs of toxicity were liver, kidney, thymus, and gastrointestinal tract. Under the conditions of this study, the no-observed-adverse-effect level (NOAEL) was 20 mg/kg, which is the equivalent pharmacodynamic dose in rats.

1.3.2.4 A Two-Week Chronic Toxicity Study of Oral Surufatinib in Beagle Dogs with a Four-Week Recovery Period

In the chronic toxicity study in Beagle dogs, five dose groups of 0, 6, 12, 24 (decreased to 18 on Day 15), and 36 mg/kg (1, 2, 4, and 6 times the equivalent pharmacodynamic dose) were set up with 6 animals per group, half males and half females; 4/6 and 2/6 animals (1/3 and 2/3 animals in the 24 mg/kg group; surviving animals in the 36 mg/kg group were necropsied early to Day 12 due to poor general condition) were sacrificed at 4 weeks of dosing and after the end of the recovery period. Animals were observed twice daily and weighed weekly during the dosing phase; toxicokinetic studies were performed on Days 1 and 28 in the dosing group (Days 1 and 11 in the 36 mg/kg group).The main toxicities were: test article at doses above 24 (18 on Day 15) mg/kg resulted in decreased weight, gastrointestinal reactions such as decreased food intake, diarrhoea, hematochezia and vomiting, and liver and kidney injury, which resulted in animal death; these reactions were recovered in animals in the 24 (18) mg/kg group after a 2-week recovery period. The main target organs of toxicity were the gastrointestinal tract, liver, kidney, and thymus. Although only some animals in the 12 mg/kg group showed positive reactions for protein urine and no abnormalities were observed in pathology and blood chemistry, the possibility of renal injury was not excluded. The test article did not accumulate in animals after repeated dosing. Under the conditions of this study, the NOAEL was 6 mg/kg, which was the equivalent pharmacodynamic dose in dogs.

## Study Rationale

Surufatinib is a novel pyrimidine small-molecule targeted anti-tumor drug independently developed by Hutchison MediPharma. It can effectively inhibit the activities of VEGFR (1, 2, 3), FGFR1, and CSF1R, thereby inhibiting functions such as vascular endothelial cell proliferation and lumen formation, ultimately inhibiting tumor growth by suppressing the formation of tumor neovascularization. Study has found that endocrine gland cells and peptidergic neurons from which neuroendocrine tumors originate can synthesize and secrete a large number of pro-angiogenic molecules, which are secreted into the blood through abundant vasculature. Correspondingly, highly vascularized neuroendocrine tumors also secrete large amounts of pro-angiogenic factors such as VEGF-A, VEGF-C, and FGF2, especially in pancreatic neuroendocrine tumors. In a Phase I dose escalation clinical study, among 18 evaluable neuroendocrine tumor patients treated with surufatinib, 8 patients achieved PR (44.4%), including 3 out of 7 evaluable PNET (pancreatic neuroendocrine tumor) patients achieving PR (42.9%).Additionally, the Phase Ib/II extension study conducted in neuroendocrine tumor patients showed a similar efficacy trend, demonstrating the preliminary efficacy of surufatinib in advanced NETs. In terms of safety, data from Phase Ia clinical trials indicated that the adverse reactions of surufatinib were similar to those of similar VEGFR-targeted therapies, with no new safety information discovered. These adverse reactions were mostly mild to moderate, with a low incidence of therapy interrupted or dose reduction, and could be alleviated or recovered through symptomatic treatment. The most common adverse reactions in the study included diarrhoea, hypertension, proteinuria, abdominal discomfort, AST increased, blood albumin decreased, fatigue, etc. Their incidence and severity were similar to or slightly lower than those of similar VEGFR-targeted drugs; adverse reactions frequently occurring with similar targeted drugs, such as hand-foot syndrome, were uncommon during surufatinib treatment, and serious adverse reactions commonly seen with VEGFR-targeted drugs, such as thrombosis and embolism, were not observed. Safety data from six clinical studies involving a total of 407 patients with advanced tumors showed that Grade 3 and above adverse reactions with an incidence ≥2% included: hypertension (29.5%), proteinuria (15.2%), blood bilirubin increased (8.6%), anaemia (6.9%), blood uric acid increased (5.4%), blood triglycerides increased (4.7%), aspartate aminotransferase increased (4.2%), hemorrhage (excluding laboratory test abnormal) (3.4%), diarrhoea (3.2%), blood potassium decreased (3.2%), alanine aminotransferase increased (2.7%), white blood cell count decreased (2.5%), blood sodium decreased (2.2%), and blood phosphorus decreased (2.0%). Surufatinib has now been approved by the National Medical Products Administration (NMPA) of China for the treatment of non-pancreatic neuroendocrine tumors. This approval is based on the renowned SANET-ep study, a randomized, double-blind, placebo-controlled Phase III clinical trial led by Professor Jianming Xu of the Fifth Medical Center of Chinese PLA General Hospital, involving 24 hospitals in China and enrolling a total of 198 patients with unresectable or metastatic, well-differentiated non-pancreatic neuroendocrine tumor (NET) with good physical condition (ECOG PS score 0 or 1), who had progressed after up to two prior systemic treatment regimens. Patients were assigned in a 2:1 ratio, with 129 cases in the surufatinib group and 69 cases in the placebo group. The primary endpoint was progression-free survival (PFS) assessed by investigators. At the ESMO conference in September 2019, Professor Jianming Xu orally reported the initial results of the study: the objective response rate (ORR) in the surufatinib group reached 10.3%, tumor regression rate was 61.2%, and disease control rate (DCR) was 86.5%. In September 2020, the full text of the SANET-ep study was published online in "Lancet Oncology". The median follow-up times for the surufatinib and placebo groups were 13.8 months and 16.6 months, respectively, with investigator-assessed median PFS of 9.2 months and 3.8 months, respectively; surufatinib significantly reduced the risk of disease progression or death by 67%. This interim analysis met the pre-specified criteria for early discontinuation of the study, and thus the study was discontinued early with approval from the Independent Data Monitoring Committee. At the 2020 ASCO annual meeting, Professor Ming Lu from Beijing Cancer Hospital presented the results of a Phase I clinical study of surufatinib combined with toripalimab in the treatment of advanced solid tumors. The study enrolled a total of 30 patients with unresectable metastatic solid tumors who had experienced treatment failure of standard therapy or had no standard treatment regimen, among whom 1 achieved complete response (CR) and 9 achieved partial response (PR). In the surufatinib 250 mg group, the ORR reached 63.6% and the DCR reached 100%. These results preliminarily indicated that surufatinib combined with ICIs has good anti-tumor activity in solid tumors and has good tolerability[18].

VEGF family members are overexpressed in pancreatic ductal adenocarcinoma (PDAC) and play an important role in the malignant progression of PDAC. VEGFR1 and VEGFR2 have been found to be overexpressed in PDAC and promote tumor progression. VEGFR1 has also been found to induce epithelial-mesenchymal transition (EMT), a process whereby polarized epithelial cells acquire mesenchymal and invasive phenotypes. VEGFR2 can enhance the local invasiveness of PDAC cells. The above suggests that VEGF-targeted therapy may block the proliferation, survival, and motility of PDAC cells^[19, 20]^. Therefore, this study aims to explore the efficacy and safety of surufatinib combined with camrelizumab and AS first-line chemotherapy regimen compared with the GN regimen in unresectable locally advanced or pancreatic carcinoma metastatic, providing clinical evidence for first-line treatment of pancreatic carcinoma with anti-angiogenesis combined with immunotherapy and chemotherapy.

# Study Endpoints

## Primary Study Endpoints

- The primary endpoints of the Phase Ib study are the DLTs and RP2D of surufatinib combined with camrelizumab and the AS regimen as first-line treatment for advanced pancreatic carcinoma metastatic;

In this trial, DLTs are defined as any of the following AEs (NCI CTCAE 5.0) related to the product occurring during the study:

1. Non-hematologic toxicities:

1) Non-hematologic toxicities of Grade ≥3, with the following exceptions: nausea, vomiting, diarrhoea, constipation, electrolyte level decreased within one week after supportive care; Grade 3 fatigue ≤ 7 days of treatment; Grade 3 hypertension that can be controlled by drugs within 1 week; alopecia, fever, and increased alkaline phosphatase due to tumor or infection;

2) Decrease in ventricular ejection fraction of Grade ≥ 3;

2. Hematologic toxicities:

1) Regarding possible AEs, even with symptomatic treatment, the following still occurs: Grade 4 neutropenia, thrombocytopenia, and hemoglobin decreased confirmed by at least 2 tests in 2 days; Grade 3 thrombocytopenia with hemorrhage tendency confirmed by at least 2 tests within 2 days;

2) Regarding possible AEs, even with symptomatic treatment, the following still occurs: Grade 3 neutropenia with fever (neutrophil count < 1.0 × 10^9^/L; body temperature ≥ 38.5 °C) confirmed at least twice in 2 days.

- The primary endpoint of the Phase II study is to evaluate the objective response rate (ORR) of first-line treatment with surufatinib combined with camrelizumab and the AS regimen in advanced pancreatic carcinoma metastatic.

## Secondary Study Endpoints

- To evaluate the progression-free survival (PFS), disease control rate (DCR), overall survival (OS), and safety of first-line treatment with surufatinib combined with camrelizumab and the AS regimen in advanced pancreatic carcinoma metastatic.

# Study Design

## Overall Design

This study is a prospective, single-center, randomized controlled Phase Ib/II study, enrolling patients with unresectable locally advanced or pancreatic carcinoma metastatic who have not received prior systemic therapy. In the Phase Ib stage, 9-15 subjects will be recruited using a 3+3 dose escalation scheme to explore the DLTs and RP2D of surufatinib combined with camrelizumab and the AS regimen. In the Phase II dose expansion stage, 90 subjects will be recruited and randomly assigned in a 1:1 ratio into the test group (45 cases) or the control group (45 cases), receiving surufatinib combined with camrelizumab and the AS regimen or standard first-line GN chemotherapy, until intolerable toxicity, disease progression, or death, to evaluate efficacy and safety.

This study is divided into three phases: screening phase, treatment phase, and follow-up phase. Every 6 weeks (±2 days), imaging methods will be used to assess the tumor status until disease progression (RECIST 1.1), death (during the patient’s treatment), or intolerable toxicity occurs, and record the tumor treatment and survival status after disease progression. Safety observations included: AEs, changes in laboratory values, vital signs, and changes in electrocardiograms, etc.

Table 2 Definition of Time for the 3 Periods During the Study

| Screening | Treatment Period | Follow-up Period |
| --- | --- | --- |
| Day -21 to Day -1  (Before the first dose of investigational product on Day 1) | From Cycle 1 Day 1 to the end of treatment (EOT, including one week off) | End of treatment to end of study |

# Subject Selection and Withdrawal

Inclusion of eligible subjects is a key step to achieve the intended objectives of this clinical study. Subjects must meet the following criteria to be allowed to participate in this study. All medical or non-medical conditions of each subject will be considered for his/her eligibility.

Before a subject is included in the study, the investigator should review, confirm, and document the suitability of the subject to participate in the study.

## Inclusion Criteria

Patients must meet all the following criteria to be enrolled in this study:

1. Have fully understood this study and voluntarily signed the informed consent form;
2. Patients with histologically or cytologically confirmed unresectable, locally advanced, or metastatic pancreatic ductal adenocarcinoma;
3. Age 18-75 years old (inclusive);
4. No prior systemic therapy for advanced pancreatic carcinoma;
5. ECOG PS 0-1;
6. Must have at least one measurable lesion, with the longest diameter of at least 10 mm as measured by spiral CT scan, or at least 20 mm as measured by conventional CT scan (according to Response Evaluation Criteria in Solid Tumors, i.e. RECIST v1.1);
7. Expected survival ≥ 3 months;
8. The functions of vital organs meet the following requirements (the use of any blood components and cell growth factors within *14 days before enrollment is not allowed):

Absolute neutrophil count ≥1.5×10^9^/L^;^

Platelets ≥100×10^9^/L^;^

Haemoglobin ≥90 g/L;

Total bilirubin < 1.5 × ULN;

ALT and/or AST < 1.5 × ULN ( < 3 × ULN for patients with metastases to liver);

Serum creatinine < 1.5 × ULN;

Endogenous creatinine clearance ≥50 mL/min;

1. Women of childbearing potential must use effective contraceptive measures;
2. Good compliance and cooperative with follow-up.

## Exclusion Criteria

Patients who meet any of the following criteria will not be enrolled in this study:

1. Unable to comply with the study protocol or study procedures;
2. Previously received treatment with VEGFR inhibitors, or previously used ICI treatment;
3. Participating in or having participated in other drug clinical trials within 4 weeks prior to enrollment;
4. Have received transfusion therapy, blood products, and hematopoietic factors, such as albumin and G-CSF, within 14 days prior to enrollment;
5. Brachytherapy (radioactive seed implantation) within 60 days prior to enrollment;
6. Have received other systemic anti-tumor treatments within 4 weeks prior to enrollment, including chemotherapy, signal transduction inhibitors, hormone therapy, and immunotherapy;
7. Have received any surgery or invasive treatment or procedure within 4 weeks prior to enrollment (excluding intravenous catheterization, paracentesis drainage, etc.);
8. Undergone major surgery within 60 days prior to enrollment or the surgical incision has not completely healed;
9. Have received local anti-tumor treatments within 4 weeks prior to enrollment, such as hepatic arterial interventional embolism, cryoablation or radiofrequency ablation of metastases to liver;
10. The patient currently has hypertension uncontrolled by medication, defined as: blood pressure systolic ≥140 mmHg and/or blood pressure diastolic ≥90 mmHg;
11. Protein urine ≥2+, or 24-hour protein urine amount ≥1.0 g on urinalysis;
12. Uncontrollable malignant ascites (defined as ascites that cannot be controlled by diuretics or paracentesis as judged by the investigator);
13. Clinically significant electrolyte abnormalities as judged by the investigator;
14. Liver metastases accounted for half or more of the total liver volume as determined by the investigator;
15. Clinically significant cardiovascular disorders, including but not limited to acute myocardial infarction within 6 months prior to enrollment, severe/unstable angina pectoris, or coronary artery bypass surgery; cardiac failure congestive with NYHA classification > Class II; ventricular arrhythmia requiring drug therapy; LVEF (left ventricular ejection fraction) <50%;
16. Hemorrhage events of ≥ Grade 3 occurring within 4 weeks prior to enrollment;
17. Patients who, within 3 months prior to enrollment, have clear evidence or history of haemorrhagic tendency (hemorrhage >30 mL within 3 months, occurrence of haematemesis, melena, haematochezia), haemoptysis (fresh blood >5 mL within 4 weeks), or have experienced thromboembolic events within 12 months (including stroke events and/or transient ischaemic attack);
18. INR > 1.5 or APTT > 1.5×ULN, or the patient is currently taking anticoagulants;
19. Currently, the patient has poorly controlled diabetes mellitus (after standard treatment, fasting glucose concentration ≥ CTCAE Grade 2);
20. The patient currently has any disease or condition that affects drug absorption, or the patient is unable to take surufatinib orally;
21. Active or uncontrolled severe infection (≥ CTCAE Grade 2 infection);
22. Known HIV infection;
23. Known history of clinically significant liver disease, including hepatitis viral [subjects known to be carriers of HBV must be excluded if they have active HBV infection, i.e., HBV DNA positive (>1×10^4^ copies/mL or >2000 IU/mL); known HCV infection with HCV RNA positive (>1×10^3^ copies/mL)], or other hepatitis, hepatic cirrhosis;
24. The patient currently has CNS metastasis or a history of brain metastasis;
25. Patients who currently have gastrointestinal diseases such as active gastric and duodenal ulcer, colitis ulcerative, or active hemorrhage in unresected tumor, or other conditions that may cause gastrointestinal hemorrhage or perforation as determined by the investigator;
26. Unresolved toxicities higher than CTCAE Grade 1 caused by any prior anti-cancer treatments, excluding alopecia and ≤ Grade 2 neurotoxicity caused by oxaliplatin;
27. Patients with a known or suspected allergy to the investigational product or drugs of the same class;
28. Pregnant (positive pregnancy test before medication) or breastfeeding women;
29. Drug abuse, medical, psychological, or social conditions may affect patient enrollment and the evaluation of experimental results;
30. Having other untreated or concomitant tumors, except cervical carcinoma in situ, treated basal cell carcinoma, or superficial bladder tumors. Patients can be enrolled if the tumor has been radically resected and there is no evidence of disease for more than 3 years. Treatment for all other tumors must have been completed at least 3 years prior to enrollment;
31. Patients considered by the investigator to be inappropriate for enrollment in this study.

## Subject Completes Study Treatment, Withdraws from Study, or Discontinues Study Treatment

### Criteria for Completion of Study Treatment:

- Disease progression (including clinical progression);
- Subject death;
- The entire study is completed.

### Withdrawal from the Study

Subjects may voluntarily withdraw from the study at any time, or they may be withdrawn from the study by the investigator or sponsor due to safety concerns, behavioral reasons, or inability to follow the study visit schedule or procedures as required by the protocol at the study site.

Reasons for withdrawal from the study include:

- The subject withdraws informed consent of participating in the study, and rejects further follow-up;
- It is not in the subject’s best interest to continue the treatment per the investigator due to occurrence of any clinical AEs, laboratory test abnormalities or co-morbidities;
- Other conditions that are necessary for the subject to withdraw from the study in the opinion of the investigator, for instance, the subject losses the ability to express his/her will freely due to confinement or isolation;
- Lost to follow-up;
- Subject death;
- The sponsor discontinued the study.

### Discontinuation Criteria

Discontinuation of study treatment did not mean withdrawal from the study. Subjects who discontinue the study treatment should continue to complete the remaining study visits as required by the protocol. A subject must discontinue the study treatment if any of the following criteria is met:

- The subject requests investigational product discontinuation;
- Medical imaging or clinical features indicate disease progression, unless the subject meets the criteria for continued treatment after progression (as judged by the investigator);
- Subject becomes pregnant during the study;
- Any clinical AE, laboratory test abnormality, or other medical conditions occur, which may result in the subject no longer benefiting from continued treatment.
- The patient is unable to continue to participate in the study due to overall deterioration of health status;
- Lost to follow-up;
- Subject death;
- Other reasons that lead to treatment discontinuation in the investigator's opinion.

### Procedures for Withdrawal from the Study or Discontinuation of Study Treatment

Every effort must be made to complete the efficacy and safety examinations specified in the protocol at the time of withdrawal from the study or discontinuation from study treatment, and to complete the safety follow-up visit with complete documentation of AEs and outcomes. The investigator may suggest or provide new or alternative treatments to the subject based on the actual situation of the subject. Subjects without disease progression should continue to be followed up for radiological evaluation until the subject starts a new anti-tumor treatment or disease progression.

If a subject refuses to come to the study site for further visits, study-related information should still be continuously collected unless the subject withdraws consent for further disclosure of information or further contact. In this case, no further study evaluations should be conducted, nor should any more data be collected.

## Dropout Criteria

All subjects who have signed the informed consent form and passed screening to enter the study have the right to withdraw from this study at any time.

- For subjects who have only signed the informed consent form and passed screening but have not been enrolled, they will not be considered as dropouts.
- After enrollment, any subject who does not complete the first two cycles of the trial and therefore cannot be evaluated for efficacy will be considered as a dropout. Except for the following reasons:
  1. After enrollment, subjects who experience disease progression with clear medical evidence are not considered as dropouts; however, radiological evidence must be provided;
  2. Subjects who discontinue participation due to pregnancy or intolerable toxicity should not be considered as dropouts.
  3. The investigator determines that treatment should be discontinued in the best benefit of the subject;
  4. Death after enrollment.

For dropout subjects, the investigator must record the reason for dropout, complete the relevant tumor assessments as much as possible, and record the circumstances of the last visit.

## Restrictions during the Study

Male or female patients of childbearing potential must use effective methods of contraception, such as double-barrier contraception, condoms, oral or injectable contraceptives, intrauterine devices, etc., during the trial (from signing the informed consent form) and within 90 days after the last dose. All female patients will be considered of childbearing potential unless the female patient has been naturally menopausal, has undergone artificial menopause, or sterilization (e.g., hysterectomy, bilateral adnexectomy, or radiation ovarian irradiation, etc.).

## Early Discontinuation or Suspension of the Study

The study may be discontinued or interrupted early if there are sufficient reasons. This may be due to decision from regulatory agencies, changes in opinions of EC, issues with the efficacy or safety of the investigational product, or the decision of the investigator. The party deciding to interrupt/discontinue the study will issue a written notice and record the reason for the discontinuation or suspension of the study to the investigator, the sponsor and the regulatory authority. The investigator must immediately notify the EC and the sponsor, and provide the relevant reasons. If a subject has been enrolled in an interventional clinical study that has been interrupted or discontinued, the institution and the investigator should formulate a protocol to properly safeguard the rights and interests of the enrolled subjects.

Reasons for early discontinuation or suspension of the study may include:

- Identified unexpected, significant, or unacceptable risks to the subjects.
- Existing efficacy results, which support early discontinuation of the study.
- Low compliance with protocol requirements.

If the abovementioned drug safety, protocol compliance issues causing study suspension are resolved, the study can be continued with the consent from the sponsor, EC or regulatory authorities.

# Treatment Regimen

## Name and Source of Investigational Product

### Drug Source

Surufatinib is provided by Hutchison Whampoa Pharmaceuticals (Shanghai) Co., Ltd. Camrelizumab is provided by the drug manufacturer (Suzhou Suncadia Biopharmaceuticals Co., Ltd.). Gemcitabine, nab-paclitaxel, and S-1 are locally sourced commercial drugs.

### Drug Dosage Form, Strength and Storage Conditions

Camrelizumab for injection is manufactured and packaged by Suzhou Suncadia Biopharmaceuticals Co., Ltd. Camrelizumab for injection is stored and transported at 2-8°C, protected from light. The drug preparation is carried out according to the instructions.

Surufatinib is packaged by qualified manufacturing facilities entrusted by Hutchison Whampoa Pharmaceuticals (Shanghai) Co., Ltd, with technical guidance and quality control from Hutchison Whampoa Pharmaceuticals (Shanghai) Co., Ltd.

**Table 3 Drug Dosage Form and Strength**

| **Dosage Form** | **Strength** | **Method of Administration** | **Manufacturer** |
| --- | --- | --- | --- |
| Capsule | 50 mg | Oral | Hutchison Whampoa Pharmaceuticals (Shanghai) Co., Ltd. |

Surufatinib should be stored in a safe and cool place as specified; stored at room temperature (10-30 °C), tightly closed and protected from light. The storage temperature needs to be recorded and kept in relevant documents. The strength, packaging and storage conditions of other investigational products will be subject to the specific product instructions.

### Investigational Product Randomization/Supply Scheme

After the investigator confirms that the subject meets all inclusion criteria and does not meet any exclusion criteria, they will enter IWRS. Eligible subjects will be randomized in a 1:1 ratio to the test group or control group for treatment. Each subject's randomization number will be provided to the investigator through IWRS.

After randomization, subjects must start the study treatment within 3 days.

### Drug Management

The registration and documentation of investigational products must be managed by a designated individual. The study site should ensure that there is a designee responsible for receiving the investigational products, that the drug distribution has accurate records, and that the investigational products are properly used and stored. The unused investigational products should be preserved according to the requirements of drug storage, and check whether the storage meet the requirements at regular intervals by designated personnel. After the study is completed, the remaining investigational products should be recovered, registered and recorded, and disposed of with a GCP compliant method.

## Method of Administration

### Dosing Regimen and Dosing Period

Test Group: surufatinib (200 mg/250 mg/300 mg orally, once daily continuous administration, with treatment cycles every 3 weeks) combined with camrelizumab (200 mg, intravenous infusion, q3w) and the AS three-week regimen (nab-paclitaxel: 125 mg/m^2^, I.V., on days 1 and 8; S-1: 40 mg BID orally, days 1-14, q3w), until intolerable toxicity or disease progression, death, or meeting other protocol-specified criteria for discontinuation of study treatment.

Control group: GnP 3-week regimen (nab-paclitaxel: 125 mg/m^2^, IV, d1, d8, q3w; gemcitabine: 1000 mg/m^2^, IV infusion over 30 minutes, d1, d8, q3w), until intolerable toxicity or disease progression, death, or reaching other criteria specified in the protocol for discontinuation of study treatment.

The above medications may be adjusted based on the adverse reactions of the subjects according to the protocol. Subjects will continue the treatment until disease progression, intolerable toxicity, withdrawal of informed consent, or discontinuation at the discretion of the investigator. The cycle date will be determined from the date of the first dose of the subject. In case of any dose interruption, omission, or underdose of the investigational product during the study, the treatment will continue according to the cycle specified in the protocol without supplementation or modification of cycle. However, it should be recorded in detail in the original data: if there is a drug omission, the time and the reason for the missed dose should be recorded in detail; if the drug is underdosed due to various reasons such as adverse drug reactions, it should be recorded in the subject's diary, original medical records, and eCRF.

### Method of Administration

Surufatinib: it is recommended to take the investigational product with 200 mL of clear water within 1 hour after breakfast, striving to take the medication at the same time each day. If vomiting occurs after medication administration, it is not recommended to take the medication again unless the entire capsule is seen.

During the study, every effort should be made to ensure that the patient is dosed according to the protocol. If the patient misses the dose in the morning, it can be taken at any time before 10 p.m. on the same day. However, if the patient misses the prescribed medication and fails to take it on the same day, the patient must take the prescribed dose at the next time, but the missed dose will not need to be taken again. The investigator should record the actual dose and amount of drug taken by the patient, and record it in the original diagnosis and treatment record and CRF.

Intense exercise should be avoided during the trial; smoking, alcohol, and caffeinated beverages should be avoided. During the study participation, patients should avoid drinking high concentrations of grapefruit juice and consuming grapefruit, lime, and beverages containing these ingredients.

S-1 is administered orally. Generally, the initial dose for adults is determined according to body surface area as per the table below. Dosage is given orally twice daily after breakfast and dinner for 28 consecutive days with 14 days of rest as a treatment cycle. Doses are administered until the patient's condition worsened or the patient is no longer tolerable.

## Precautions

According to the latest Investigator's Brochure for surufatinib, the following toxicities need to be particularly observed and prevented in this clinical study.

**Hypertension**

Hypertension is one of the common adverse reactions during surufatinib treatment. It can occur at any time after the start of treatment, but most cases appear within one month after administration. Most of hypertension are Grade 1-2; some patients experience Grade 3 increased blood pressure, but there were no cases of Grade 4 or higher hypertension. Blood pressure should be actively monitored during treatment, and patients who develop increased blood pressure should be promptly treated. Currently, there are no prospective clinical studies confirming the optimal treatment regimen for hypertension induced by VEGF inhibitors. Therefore, the choice of antihypertensive drugs should consider the patient's specific clinical situation. For patients with proteinuria, chronic kidney disease, or metabolic diseases, ACE inhibitors (ACEI) or angiotensin II receptor blockers (ARB) class antihypertensive drugs are preferred. Calcium channel blockers are mainly metabolized via CYP3A4; when used in combination with surufatinib, blood pressure should be monitored to prevent hypotension. Please refer to hypertension treatment guidelines, and consult a cardiologist when necessary. The goal of antihypertensive treatment is to control blood pressure within the range of 140/90 mmHg; however, for high-risk populations, such as patients with chronic kidney disease, diabetes mellitus, or those who develop proteinuria after treatment, it is recommended to control blood pressure within the range of 130/80 mmHg.

**Proteinuria**

Proteinuria is one of the common adverse reactions during surufatinib treatment. Most of proteinuria are Grade 1-2, with a few reaching Grade 3. In the phase I clinical study of surufatinib, a small number of patients paused medication or discontinued treatment due to proteinuria. If a patient's urinalysis during treatment detects protein urine 2+, please collect a 24-hour urine, manage according to local standard medical practice based on the test results, and reduce the dose or stop the drug if necessary.

**Gastrointestinal Symptoms**

Common gastrointestinal symptoms during surufatinib treatment include diarrhoea, nausea, or vomiting. If a patient experiences diarrhoea, nausea, vomiting, symptomatic treatment with drugs may be given according to local medical practice, and blood electrolyte levels (e.g., 1-2 times a week) should be closely monitored, and fluid replacement supportive treatment should be given promptly. After appropriate treatment, the investigational product may be interrupted if diarrhoea, nausea and vomiting are still intolerable. Prophylactic symptomatic treatment may be considered in patients with a clear predisposition to diarrhoea, nausea, and vomiting.

If the investigator judges that diarrhoea is related to the toxicity of the investigational product, antidiarrheal and intestinal flora regulating medications such as montmorillonite powder and loperamide can be administered; if it is judged that the diarrhoea is mainly caused by hormones secreted by the tumor, short-acting octreotide acetate (0.1 mg every 8 hours, subcutaneous injection) can be given for symptomatic treatment. If control is poor, the use of long-acting somatostatin analogs (long-acting octreotide acetate 20 or 30 mg once every 28 days, intravenous injection, or lanreotide acetate for injection 40 mg once every 14 days, intravenous injection) can be considered for short-term symptomatic treatment.

**Bone Marrow Suppression**

Common myelosuppression during surufatinib treatment include leukopenia, anaemia, and thrombocytopenia. Hematology and coagulation tests should be closely monitored, and patients should be noted for hemorrhage symptoms. Leukopenia, anaemia, and thrombocytopenia are mostly Grade 1-2. If the patient experiences events of Grade 3 or above, discontinuation may be considered on a case-by-case basis, hematology may be repeated every 2-3 days or daily, and transfusion or platelet transfusion may be given if necessary.

**Hemorrhage**

Hemorrhage is one of the common adverse reactions during surufatinib treatment, mostly Grade 1-2. If the hemorrhage event is assessed as Grade 2, medication needs to be interrupted; if it can recover to Grade 1 or below within 28 days, medication can be resumed at a reduced dose. If the hemorrhage event is assessed as severe (≥ Grade 3) or an SAE, surufatinib treatment should be stopped immediately, and appropriate treatment methods should be taken to control the hemorrhage (e.g., transfusion, radiotherapy, endoscopy, or selective surgical interventions). When the patient cannot tolerate invasive procedures or surgery, the best supportive care is provided.

**Hepatic Function Abnormal**

Hepatic ALT, AST increased, and/or blood bilirubin increased have been observed, mostly Grade 1-2, with a few being Grade 3-4, and fatal Grade 5 liver injury events have been reported. In clinical study, liver function and skin color should be closely monitored. Immediate discontinuation is required, supportive care is given, and other potential causes of liver injury are identified by examination. Hepatic function abnormal conditions that should be reported immediately to the sponsor include: 1. Subjects with normal liver function at baseline (ALT, AST, and bilirubin all within normal ranges) who, in the same blood sample collection, have AST and/or ALT increased ≥3×ULN combined with total serum bilirubin increased ≥2×ULN. 2. Subjects with transaminases or total bilirubin increased at baseline who, in the same blood sample collection, have AST and/or ALT increased ≥2 times the baseline value combined with total serum bilirubin increased ≥2 times the baseline value.

**Acute Kidney Injury**

It includes acute kidney injury, acute renal failure, renal injury, blood urea increased, and blood creatinine increased, etc. Most acute kidney injury events are Grade 1-2, with a few being Grade 3-4. When patients experience decreased urine output, a 24-hour urine volume should be collected. After temporarily interrupting medication, if recovery to Grade 1 occurs within 28 days, study treatment is re-started at a reduced dose level. If recovery takes more than 28 days, it is generally necessary to discontinue study treatment. If patients develop hypertension before or during acute kidney injury, blood pressure should be controlled according to hypertension management measures.

**Other Important Potential Risks**

Some important potential risks have been observed from AE and laboratory abnormality data in preclinical studies or ongoing clinical studies: including infection, intestinal obstruction, gastrointestinal perforation, myocardial ischaemia, cardiac failure, venous thromboembolism/arterial thromboembolism, and pancreatitis. It is recommended to closely monitor patients for signs of these risks and provide supportive treatment according to local clinical practice.

In previous pancreatic carcinoma patients, the incidence of common toxic reactions during monotherapy with S-1 is as follows: leukopenia 32.2%, neutropenia 27.1%, haemoglobin decreased 50.8%, thrombocytopenia 33.9%, decreased appetite 61%, nausea 55.9%, pigmentation 39%, stomatitis 25.4%, vomiting 35.6%, AST increased 18.6%, and ALT increased 16.9%. Severe myelosuppression may occur such as pancytopenia, granulocytopenia (symptoms: pyrexia, sore throat, and general discomfort), leukopenia, anaemia, and thrombocytopenia (incidence as above), and haemolytic anaemia (incidence unknown). Severe hepatic function abnormalities like fulminant hepatitis (including those caused by reactivation of hepatitis B) may occur (incidence unknown); severe diarrhoea may cause dehydration (incidence unknown); severe enteritis incidence is 0.5%; haemorrhagic enteritis, ischaemic enteritis, and enteritis necroticans may occur. The incidence of interstitial pneumonia is 0.3%; early symptoms include cough, shortness of breath, dyspnoea, and pyrexia. Myocardial infarction, angina pectoris, arrhythmia (including ventricular tachycardia), and cardiac failure may occur (all with unknown incidence). In addition, serious stomatitis (incidence unknown), gastrointestinal ulcer (incidence 0.5%), gastrointestinal hemorrhage (incidence 0.3%), and gastrointestinal perforation (incidence unknown), etc. may occur.

In previous clinical studies of gemcitabine, common adverse reactions include: neutropenia: Grade 3 19.3%, Grade 4 6%; mild to moderate myelosuppression, thrombocytopenia, anaemia, febrile neutropenia, anorexia, headache, insomnia, somnolence, mild dyspnoea, cough, dermatitis, vomiting, diarrhoea, nausea, stomatitis, constipation, AST and ALT increased, alkaline phosphatase increased, skin pruritus, mild proteinuria, and fatigue.

The most common adverse reactions (≥20%) of nab-paclitaxel in adenocarcinoma pancreas are neutropenia, fatigue, peripheral neuropathy, nausea, alopecia, peripheral oedema, diarrhoea, pyrexia, vomiting, decreased appetite, rash, and dehydration.

AEs related to immunotherapy are usually temporary but can occasionally be severe or fatal. The most common and significant immunotherapy-related AEs are dermatosis, diarrhoea/colitis, hepatotoxicity, and endocrine diseases, but other organs may also be affected. In previous monotherapy studies of camrelizumab in other cancer types, the majority of reported adverse reactions are Grade 1 or 2 in severity. The most common ≥ Grade 3 adverse reactions are: anaemia (3.9%), hyponatraemia (3.1%), blood bilirubin increased (3.0%), gamma-glutamyltransferase increased (2.4%), and aspartate aminotransferase increased (2.2%).

## Dose Adjustment

### Principles of Dose Adjustment

Adverse drug reactions experienced by patients during the trial will be graded according to NCI CTCAE version 5.0. For subjects in the surufatinib treatment group, the dose should be adjusted according to the following rules:

- When intolerable adverse drug reactions occur during treatment, dose reduction may be performed, but each patient's dose reduction should not exceed 2 times.
- For complications already identified at baseline, if deemed appropriate by the investigator, dose adjustments should be made according to the corresponding change in the grade of adverse reactions. For example, if a patient presents with Grade 1 fatigue at baseline and it increases to Grade 2 during treatment, it can be considered a one-grade change in toxicity, and dose adjustment should be made according to Grade 1 toxicity.
- To allow recovery from acute adverse reactions, treatment can generally be delayed for up to 28 days unless otherwise specified. If treatment needs to be delayed for more than 28 days, the investigator must agree in advance with the sponsor.
- If multiple adverse reactions occur at the same time with different grades or severity, dose adjustment should be based on the highest observed grade.

### Detailed Procedures for Dose Adjustment

See Tables 4 and 5 for dose reduction guidelines for hematologic and non-hematologic toxicities of surufatinib (dose reduction guidelines related to hypertension, proteinuria, hepatic dysfunction, thrombocytopenia, and hemorrhage are found in the package insert). For each dose reduction, the single dose of surufatinib should be reduced by 50 mg, but the lowest dose should not be less than 150 mg.

**Table 4 Dose Adjustments for Hematological Toxicities**

| **NCI CTCAE v5.0 Toxicity Grade** | **Actions** |
| --- | --- |
| **Grade 1 or 2** | None |
| **Grade 3 or 4**  Expected to be treatable/reversible after dose reduction  Grade 3 or 4 toxicity recovered to ≤ Grade 1 or baseline level within 28 days_b_ | Interruption_a_  Reduction by one dose level |
| Recurrence of Grade 3 toxicity  Recurrence of Grade 4 toxicity | Reduction by one dose level or discontinuation of investigational product  Discontinuation of investigational product |
| **Grade 3 or 4**  Expected to be non-treatable/irreversible after dose reduction | Discontinuation of investigational product |

_a_ Interruption of the investigational product until toxicity recovers to ≤ Grade 1 or baseline level. For patients whose toxicity resolution exceeds 28 days, the study treatment should generally be discontinued; unless judged by the investigator that the patient still has clinical benefit, the investigator should agree with the sponsor whether the patient should continue study treatment.

_b_ Interruption of the investigational product when white blood cells or neutrophils decrease to Grade 3 or 4; after recovery to ≤ Grade 1 or baseline levels within 28 days, the original dosage can be maintained. A dose reduction is necessary only when febrile neutropenia (neutrophil count <1000/mm³, accompanied by pyrexia ≥38℃ lasting over 1 hour or a single body temperature measurement >38.3℃) occurs, or when Grade 4 neutropenia recurs or persists for up to 7 days and after recovery to ≤ Grade 1 or baseline levels within 28 days.

**Table 5 Dose Adjustments for Non-Hematological Toxicities**

| **NCI CTCAE v4.03** Toxicity Grade | **Actions** |
| --- | --- |
| **Grade 1 or 2** (tolerable) | None |
| **Grade 2** (intolerable)**_d_**  Toxicity recovered to ≤ Grade 1 or baseline level within 28 days  Relapse | Interruption_c_  Reduction by one dose level |
| **Grade 3 or 4_d_**  Expected to be treatable/reversible after dose reduction  Grade 3 toxicity recovered to ≤ Grade 1 within 28 days  Recurrence of Grade 3 toxicity  Recurrence of Grade 4 toxicity | Interruption_c_  Reduction by one dose level  Reduction by one dose level or discontinuation of investigational product  Discontinuation of investigational product |
| **Grade 3 or 4**  Expected to be non-treatable/irreversible after dose reduction | Discontinuation of investigational product |

_c_ For patients whose toxicity resolution exceeds 28 days, the study treatment should generally be discontinued; unless judged by the investigator that the patient still has clinical benefit, the investigator should agree with the sponsor whether the patient should continue study treatment.

_d_ For nausea, vomiting, or diarrhoea, supportive treatment should first be administered. Investigational product should be withheld until toxicity returns to ≤ Grade 1 or baseline. Additionally, for adverse reactions that the investigator believes are unlikely to develop into serious or life-threatening toxicity events and the patient can tolerate, under the premise of actively providing symptomatic treatment, the study treatment can be maintained at the same dose level without dose reduction or interruption, such as hyperuricaemia and hypophosphataemia found in laboratory tests, or alopecia, etc.

During treatment with camrelizumab, atypical responses may be observed (e.g., temporary enlargement of tumors or appearance of new small lesions in the first few months, followed by tumor shrinkage). If the patient's clinical symptoms are stable or continuously improving, even if there is preliminary imaging evidence of disease progression, based on the assessment of overall clinical benefit, consideration can be given to continuing treatment with camrelizumab until disease progression is confirmed or intolerable toxicity occurs.

Dose interruption or discontinuation may be required based on individual safety and tolerability. Dose increase or decrease was not recommended. Specific adjustment protocols for dose interruption or permanent discontinuation are shown in Table 6.

**Table 6 Recommended Dose Adjustment Plan for Camrelizumab**

|  | Severity‡ | Treatment Modification Plan |
| --- | --- | --- |
| **Reactive capillary endothelial proliferation*** | Grade 3 | Interrupt medication until the adverse reaction recovers to Grade 0-1. |
|  | Grade 4 | Permanent discontinuation |
| **Immune-Related Adverse Reactions** | | |
| Pneumonia | Grade 2 | Interrupt medication until the adverse reaction recovers to Grade 0-1. |
|  | Grade 3 or 4 or recurrent Grade 2 | Permanent discontinuation |
| Diarrhoea and colitis | Grade 2 or 3 | Interrupt medication until the adverse reaction recovers to Grade 0-1. |
|  | Grade 4 | Permanent discontinuation |
| Hepatitis  (Applicable to non-hepatocellular carcinoma patients) | Grade 2, aspartate aminotransferase (AST) and alanine transaminase (ALT) are at 3-5 × the upper limit of normal (ULN), or total bilirubin (TBIL) is at 1.5-3 × ULN. | Interrupt medication until the adverse reaction recovers to Grade 0-1. |
|  | Grade 3 or 4, AST, ALT >5 × ULN, or total bilirubin >3 × ULN | Permanent discontinuation |
| Hepatitis  (Applicable to hepatocellular carcinoma patients) | AST or ALT: 3-5 × ULN, if baseline is within normal range  AST or ALT: 5-10 × ULN, if baseline AST or ALT is between 1-3 × ULN  AST or ALT: 8-10 × ULN, if baseline is 3-5 × ULN  Total bilirubin: 1.5-3 × ULN | Interrupt medication until the adverse reaction recovers to Grade 0-1 or returns to baseline, then resume dosing. |
|  | AST or ALT: >5 × ULN, if baseline is within normal range  AST or ALT: >10 × ULN, if baseline > ULN  Total bilirubin >3 × ULN | Permanent discontinuation |
| Nephritis | Grade 2 or 3 blood creatinine increased | Interrupt medication until the adverse reaction recovers to Grade 0-1. |
|  | Grade 4 blood creatinine increased | Permanent discontinuation |
| Endocrine diseases | Symptomatic Grade 2 or 3 hypothyroidism, Grade 2 or 3 hyperthyroidism, Grade 2 or 3 hypophysitis, Grade 2 adrenal insufficiency  Grade 3 hyperglycaemia or diabetes mellitus | Interrupt medication until the adverse reaction recovers to Grade 0-1. |
|  | Grade 4 hypothyroidism  Grade 4 hyperthyroidism  Grade 4 hypophysitis  Grade 3 or 4 adrenal insufficiency  Grade 4 hyperglycaemia or diabetes mellitus | Permanent discontinuation |
| Skin adverse reaction | Grade 3 | Interrupt medication until the adverse reaction recovers to Grade 0-1. |
|  | Grade 4, Stevens-Johnson syndrome (SJS) or toxic epidermal necrolysis (TEN) | Permanent discontinuation |
| Thrombocytopenia | Grade 3 | Interrupt medication until the adverse reaction recovers to Grade 0-1. |
|  | Grade 4 | Permanent discontinuation |
| Other immune-related adverse reactions | Grade 3 or 4 amylase increased or lipase increased  Grade 2 or 3 pancreatitis  Grade 2 myocarditis**  Other first occurrences of Grade 2 or 3 immune-related adverse reactions | Interrupt medication until the adverse reaction recovers to Grade 0-1. |
|  | Grade 4 pancreatitis or recurrent pancreatitis of any grade  Grade 3 or 4 myocarditis  Grade 3 or 4 encephalitis  Other first occurrences of Grade 4 immune-related adverse reactions | Permanent discontinuation |
| Recurrent or persistent adverse reaction | Recurrent Grade 3 or 4 (excluding endocrine disorders)  Grade 2 or Grade 3 adverse reactions (excluding endocrine disorders) that have not improved to Grade 0-1 within 12 weeks after the last administration  Failure to reduce corticosteroids to a prednisone-equivalent dose of ≤10 mg/day within 12 weeks after the last administration | Permanent discontinuation |
| Infusion reaction | Grade 2 | Reduce the infusion rate or interrupt administration, and the investigational product may be resumed when there is a symptomatic remission, closely observation should also be conducted. |
|  | Grade 3 or 4 | Permanent discontinuation |

Patients with mild hepatic impairment do not require dose adjustments. Use in patients with moderate or severe hepatic impairment is not recommended, as there are no relevant studies of camrelizumab in patients with severe hepatic function abnormal. Currently, there are no study data on camrelizumab in patients with moderate to severe renal impairment; use in patients with moderate or severe renal impairment is not recommended. Patients with mild renal impairment should use camrelizumab cautiously under the guidance of a physician, and if usage is necessary, no dose adjustment is required.

Reactive capillary endothelial proliferation mostly occurs on the surface skin, with a few cases observed in the oral mucosa, nasal mucosa, and eyelid conjunctiva. Reactive capillary endothelial proliferation occurring on the skin initially often presents as bright red pinpoint lesions on the body surface, with a diameter ≤2 mm. With the increase of the number of doses, the lesion range may gradually enlarge, mostly nodular, or patchy, bright red or dark red in color. Clinical symptoms and signs need to be observed, which can be managed according to the following grading criteria and treatment recommendations:

**Table 7. Treatment Recommendations for Reactive Capillary Endothelial Proliferation Related to Camrelizumab**

| **Grading†** | **Clinical Manifestations** | **Treatment Recommendations** |
| --- | --- | --- |
| Grade 1 | Maximum single diameter ≤10 mm, with or without ulceration and hemorrhage | Continue the drug, and the easily rubbed area can be protected with gauze to avoid hemorrhage. The area with ulceration and hemorrhage can be treated with local compression hemostasis. |
| Grade 2 | Maximum single diameter >10 mm, with or without ulceration and hemorrhage | Continue the drug, and the easily rubbed area can be protected with gauze to avoid hemorrhage. The area with ulceration and hemorrhage can be treated with local compression hemostasis, or local treatment measures, such as laser or surgical resection. Avoid infection at the site of ulceration. |
| Grade 3 | Generalized, can be complicated by skin infection, may require hospitalization. | Interrupt the drug, resume the drug after recovery to ≤ Grade 1, and the easily rubbed area can be protected with gauze to avoid hemorrhage. The area with ulceration and hemorrhage can be treated with local compression hemostasis, or local treatment measures, such as laser or surgical resection, and anti-infective treatment should be given for those complicated with infection. |
| Grade 4‡ | Multiple and generalized, life-threatening | Permanent discontinuation |
| Grade 5‡ | Death |  |

† Grading is based on the *CSCO Guidelines for Toxicity Management Related to ICIs 2019*.
‡ No Grade 4 life-threatening or Grade 5 death AEs occurred in the camrelizumab studies.

When the patient develops this adverse reaction, scratching or rubbing should be avoided. Areas prone to friction can be protected with gauze to prevent hemorrhage. Simultaneously, the doctor should be contacted to obtain appropriate management advice. For those with ulcerative hemorrhage, local compression haemostasis can be applied. For recurrent cases, after haemostasis, patients can visit the dermatology department for local treatments such as laser therapy or surgical excision. Patients with local concurrent infection should be given local anti-infective therapy. Reactive capillary endothelial proliferation may occur in tissues other than the skin (including palpebral conjunctiva, medial and external canthus, oral mucosa, throat or other organs), and appropriate medical examinations, such as fecal occult blood, endoscopy, and imaging examinations, should be performed according to self-examination signs and symptoms, if necessary (for details, refer to the "Information Collection and Risk Management Plan for Reactive Capillary Endothelial Proliferation").

## Concomitant and Excluded Medications

### Concomitant Medications

Concomitant medications include any prescription and over-the-counter drugs. All medications used by the patient within 21 days prior to the first dose administration up to the 30-day safety visit should be recorded. Only subsequent anti-tumor therapies will be recorded after the 30-day safety visit. Patients on oral contraceptives and hormone replacement therapy may continue to take the medications. All concomitant medications, including the start and end dates, route of administration, usage, dosage, and indications, must be recorded in the Case Report Form. Patients should, as much as possible, inform and consult the investigators before using any concomitant medications.

### Excluded Medications

Patient cannot receive other anti-tumor therapies at the same time throughout the study, including but not limited to: chemotherapy, radiation therapy, biotherapy, hormonal therapy or any other investigational product therapy, and traditional Chinese medicine with anti-tumor effect cannot be used during the entire period of investigational product therapy. If a patient requires palliative local radiotherapy for symptomatic relief (e.g., local radiotherapy to relieve cancerous bone pain), the patient may restart the investigational product 7 days after the end of local radiotherapy, while the patient must meet the following criteria:

- The patient recovers from radiation therapy-related toxicity to ≤ Grade 2;
- No tumor progression.

### Drug Interaction

In the in vitro liver microsomal incubation studies, the primary Phase I metabolic enzymes mediating the metabolism of surufatinib include CYP3A4/5 and CYP2C8 (dehydrogenation, N-demethylation), CYP2E1 (mono-oxygenation), and FMO. Surufatinib shows no significant reversible inhibition on P450 enzymes. Surufatinib exhibits time-dependent inhibition on CYP3A4/5, with k_inact_ of 0.73 min⁻¹ and KI of 163 μM. Results from induction studies in human primary hepatocytes showed that surufatinib did not have significant induction effects on CYP1A2 at a concentration of 10 μM and on CYP3A4 at a concentration of 2 μM. In vitro permeability and efflux transport studies using Caco-2 cells suggested that surufatinib may be a substrate of the efflux transporter P-gp. Strong inducers, strong inhibitors, or substrates of CYP3A4 should be avoided concomitantly during the study. If the investigator deems it necessary to use them, the potential for reduced efficacy or increased toxicity due to drug interactions should be closely monitored. Appendix 8 provides a list of some strong inhibitors, strong inducers, and substrates of CYP3A4.

## Subject Compliance

It is the investigator's responsibility to ensure that patients comply with the study treatment, which is supervised by the sponsor's representatives through on-site monitoring visits. The investigator should maintain complete and accurate records of drug usage data. Patients' medication administration and actual dosage taken need to be reflected in the source medical records and the CRF. At each treatment visit, the investigator or study staff needs to assess the patient's treatment compliance, making a comprehensive evaluation based on the quantity of medication dispensed at each visit, the amount of medication returned by the patient, and the patient's reported actual consumption, missed doses, overdoses, etc. At the end of the study, patients must return all medication bottles and any remaining capsules.

## Study Procedures

Subjects must read and sign the informed consent form currently approved by the EC prior to initiation of the study. All study steps must be conducted within the time windows specified in the study schedule.

The utmost effort will be made to ensure that the required tests and procedures are carried out according to the protocol. However, unscheduled situations occur from time to time beyond the investigator’s control, making the examinations difficult to perform. In such cases, the investigator must take all necessary measures to ensure the safety and interests of the subjects. When a required test cannot be conducted, the investigator needs to record the reasons. Additionally, the study team should be promptly notified of any unexpected situations in a timely manner.

## Screening

Unless otherwise specified, the following screening procedures must be completed within 21 days prior to starting investigational product therapy; during the screening visit (Day -21 to Day 1):

- Obtaining the signed written informed consent from the subject.
- Collection of medical history and demographic data (specify detailed information, such as body height, weight, etc.);
- Disease treatment history (chemotherapy, radiotherapy...)
- Collection of medication history
- Careful and comprehensive physical examination (in detail): ECOG PS score, body height, weight, vital signs (blood pressure, pulse, etc.), examination of various organs;
- Necessary laboratory tests, collection of blood and urine samples;
- Necessary imaging examinations;
- Evaluation of current concomitant medications and treatments;
- Evaluation of current symptoms/AEs.

## Enrollment

Each subject is evaluated for all eligibility screening criteria. By logging into the IWRS online, the investigator should complete the IWRS worksheet to register screening. The judgment of eligibility criteria during the screening period should be medically reviewed and confirmed by the investigator prior to randomization. If the subject meets all eligibility criteria, they can be randomized, receive a randomization number, and be enrolled in the study. The subject who discontinues the study after signing the informed consent form and before randomization will be considered as a "screening failure". Subjects who previously failed screening may be re-screened once. A new subject number needs to be reassigned through IWRS. Subjects can only be re-screened once.

## Treatment Period

The test group and the control group both have a treatment cycle of 21 days. Unless otherwise specified, all visits during the treatment period should be completed within ±2 days of the scheduled date, and the evaluation times should refer to the study flowchart. The evaluation content includes the assessment and recording of AEs, laboratory tests, electrocardiograms, echocardiograms, HBV DNA or HCV RNA testing (for those who tested positive for HBs Ag, HBe Ag, or HCV Ab during the screening period), etc. This study will conduct tumor assessments according to RECIST version 1.1 standards, with tumor assessments performed every 6 weeks ±2 days after the start of treatment. For patients who have discontinued study treatment before disease progression, tumor assessments should be carried out as planned whenever possible. Unscheduled examinations may be conducted if clinically indicated.

## Follow-up Period

After the patient completes the 30-day safety visit following the end of study treatment, if the patient has unresolved AEs, follow-up will continue until the AEs return to baseline, the AEs stabilize, the patient initiates new anti-tumor therapy, is lost to follow-up, withdraws informed consent, or it is determined that the AEs are unrelated to the study treatment or participation in this study. Newly occurring drug-related SAEs also need to be recorded and reported.

If a patient discontinues study treatment early for reasons other than disease progression, tumor assessments should be conducted per protocol whenever possible before the initiation of other anti-tumor therapies, until the patient experiences disease progression, death, is lost to follow-up, withdraws the informed consent, or the trial ends.

Subjects who discontinue treatment for any reason will undergo survival/post-tumor treatment follow-up every 3 months (±7 days) within one year after the end of treatment (EOT). After one year, survival/post-tumor treatment follow-up will be conducted every 6 months (±7 days), until death, loss to follow-up, withdrawal of consent, refusal to continue to provide information, or the end of the trial as a whole. All surviving subjects will be followed for survival once before the end of the entire study. The investigator should try to avoid loss to follow-up, and if the subject cannot be contacted at the time of survival follow-up, he/she needs to contact at least three times on a different date within each survival visit window. Only after all attempts have been made and the subject still cannot be contacted during all three OS visits can they be recorded as lost to follow-up.

After the entire trial is concluded, no further follow-up will be conducted on the subjects.

## Continuation of Medication after the End of the Study

After the end of the study, if the subjects continue to benefit from the treatment as judged by the investigator, then they will be allowed to receive the investigational product until the treatment discontinuation criteria (disease progression, intolerable toxicity, subject refusal to continue the investigational product, the investigator judged that the subject is no longer suitable to continue the investigational product) are met provided that the investigator has obtained their informed consent for this continued treatment. SAEs will be collected and recorded during and after the last dose according to the protocol.

# Evaluation

## Efficacy Evaluation

Baseline tumor assessment should be completed within 21 days prior to the first dose. CT or MRI of the chest, abdomen and pelvis at baseline is recommended. If there are clinical indications or suspected lesions in other areas, tumor imaging evaluation must also be performed. Baseline and post-treatment response assessments should be performed using the same method and as far as possible by the same investigator (RECIST 1.1 criteria). At screening and subsequent tumor evaluations, all measurable and non-measurable tumor lesions should be recorded and evaluated. Measurable and non-measurable lesions will be evaluated by the investigator per RECIST criteria version 1.1.

Tumor assessments will be performed at baseline and every 6 weeks ± 2 days after start of treatment., until disease progression, withdrawal of informed consent, or death (whichever occurs first). The subjects who discontinue the study treatment for reasons other than disease progression or death should undergo tumor assessments as planned as far as possible until disease progression. Unscheduled tumor assessments may be performed by the investigator as clinically indicated or when disease progression is suspected.

If there are no contraindications, tumor assessment during screening should include enhanced CT scans (preferred) of the head, chest, abdomen, and pelvis; if there are contraindications to contrast agents, MRI may be used. If metastases to bone are known or suspected at baseline, a bone scan examination should be performed; or if bone scan results suggest possible metastases to bone, corresponding bone CT/MRI or X-ray verification is required. Other relevant examinations may also be conducted as clinically indicated.

For each patient, the same tumor examination methods and procedures as at baseline should be used throughout the study period. During subsequent follow-up, bone scan examinations should be repeated only when it is necessary to confirm a complete response or when progression of bone metastasis lesions is suspected. Head CT/MRI may be repeated only when brain metastasis lesions are suspected.

If disease progression is suspected or based on clinical judgment, the investigator may decide to perform unscheduled tumor evaluations at any time. Patients who have discontinued study treatment before disease progression should undergo tumor assessment as originally planned prior to initiating new anti-tumor therapy.

Selection of target lesions should follow the RECIST version 1.1 criteria, that is, under the premise of no more than 2 target lesions per organ and a total of no more than 5 target lesions, as many involved organs as possible should be included. At baseline, the number of target lesions, description of their locations, the maximum diameter of each target lesion (excluding lymph nodes), the minimum diameter of lymph nodes, and the sum of diameters of all target lesions should be recorded.

When the investigator suspects that the subject may have pseudoprogression, PD should be confirmed in principle at least 4 weeks later (it is recommended that the investigator confirm PD according to irRECIST). During the PD confirmation period, the subject should continue to receive investigational product therapy.

## Safety Evaluation

**AE**

The safety of both groups will be comprehensively evaluated based on the incidence and severity of AEs, graded according to NCI CTCAE version 5.0. Safety endpoints include but are not limited to: the overall incidence of AEs; the incidence of Grade 3 or higher AEs; the incidence of drug-related AEs; the incidence of SAEs; the incidence of AEs leading to permanent discontinuation; the incidence of AEs leading to interruption or dose adjustment.

**Vital Signs Assessment**

Vital signs include four parameters: blood pressure, heart rate, respiration, and body temperature. Before measuring blood pressure, patients need to sit quietly for 5 minutes.

**Performance Status Score and Physical Examination**

During the screening period, the performance status score and a comprehensive physical examination should be completed, including weight, head, neck, ears, nose, eyes, throat, cardiovascular, skin and mucous membranes, musculoskeletal, respiratory, gastrointestinal, and nervous system.

Subsequent visits will score the performance status, and only partial physical examinations are required to evaluate changes in abnormal signs relative to baseline, new abnormal signs, and patient-reported symptoms. If applicable, new or worsening abnormal signs should be recorded as AEs.

As a part of tumor evaluation, physical examinations should include enlarged lymph nodes, skin lesions, hepatomegaly, splenomegaly, etc.

Height will be measured at screening only. Body weight will be measured at screening and at subsequent visits.

**Liver Function (Child-Pugh Modified Grading) Score**

Liver function will be scored according to different degrees of serum total bilirubin, serum albumin concentration, prothrombin prolongation time, ascites and hepatic encephalopathy.

**Laboratory Evaluation**

The laboratory of the study site will be used to monitor hematology, clinical chemistry, urinalysis, etc., at screening and at each visit point, including at least the following parameters.

Hematology (expected blood volume 15-20 mL): red blood cell, hemoglobin, neutrophil, platelet count and absolute differential white blood cell;

Coagulation parameters: APTT, PT, INR;

Clinical chemistry: blood urea nitrogen (or urea), creatinine, sodium, potassium, magnesium, chloride, calcium, phosphorus, fasting blood glucose, total bilirubin, ALT, AST, ALP, lactate dehydrogenase, total cholesterol, triglycerides, total protein, albumin, blood uric acid and serum amylase, etc.;

Urinalysis: routine urine (urine protein, occult blood, glucose, ketones) and 24-hour urine protein quantification;

Fecal occult blood test;

Thyroid function test, including FT3, FT4, and TSH;

Hepatitis B and C serology: screening for hepatitis B (hepatitis B surface antigen [HBs Ag], hepatitis B surface antibody, hepatitis B e antigen [HBe Ag], hepatitis B e antibody, hepatitis B core antibody) and hepatitis C antibody (HCV Ab) will be performed at screening. The tests performed before screening period are also acceptable, and it is not necessary to be repeated at screening.

Blood pregnancy test: female subjects of childbearing potential (within 2 years of natural menopause) should have a blood pregnancy test performed at screening and within 30 days after the end of study treatment. A pregnancy test should be performed when the subject is suspected to be pregnant.

Normal ranges for laboratory parameters will be collected for each site prior to study initiation.

**Cardiac Monitoring**

Left ventricular ejection fraction (LVEF) will be evaluated by echocardiography at screening, every 6 weeks ± 2 days after the start of treatment, and within 30 days after the end of treatment; 12-lead electrocardiogram will be performed at screening, within 2 days prior to dosing on Day 1 of each treatment cycle starting from Cycle 2, and within 30 days after the end of treatment.

## European Organisation for Research and Treatment of Cancer (EORTC) Quality of Life Questionnaire

EORTC QLQ-C30 questionnaire: the EORTC QLQ-C30 questionnaire consists of 30 questions of 5 domains of patient functional assessment (physical, emotional, role, cognitive, and social), 3 symptom scales (fatigue; nausea, vomiting, and pain; and general health/quality of life [HRQoL]); and 6 items (dyspnea, insomnia, decreased appetite, constipation, diarrhea, and financial difficulties) describing the patient's status during a recall period termed "recent week".

EORTCQLQ-C30 is self-administered and completed by patients at the study site. The questionnaire should be completed within 7 days before the patient's study treatment, at the Day 1 visit of each treatment cycle starting from Cycle 2, and should be completed before conducting various clinical examinations and clinical evaluations, before being informed of any new disease information, and before initiating any new treatments, until the end of treatment.

# AE Report

## AE

AEs will be collected from the time when the subject signs the informed consent form until the end of the safety follow-up period (30 days after the last dose).

### Definition of AE

An AE is defined as any untoward medical event that occurs after receiving a drug or treatment or any deterioration of a disease or symptom that existed before receiving the investigational product or treatment (excluding the disease studied in this study) in a patient or a clinical study subject, whether or not considered related to the investigational product or treatment. AEs can be any unfavorable and unexpected symptoms, signs, laboratory abnormalities, or diseases, including the following situations:

1) Worsening of pre-existing (before enrollment) medical conditions/diseases (including worsening of symptoms, signs, laboratory abnormalities);

2) Any new AEs: any new adverse medical conditions (including symptoms, signs, newly diagnosed diseases);

3) Abnormal and clinically significant laboratory values or results.

The investigator should record in detail any AEs that occur in the subjects, including: the name of the AE and a description of all related symptoms, the time of onset, severity, relationship to the investigational product, duration, measures taken, final results and outcome.

### Criteria for AE Severity

AEs will be graded per NCI-CTCAE v5.0. The following criteria should be used for AEs not listed in the NCI CTCAE v 5.0:

**Table 8 Criteria for AE Severity**

| **Grade** | **Clinical Description of Severity** |
| --- | --- |
| 1 | Mild; with no symptoms or minor clinical symptoms; only clinical or laboratory abnormalities; no treatment required. |
| 2 | Moderate; requiring minor, local, or non-invasive treatment; limitations in age-appropriate instrumental activities of daily living (ADL); instrumental ADL refer to cooking, shopping, making phone calls, and counting money, etc. |
| 3 | Severe or medically significant but not immediately life-threatening; hospitalization or prolongation of hospitalization indicated; disabling; limiting self-care ADL. Self-care ADL refers to bathing, dressing and undressing, feeding self, using the toilet, taking medicine, etc., and not bedridden. |
| 4 | Life-threatening; urgent intervention indicated. |
| 5 | Resulting in death |

### Determination of Causality between AE and Investigational Product

AEs will be collected from signing the informed consent form until the end of the safety follow-up period, regardless of whether the event is related to the investigational product, whether or not the subject is assigned to the investigational product group, or even whether or not the drug is used. Any discomfort reactions reported by the subjects during the treatment period or any abnormal changes in objective laboratory indicators should be accurately recorded, along with the severity, duration, management measures, and outcome of the AE. The investigator should comprehensively assess the relationship between the AE and the investigational product, for example. whether there is a reasonable temporal sequence between the occurrence of the AE and drug administration, the characteristics as well as toxicological and pharmacological effects of the investigational product, whether the subject is using other concomitant medications, the subject’s underlying diseases, medical history, family history, provocation and rechallenge reactions, etc. The possible causality between the AE and the investigational product should be assessed as "definitely related, possibly related, unlikely related, not related, and unable to determine" using a five-level classification method.

## SAE

### Definition of SAE

SAEs refer to adverse medical events occurring during the course of a clinical study that require hospitalization or prolongation of hospitalization, result in disability, affect work ability, endanger life or death, or lead to congenital malformations. SAEs include the following medical events:

- Events leading to death;

• Life-threatening events (defined as a situation where the subject is at immediate risk of death when the event occurs);

• Events requiring hospitalization or prolongation of hospitalization;

• Events that may lead to permanent or serious disability/functional impairment/impact on work capacity;

• Congenital anomalies or birth defects;

• Other medically significant events (defined as events that harm the subject or require intervention to prevent any of the above situations from occurring).

### Hospitalisation

AEs in clinical studies that lead to hospitalization (even if less than 24 hours) or prolongation of existing hospitalization should be considered as SAEs.

However, hospitalization or prolongation of hospitalization for the following reasons should not be reported as an SAE:

- Rehabilitation facility
- Sanatorium
- Routine emergency room treatment (less than 24 hours)
- Day surgery (such as outpatient/day/ambulatory surgery)
- Social reasons (medical insurance reimbursement, etc.)

Any hospitalization or prolongation of hospitalization unrelated to the worsening of AEs is not considered as an SAE. For example:

- Hospitalization due to a pre-existing disease without a new AE or exacerbation of the pre-existing disease (such as hospitalization to check for laboratory abnormalities that occurred prior to the study and are still present);
- Hospitalization due to administrative reasons (e.g., annual routine physical examination);
- On-study hospitalization as specified in the protocol (e.g., protocol-specified procedures);
- Elective hospitalization for any cause other than the worsening of AEs (e.g., elective surgery);
- Pre-scheduled treatment or surgery, which should be documented in the study protocol and/or the subject’s baseline data;
- Hospitalization for blood product use only.

Any invasive procedures (such as surgery) and non-invasive procedures for diagnosis or treatment should not be reported as an AE. However, if the medical condition leading to the procedure meets the definition of an AE, it should be reported as an AE. For example, acute appendicitis occurring during the AE reporting period should be reported as an AE, while the appendectomy performed as a result should be documented as the treatment of the AE.

### Disease Progression and Death

Disease progression is defined as the worsening of the subject's condition caused by the indication of the study. It includes radiological progression and the progression of clinical symptoms and signs. A new lesion of the primary tumor or progression of an existing lesion will be considered disease progression. Events that are life-threatening or that require hospitalization or prolongation of hospitalization, or that result in permanent or significant disability/incapacity/impairment of work or congenital anomaly or birth defect will not be considered SAEs for reporting if they are due to symptoms and signs of disease progression. If there is any uncertainty as to whether an SAE is caused by disease progression, it should be reported as an SAE.

In the study population of this study, “disease progression” is an expected occurrence and should not be reported as an AE term. When disease progression occurs, the event used to confirm disease progression should be reported as an AE. For example, if a subject has epilepsy that is determined to be related to brain metastasis, the AE term should be recorded as “epilepsy”, rather than “disease progression” or “brain metastasis”.

If a subject dies during the study, regardless of whether or not he/she has received new anti-tumor therapy, it must be reported as an SAE (refer to Section 7.4, Table 9 Principles for AE/SAE Collection and Follow-up Period). Deaths assessed by the investigator as possibly due to symptoms and signs of disease progression should be recorded on the eCRF and will not be reported as SAEs. However, the investigator should record the death in the CRF Death Report Form and promptly inform the sponsor. The term "death" should not be used as an AE or SAE term, but rather as the outcome of an event. The event that results in or leads to death should be recorded as an AE or SAE. If the cause of death is unknown and cannot be determined at the time of reporting, the AE or SAE term should be recorded as "death of unknown cause".

### Other Anti-tumor Therapies

SAEs will be recorded from the time the subject signs the informed consent form until the end of the safety follow-up period (30 days after the last dose of investigational product). If a subject starts other anti-tumor therapy before the end of the safety follow-up period, for SAEs that are not fatal, unless suspected to be related to the investigational product, the reporting period ends until the start of new anti-tumor therapy. Deaths occurring during the safety follow-up period must be reported as SAEs, regardless of whether or not there are other treatments.

For certain investigational products, such as camrelizumab, investigational product-related SAEs also need to be collected due to the possibility of a delayed immune response.

### SAE Reporting System

SAEs should be collected from the time the subject signs the informed consent form until the end of the safety follow-up period. In the event of an SAE, whether the initial report or the follow-up report, the investigator must immediately fill in the Serious Adverse Event Report Form, sign and date, then send it to the pharmacovigilance department of the sponsor by telephone (4006586360), email or fax (021-20673186) within 24 h of learning of the SAE, and report to the National Bureau, Health Commission, EC and the Office of Clinical Trial Facility (if applicable) according to the corresponding requirements of the study site. The initial report should include, to the extent possible, the following: source of report, subject general information, investigational product name, SAE name, severity, relationship to the investigational product, treatment, and outcome of the event. The initial SAE report, follow-up report and final report should indicate the unique identification code of the subject in the trial, rather than the identification information such as his/her real name, ID card number or address.

After the investigator becomes aware of the SAE, the SAE should be analyzed, assessed and judged promptly. If it is assessed as an SUSAR, it will be reported to relevant departments such as the National Medical Products Administration, National Health Commission, EC and office of the clinical study institution (if applicable) in accordance with the current Standards and Procedures for Expedited Reporting of Safety Data During Drug Clinical Studies and relevant requirements of the study site. SAEs that occur after the safety follow-up period and are suspected to be related to the investigational product should be collected.

For SAEs, the symptoms, severity, relationship to the investigational product, time of occurrence, time of management, measures taken, follow-up time and manner, and outcome should be recorded in detail. If the investigator thinks that an SAE is unrelated to the investigational product but potentially related to the study conditions (e.g., discontinuation of the pre-existing treatment, or complications during the trial), details should be described in the narrative section of the SAE report. If the severity of an ongoing SAE or its relationship to the investigational product is changed, a follow-up report should be submitted immediately. If the investigator believes that previously reported SAE information was misreported, then correction, revocation or downgrading instructions may be made through a follow-up report, which should be submitted in accordance with the SAE reporting procedures.

The sponsor of this project receives the SAE reports at safety@hmplglobal.com.

## Pregnancy

If a female subject becomes pregnant during the clinical study, the subject is discharged from the group; if the partner of a male subject becomes pregnant during the clinical study, the subject will continue the clinical study. The investigator should fill out the "Hutchison Whampoa Clinical Study Pregnancy Report/Follow-up Form" and report to the sponsor within 24 hours of becoming aware of the pregnancy event, as well as report to the EC in a timely manner.

The investigator should follow up the pregnancy outcome until 1 month after delivery, and report to the sponsor.

Pregnancy outcomes stillbirth, spontaneous abortion or fetal malformation will be considered as SAEs, and should be reported within specified time limit for SAE reporting.

If a subject also develops an SAE during pregnancy, the investigator should also fill out the "Serious Adverse Event Report Form", and report according to SAE reporting procedures.

## Follow-up for AEs/SAEs

All AE/SAEs should be followed up until the end of the safety follow-up period or they have disappeared, recovered to baseline level or ≤ Grade 1, reached a stable state, or been reasonably explained (such as lost to follow-up, death).

The investigator should inquire about the AE/SAEs that occurred after the last visit at each visit, and provide follow-up information in a timely manner according to the request of the sponsor. During the end-of-study period, the collection and follow-up period of AEs/SAEs occurring after the last dose of subjects can be found in the following table:

Table 9 Principles of AE/SAE Collection and Follow-up Period

| **Classification** | **Collection/Recording Requirements** | **Follow-up Requirements** |
| --- | --- | --- |
| AEs without drug-related | Until the end of the safety follow-up Period or start of new anti-tumor therapy, whichever comes first | Until the end of the safety follow-up period |
| AEs with drug-related | Until the end of the safety follow-up period | Follow-up until disappearance, response, or return to baseline levels, or ≤ Grade 1, or achieving a stable state, or obtaining a reasonable explanation (e.g., lost to follow-up, death). |
| SAEs and SIEs without drug-related | Until the end of the safety follow-up Period or start of new anti-tumor therapy, whichever comes first | Until the end of the safety follow-up period |
| SAEs and SIEs with drug-related | Indefinite | Follow-up until disappearance, response, or return to baseline levels, or ≤ Grade 1, or achieving a stable state, or obtaining a reasonable explanation (e.g., lost to follow-up, death). |
| AE: adverse event; SAE: serious adverse event; SIE: adverse event of special interest | | |

# Statistical Methods and Analysis

## Analysis Population

Intent-to-Treat Population (ITT Population): all randomized patients will be included for analysis according to the intention-to-treat principle. The ITT population is used for analyzing all efficacy endpoints.

Per-Protocol Analysis Population (PP Population): patients in the ITT Population who had no major protocol deviations affecting the efficacy assessment. The PP population is used for sensitivity analyses of OS and PFS.

Safety Population (Safety Set, SS Population): includes all randomized patients who received at least one dose of investigational product. The SS population is used for analyzing safety endpoints.

## Efficacy Analysis

### Primary Efficacy Analysis

**Objective Response Rate (ORR)**

Objective response rate is defined as the proportion of patients with a best overall assessment of complete response or partial response. Clopper-pearson method is used to calculate the 95% confidence intervals of the rates in each group. Depending on the characteristics of the data, rates between groups are compared using CMH test or chi-square test or Fisher exact probability method.

### Secondary Efficacy Analysis

**Progression-free Survival (PFS)**

PFS is defined as the time (in days) from randomization to disease progression or death. Overall survival is compared between the test group and the control group using a stratified log-rank test. The Kaplan-Meier method will be used to estimate the median survival time of both treatment groups, and Kaplan-Meier curves will be plotted to provide a visually intuitive description of the differences between treatment groups.

**Overall Survival (OS)**

Overall survival (OS) is defined as the time from randomization to death from any cause. For patients who have not reported death at the time of analysis, the date of their last known survival will be taken as the censoring date. The analysis method is the same as for PFS.

**Disease Control Rate (DCR)**

Disease control rate is defined as the proportion of patients with a best overall assessment of complete response, partial response, or stable disease. For patients assessed as having stable disease, at least one lesion assessment meeting the SD criteria is required at least 6 weeks after starting medication. The analysis method is the same as that for ORR.

The comparison of ORR, PFS, OS, and DCR between treatment arms will be based on ITT.

### Quality of Life Scores and Tumor Biomarkers

Quality of life scores and tumor markers will be summarized mainly using descriptive statistical methods.

## Safety Analysis

Safety will be evaluated by summaries of AEs, changes in laboratory results, and changes in vital signs.

All AEs will be graded according to NCI CTCAE (version 5.0). AEs will be coded using the Medical Dictionary for Regulatory Activities (MedDRA). The number and incidence of AEs will be summarized by human system organ class (SOC) with corresponding terms. All SAEs (including deaths) and AEs leading to dose reduction, dose interruption, and discontinuation of investigational product should be tabulated and summarized separately.

All laboratory assessments will be summarized and tabulated. Changes in laboratory test results will be graded according to NCI CTCAE (version 5.0), and comparison of baseline and post-treatment maximum toxicity will be presented in shift tables. Patients with laboratory abnormalities of CTCAE grade 3 or 4 will be tabulated separately.

Changes in physical examinations, vital signs, and ECOG Performance Status scores will be compared with baseline and will be analyzed descriptively.

Patients exhibiting abnormalities in electrocardiogram and echocardiogram will be tabulated. Shift tables will be used to summarize changes from baseline to abnormality for each treatment group.

## Sample Size Determination

According to literature results, the rate in the test group is 0.49, and the rate in the control group is 0.23. With a superiority margin of 0, a one-sided alpha of 0.05, beta of 0.2, and a sample size ratio of 1 between the two groups (test group: control group), the calculated sample sizes are 40 cases in the test group and 40 cases in the control group, totaling 80 cases. Considering a 10% dropout rate, the total required sample size is 90 cases.

# Data Management Methods

## Data Recording

Electronic data capture (EDC) will be used for the collection and management of clinical data in this study.

### Completion of Original Medical Records

The original medical records should be kept in their entirety as the original documents for the clinical study. The investigator is responsible for filling out and keeping the original medical records. Before each completion, the subject information on the cover of the medical records should be checked, and medical records should be completed in neat and legible handwriting to facilitate data reconciliation with the eCRF by the CRA of the sponsor during each monitoring.

### eCRF Completion

Data in the eCRF should come from original medical records such as study medical records and laboratory test reports and should be consistent with the original documents. Any observations and inspection results in the study should be recorded in the eCRF in a timely, accurate, complete, standardized, and truthful manner.

When making data corrections in the eCRF, the reason for the data modification should be filled in according to the system prompts.

### eCRF Review

The investigator should complete, save, and submit the eCRF promptly after each visit of each subject. The system's logic check program will check the integrity and logic of the data entered into the EDC system and raise queries for problematic data. The investigator or data entry personnel are allowed to modify or explain the problematic data. Queries can be raised multiple times until the problematic data is resolved. The CRA, data manager, and medical reviewer may also review the eCRF data and raise queries for any problematic data. The investigator should respond promptly to queries from the system and data reviewer. After data cleaning is completed, the investigator electronically signs the completed eCRFs.

## Data Management

### Establishment of EDC Database

Data managers will establish a study data collection system and database according to the study protocol and provide it online before subject enrollment. All EDC users need to complete relevant training, and fill out training record and account application form in order to obtain the corresponding account to log in to the system.

### Data Review and Database Lock

Prior to database lock, the project team needs to summarize all protocol deviations that occur during the conduct of the study and hold a data review meeting. Decisions made at the data review meeting must be documented.

After all data have been reviewed and approved, the database will be locked upon confirmation by the investigator, sponsor and statistician. After locking, the data files cannot be altered. The locked data should be properly preserved for future reference.

### Data Archiving

Upon completion of the study, eCRFs in PDF format will be generated in the EDC system, which will be stored on read-only CDs and submitted to the sponsor and study site for filing and future audits.

Upon completion of the study, eCRFs in PDF format will be generated in the EDC system, which will be stored on read-only CDs and submitted to the sponsor and study site for filing and future audits. After the investigational product is approved for marketing, or even if the clinical study is discontinued early, the sponsor should keep the clinical trial data for at least 10 years. All data of this clinical study belong to the principal investigator and his/her institution. Except as required by the National Medical Products Administration, no one may provide it to a third party in any form without the written consent of the principal investigator (principal study unit).

# Source Data and Source Documents

In accordance with ICH E6, relevant regulations, and the study institution's requirements for the protection of subject personal information, each study site must properly keep records related to this study. As part of the sponsor's participation in the study, study sites should allow sponsor-authorized representatives and regulatory agencies to inspect (and, if legally permitted, copy) clinical records for quality review, auditing, safety assessment, research progress evaluation, and data validity assessment.

Source data represents all information essential for the reconstruction and evaluation of clinical studies, and it is the original record of clinical findings, observations, or other activities. Examples of these original documents and data records include, but are not limited to: hospital records, laboratory records, memos, subject diaries, pharmacy dispensing records, recorded consultations, data from automated instruments, copies or transcriptions that have been verified and deemed to be accurate and complete, microfiche, photographic plate, microfilm strip or disk, X-ray film, and files and records of subjects stored in participating pharmacies, laboratories, and medical technology departments.

# Quality Assurance and Quality Control

In order to ensure the quality of the trial, the sponsor and the investigator jointly discuss and develop a clinical study plan before the official start of the trial, and confirm whether the relevant study personnel involved in the trial have received appropriate GCP training.

Study medication must be managed by each site in accordance with SOPs, including receipt, storage, distribution, recovery, and destruction.

According to GCP guidelines, necessary measures should be taken during the design and implementation phases of the study to ensure that the data collected is accurate, consistent, complete, and reliable. All observation results and abnormal findings in the clinical study should be carefully verified and documented in a timely manner to ensure the reliability of the data. Instruments, equipment, reagents, and standards used in various examinations in clinical study should follow strict quality standards and function normally.

Information required by the protocol should be entered into eCRF by the investigator. The monitor should verify the integrity and accuracy of the entered information, and instruct the study site personnel to make necessary modifications and additions.

Drug regulatory authority, EC, and monitors and/or auditors of the sponsor may conduct systematic checks on study-related activities and documents to assess whether the study is performed in accordance with the protocol, SOPs and applicable laws and regulations, as well as whether the study data is recorded timely, truthfully, accurately and completely. Audits should be conducted by personnel not directly involved in the clinical study.

# Ethics

## Ethical Codes

This clinical trial must comply with the International Ethical Guidelines for Biomedical Research in Humans (CIOMS, 2002), the current ICH-GCP, the Declaration of Helsinki, and relevant regulations. Before the start of the study, the EC should be provided with the protocol, protocol amendments, ICF and other relevant documents, such as recruitment advertisements. This study must be approved by relevant regulatory departments and the Hospital EC before it can be conducted.

Neither party may unilaterally modify this study protocol without the agreement of both the sponsor and the investigator. The modified protocol must be approved by the EC before implementation. If, in order to eliminate obvious direct harm to the subjects, the investigator must enter a process that deviates from the protocol, they must immediately notify the EC and the sponsor in writing after the deviation is implemented, and explain and record any deviations made from the protocol.

During the clinical study, any modifications made to the protocol should be submitted to the EC, and, if necessary, other study documents should be revised accordingly at the same time, and submitted for approval according to the requirement of the EC. The investigator is responsible for submitting interim reports to the EC as required and must notify the EC when the study has ended.

## Independent EC

The protocol, informed consent form, recruitment materials, and all materials of subjects will be submitted to the EC for review and approval. Subject enrollment can be started only after the protocol and ICF are approved. Any amendments to the protocol must be reviewed and approved by the EC before implementation. All revisions to the informed consent form must also be approved by the EC, and it is up to the EC to decide whether subjects who have already signed previous versions of the informed consent form need to re-sign the new version.

## Informed Consent

### Informed Consent Form and Other Written Information Required by Subjects

The informed consent form provides a detailed description of the study treatment and procedures, and adequately explains the risks involved in the study. A written informed consent must be obtained before any study-related procedures are conducted on the subjects. The following informed consent materials will be submitted with the protocol.

### Informed Consent Process and Records

Informed consent process should begin before subjects agree to participate in the clinical study and continue throughout the entire process of the clinical study. The risks and potential benefits of participating in the study will be discussed in detail and thoroughly with subjects or their guardian. The subject will be asked to review the informed consent form approved by the EC. The investigator will explain the clinical study to the subjects and answer any questions they may have. Subjects can only begin participating in the study after signing the informed consent form. During the entire process of the clinical study, subjects may withdraw consent at any time. A copy of the informed consent form will be retained by the subject. Even if patients decline to participate in the study, their rights will be fully protected, and the quality of their medical care will not be affected in any way.

## Confidentiality of Subject Information

The confidentiality of subject information is strictly kept by the investigator, study participants, the sponsor and its agent. Confidentiality also encompasses biological samples and genetic tests, in addition to the subject's clinical information. Therefore, the study protocol, documents, data, and all other information generated will be kept strictly confidential. No relevant study or data information should be disclosed to any unauthorized third party without the prior written approval of the sponsor.

Other authorized representatives of the sponsor, ECs, and regulatory authorities may inspect all documents and records that the investigator is required to maintain. These include, but are not limited to, medical records and subject treatment records. The study site should allow access to these records.

The contact information of subjects will be securely stored at each study site and used internally only during the study process. At the end of the study, all records will continue to be stored securely according to the time limits specified by the local EC and regulations.

### Study Use of Samples, Specimens, or Data

- Planned Use: The samples and data collected according to this protocol will be used for the analysis and data publication of this study and will not be used for any unrelated purposes.
- Storage: When storing samples and data, they will be numbered by the study. Data in computers will also be password protected. Only study personnel have the right to access these samples and data.

# Publication of Study Results

## Utilization of Study Data

All the study specific data concerning surufatinib, such as patent application, dosage form, manufacturing process, and basic study data, should be regarded as confidential information as long as these data has not been published.

Study data obtained in this study will also be considered confidential. The sponsor and investigator shall make it publicly available to other clinical study personnel, NMPA, or other government agencies as appropriate. To ensure the integrity of the clinical data analysis, the study personnel are obliged to provide the complete study results and data to the sponsor.

The investigator must ensure that the privacy of the subjects is not disclosed to any unauthorized third party. The names of the patients should not be included in submitted CRF and other documents, but only identified with codes. The investigator may retain an enrollment form containing the patient’s signature code, name, and address. Informed consent forms and other documents should be strictly confidential, and not be submitted to sponsor.

## Publication

The results of this study may be published in core journals; the principal investigators who have made important contributions to the conduct and management of the study and staff members who have contributed substantially to the design, interpretation, or analysis of the study (e.g., staff or consultants of sponsor) may be listed.

The investigators must obtain the sponsor's consent before submitting academic articles or abstracts. The study personnel have the right to publish the study results provided that they shall comply with requirements for the protection of confidential information.

The intellectual property rights of confidential materials are common to the sponsor and the research initiating institution, may not be disclosed to others without the written consent of the sponsor and the research initiating institution, and may not be used for other purposes other than this study.

# References

1. GLOBOCAN 2020

2. Zeng H, Chen W, Zheng R, et al. Changing cancer survival in China during 2003-15: a pooled analysis of 17 population-based cancer registries. Lancet Glob Health. 2018 May;6(5):e555-e567.

3. Rahib L, Smith BD, Aizenberg R, Rosenzweig AB, Fleshman JM, Matrisian LM. Projecting cancer incidence and deaths to 2030: the unexpected burden of thyroid, liver, and pancreas cancers in the United States. Cancer Res. 2014 Jun 1;74(11):2913-21.

4. Von Hoff DD, Ervin T, Arena FP, et al. Increased survival in pancreatic cancer with nab-paclitaxel plus gemcitabine. N Engl J Med. 2013 Oct 31;369(18):1691-703.

5. Conroy T, Desseigne F, Ychou M, et al. FOLFIRINOX versus gemcitabine for metastatic pancreatic cancer. N Engl J Med. 2011 May 12;364(19):1817-25.

6. Chiorean EG, Cheung WY, Giordano G, et al. Real-world comparative effectiveness of nab-paclitaxel plus gemcitabine versus FOLFIRINOX in advanced pancreatic cancer: a systematic review. Ther Adv Med Oncol. 2019 May 19;11:1758835919850367.

7. Kang J, Hwang I, Yoo C, et al. Nab-paclitaxel plus gemcitabine versus FOLFIRINOX as the first-line chemotherapy for patients with metastatic pancreatic cancer: retrospective analysis. Invest New Drugs. 2018 Aug;36(4):732-741.

8. Portal A, Pernot S, Tougeron D,et al. Nab-paclitaxel plus gemcitabine for metastatic pancreatic adenocarcinoma after Folfirinox failure: an AGEO prospective multicentre cohort. Br J Cancer. 2015 Sep 29;113(7):989-95.

9. J Clin Oncol 38, 2020 (suppl 4; abstr 717)

10. Cui H, Guan J, Deng G, et al. A Chinese Retrospective Multicenter Study of First-Line Chemotherapy for Advanced Pancreatic Cancer. Med Sci Monit. 2020 Oct 26;26:e927654.

11. Henriksen A, Dyhl-Polk A, Chen I, et al. Checkpoint inhibitors in pancreatic cancer[J]. Cancer treatment reviews, 2019, 78: 17-30.

12. Kamath SD, Kalyan A, Kircher S, et al. Ipilimumab and Gemcitabine for Advanced Pancreatic Cancer: A Phase Ib Study. Oncologist. 2020 May;25(5):e808-e815.

13. Aglietta M, Barone C, Sawyer MB, et al F. A phase I dose escalation trial of tremelimumab (CP-675,206) in combination with gemcitabine in chemotherapy-naive patients with metastatic pancreatic cancer. Ann Oncol. 2014 Sep;25(9):1750-1755.

14. Weiss GJ, Blaydorn L, Beck J, et al: Phase Ib/II study of gemcitabine, nab-paclitaxel, and pembrolizumab in metastatic pancreatic adenocarcinoma. Invest New Drugs. 2019 Aug;37(4):797.

15. Wainberg ZA, Hochster HS, Kim EJ, et al. Open-label, Phase I Study of Nivolumab Combined with nab-Paclitaxel Plus Gemcitabine in Advanced Pancreatic Cancer. Clin Cancer Res. 2020 Sep 15;26(18):4814-4822.

16. The Canadian Cancer Trials Group PA. 7 trial: Results of a randomized phase II study of gemcitabine (GEM) and nab-paclitaxel (Nab-P) vs GEM, nab-P, durvalumab (D) and tremelimumab (T) as first line therapy in metastatic pancreatic ductal adenocarcinoma (mPDAC). 2020 ESMO. Abs #LBA65.

17. Yang Haiyan, Cui Jiujie, Wang Liwei et al. Prospective Single-arm Exploratory Clinical Study on Efficacy and Safety of SHR-1210 in Combination with Nab-paclitaxel and Gemcitabine as First-line Treatment for Pancreatic Carcinoma Metastatic. 2020 CSCO Paper ID:7698.

18. ASCO 2020 Abstract #9563

19. Momeny M, Alishahi Z, Eyvani H, et al. Anti-tumor activity of cediranib, a pan-vascular endothelial growth factor receptor inhibitor, in pancreatic ductal adenocarcinoma cells. Cell Oncol (Dordr). 2020 Feb;43(1):81-93.

20. Candido JB, Morton JP, Bailey P, et al. CSF1R+ Macrophages Sustain Pancreatic Tumor Growth through T Cell Suppression and Maintenance of Key Gene Programs that Define the Squamous Subtype. Cell Rep. 2018 May 1;23(5):1448-1460.

# Appendix

**Appendix 1: Guideline on Potential Interactions of Surufatinib with Concomitant Medications**

Currently, there is no data indicating pharmacokinetic interactions between surufatinib and certain concomitant medications.

In the in vitro liver microsomal incubation studies, the primary Phase I metabolic enzymes mediating the metabolism of surufatinib include CYP3A4/5 and CYP2C8 (dehydrogenation, N-demethylation), CYP2E1 (mono-oxygenation), and FMO. Surufatinib has no significant inhibitory effect on P450 enzymes. Results from induction studies in human primary hepatocytes showed that surufatinib did not have significant induction effects on CYP1A2 at a concentration of 10 μM and on CYP3A4 at a concentration of 2 μM. In vitro permeability and efflux transport studies using Caco-2 cells suggested that surufatinib may be a substrate of the efflux transporter P-gp.

According to in vitro tests, surufatinib shows time-dependent inhibition of the metabolic enzymes CYP3A4/5, with k_inact_ of 0.73 min^-1^ and KI of 163 μM. Tables 1-3 in this Appendix provide a series of strong CYP3A4 inducers, strong inhibitors, and substrates. Patients who have received Hypericum perforatum therapy within 3 weeks prior to receiving the first dose of investigational product therapy or have received other strong CYP3A4 inducers or strong inhibitors within 2 weeks cannot participate in this study.

Concomitant use of strong inducers, strong inhibitors, or substrates of CYP3A4 should be avoided during the trial unless deemed necessary by the investigator, but in such cases, patients must be closely monitored for decreased efficacy or increased toxicity of the investigational product due to drug interactions.

These listings are limited, and similar restrictions should be applied to other drugs known to have strong modulation of CYP3A4 activity, or drugs that are primarily metabolized by CYP3A4. If necessary, complete prescribing information for all combination medications should be consulted before coadministration with surufatinib.

**Table 1 Strong Inhibitors of CYP3A4 Enzyme**

| Strong CYP3A4 inhibitors that may increase surufatinib exposure |
| --- |
| Boceprevir  Clarithromycin  Conivaptan  Elvitegravir/Ritonavir  Fluconazole  Grapefruit juice^ab^  Indinavir  Itraconazole  Ketoconazole  Lopinavir/Ritonavir  Mibefradil  Nelfinavir  Posaconazole  Ritonavir  Saquinavir  Telaprevir  Telithromycin  Tipranavir  Ritonavir  Troleandomycin  Voriconazole |

RIT = Ritonavir.Ritonavir

a High concentrations of grapefruit juice

b During the study, subjects should not consume large amounts of grapefruit and lime (and other products containing such fruits, e.g., grapefruit juice or marmalade), e.g. no more than a small glass of grapefruit juice (120 mL) or half a grapefruit or 1 to 2 teaspoons (15 g) of marmalade daily.

**Table 2 Strong Inducers of CYP3A4 Enzyme**

| Strong inducers of CYP3A4 enzyme |
| --- |
| Avasimibe  Carbamazepine  Enzalutamide  Mitotane  Phenobarbital  Phenytoin  Rifabutin  Rifampicin  Hypericum perforatum  Enzalutamide |

**Table 3 Substrates of CYP3A4 Enzyme**

| Substrates of CYP3A4 enzyme |
| --- |
| Alfentanil  Aprepitant  Budesonide  Buspirone  Conivaptan  Darifenacin  Darunavir  Dasatinib  Dronedarone  Eletriptan  Eplerenone  Everolimus  Felodipine  Indinavir  Fluticasone  Lopinavir  Lovastatin  Lurasidone  Maraviroc  Midazolam  Nisoldipine  Quetiapine  Saquinavir  Sildenafil  Simvastatin  Sirolimus  Tolvaptan  Tipranavir  Triazolam  Ticagrelor  Vardenafil  Astemizole  Cisapride  Cyclosporine  Dihydroergotamine  Ergotamine  Fentanyl  Pimozide  Quinidine  Tacrolimus  Terfenadine |

**Appendix 2 Traditional Chinese Medicines Prohibited During the Study**

| Prohibited traditional Chinese medicines during the study include, but are not limited to: |
| --- |
| Huatan Huisheng Tablets  Yadanziyou Soft Capsules  Zhemu Syrup  Cantharidin  Cinobufotalin  Chansu  Kang'ai Injection  Kanglaite  Zhongjiefeng Injection  Aidi Injection  Aweihuapi Plaster  Kangaiping Pills  Fukang Capsules  Xiaoaiping  Ping Xiao Capsules  Ping Xiao Tablets  Shendan Sanjie Capsules  Ankangxin Capsules  Bosheng Aining  Zedoary Turmeric Oil and Glucose Injection  Kang Lixin Capsules  Cidan Capsules |
