# Supplementary material for: First-line treatment with chemotherapy, surufatinib (an angio-immuno kinase inhibitor), and camrelizumab (an anti-PD-1 antibody) for locally advanced or metastatic pancreatic ductal adenocarcinoma: a phase Ib/II randomized study
Source: Signal Transduct Target Ther. 2025 Oct 13;10:339. doi: 10.1038/s41392-025-02441-2 (PMC12515981; doi:10.1038/s41392-025-02441-2)
Supplement: Supplementary file 4 — Supplementary information [file 41392_2025_2441_MOESM4_ESM.docx]

A Randomized, Controlled Phase Ib/II Study of Surufatinib Combined with Camrelizumab and the AS Regimen as First-Line Treatment for Advanced Pancreatic Carcinoma Metastatic

**Statistical Analysis Plan**

**（PHASE II）**

| Clinical Trial Institution | Chinese PLA General Hospital |
| --- | --- |
| Principal Investigator: | Guanghai Dai / Quanli Han |
| Version | V1 |
| Version Date | October 17, 2024 |

**目录**

[List of abbreviations 3](#_Toc202947100)

[Preface 4](#_Toc202947101)

[1 Study objective and Basic information 4](#_Toc202947102)

[1.1 Study objectives 4](#_Toc202947103)

[1.2 Investigational medicinal products 4](#_Toc202947104)

[1.3 Study Design 5](#_Toc202947105)

[2 Endpoint Indicators 6](#_Toc202947106)

[2.1 Primary Study Endpoints 6](#_Toc202947107)

[2.2 Secondary Study Endpoints 6](#_Toc202947108)

[3 Sample Size Determination 6](#_Toc202947109)

[4 Study population 6](#_Toc202947110)

[5 Statistical Analysis 7](#_Toc202947111)

[5.1 Statistical hypothesis and multiplicity considerations 7](#_Toc202947112)

[5.1.1 Statistical hypothesis 7](#_Toc202947113)

[5.2 Statistical analysis software 7](#_Toc202947114)

[5.3 General statistical principles 7](#_Toc202947115)

[5.3.1 Descriptive statistics 7](#_Toc202947116)

[5.3.2 Data Rounding Rules 7](#_Toc202947117)

[5.3.3 General Methods of Statistical Analysis 7](#_Toc202947118)

[5.4 Handling of Missing Data 8](#_Toc202947119)

[5.4.1 Handling of missing values 8](#_Toc202947120)

[5.5 Statistical analysis content and methods 8](#_Toc202947121)

[5.5.1 Subject Disposition 8](#_Toc202947122)

[5.5.2 EFFICACY ANALYSES 8](#_Toc202947123)

[5.5.3 Safety Analysis 9](#_Toc202947124)

[6 Discussion of Statistical Results 10](#_Toc202947125)

[7 Amendment of statistical content from the protocol 10](#_Toc202947126)

[8 Appendix 10](#_Toc202947127)

[9 Reference 10](#_Toc202947128)

**List of abbreviations**

| **Abbreviation** | **Full Name** |
| --- | --- |
| CI | Confidence Interval |
| CMH | Cochran-Mantel-Haenszel |
| Fisher | Fisher's exact test |
| ITT | Intention to treat |
| Max | Maximum |
| Mean | Arithmetic mean |
| Median | Median |
| MedDRA | Medical Dictionary for Regulatory Activities |
| Min | Minimum |
| OR | Odds ratio |
| PPS | Per-protocol set |
| SD | Standard Deviation |
| SS | Safety Analysis Set |

**Preface**

The Statistical Analysis Plan（SAP）described all the analysis plan to be performed and proposed in “A RANDOMIZED, CONTROLLED PHASE IB/II STUDY OF SURUFATINIB COMBINED WITH CAMRELIZUMAB AND THE AS REGIMEN AS FIRST-LINE TREATMENT FOR ADVANCED PANCREATIC CARCINOMA METASTATIC”, and is based on the protocol version 3.0 on March 7, 2022.

This analysis plan is drafted based on the relevant descriptions in the study protocol and specifies the efficacy and safety evaluation metrics. In accordance with the fundamental characteristics of each metric outlined in the protocol and the specific requirements of this study, it proposes detailed statistical analysis methods for the relevant evaluation criteria. Given that the final data distribution in clinical trials may present unforeseen variations (e.g., non-normality, small theoretical frequencies, etc.), the statistical analysis methods may undergo minor adjustments, and the presentation format of the corresponding analysis results may also be modified to some extent.

1. **Study objective and Basic information**
2. **Study objectives**

This phase Ib/II randomized study aimed to evaluate the efficacy and safety of surufatinib in combination with camrelizumab and nab-paclitaxel plus S-1 (NASCA) compared to nab-paclitaxel and gemcitabine as first-line treatment for locally advanced or metastatic pancreatic ductal adenocarcinoma (PDAC).

1. **Investigational medicinal products**

1.2.1. Dosing Regimen and Dosing Period

Test Group: surufatinib (200 mg/250 mg/300 mg orally, once daily continuous administration, with treatment cycles every 3 weeks) combined with camrelizumab (200 mg, intravenous infusion, q3w) and the AS three-week regimen (nab-paclitaxel: 120 mg/m2, I.V., on days 1 and 8; S-1: 40 mg BID orally, days 1-14, q3w), until intolerable toxicity or disease progression, death, or meeting other protocol-specified criteria for discontinuation of study treatment.

Control group: GnP 3-week regimen (nab-paclitaxel: 125 mg/m2, IV, d1, d8, q3w; gemcitabine: 1000 mg/m2, IV infusion over 30 minutes, d1, d8, q3w), until intolerable toxicity or disease progression, death, or reaching other criteria specified in the protocol for discontinuation of study treatment.

The above medications may be adjusted based on the adverse reactions of the subjects according to the protocol. Subjects will continue the treatment until disease progression, intolerable toxicity, withdrawal of informed consent, or discontinuation at the discretion of the investigator. The cycle date will be determined from the date of the first dose of the subject. In case of any dose interruption, omission, or underdose of the investigational product during the study, the treatment will continue according to the cycle specified in the protocol without supplementation or modification of cycle. However, it should be recorded in detail in the original data: if there is a drug omission, the time and the reason for the missed dose should be recorded in detail; if the drug is underdosed due to various reasons such as adverse drug reactions, it should be recorded in the subject's diary, original medical records, and eCRF.

1.2.2. Method of Administration

Surufatinib: it is recommended to take the investigational product with 200 mL of clear water within 1 hour after breakfast, striving to take the medication at the same time each day. If vomiting occurs after medication administration, it is not recommended to take the medication again unless the entire capsule is seen.

During the study, every effort should be made to ensure that the patient is dosed according to the protocol. If the patient misses the dose in the morning, it can be taken at any time before 10 p.m. on the same day. However, if the patient misses the prescribed medication and fails to take it on the same day, the patient must take the prescribed dose at the next time, but the missed dose will not need to be taken again. The investigator should record the actual dose and amount of drug taken by the patient, and record it in the original diagnosis and treatment record and CRF.

Intense exercise should be avoided during the trial; smoking, alcohol, and caffeinated beverages should be avoided. During the study participation, patients should avoid drinking high concentrations of grapefruit juice and consuming grapefruit, lime, and beverages containing these ingredients.

S-1 is administered orally. Generally, the initial dose for adults is determined according to body surface area as per the table below. Dosage is given orally twice daily after breakfast and dinner for 28 consecutive days with 14 days of rest as a treatment cycle. Doses are administered until the patient's condition worsened or the patient is no longer tolerable.

1. **Study Design**

This study is a prospective, single-center, randomized controlled Phase Ib/II study, enrolling patients with unresectable locally advanced or pancreatic carcinoma metastatic who have not received prior systemic therapy. In the Phase Ib stage, 9-15 subjects will be recruited using a 3+3 dose escalation scheme to explore the DLTs and RP2D of surufatinib combined with camrelizumab and the AS regimen. In the Phase II dose expansion stage, 90 subjects will be recruited and randomly assigned in a 1:1 ratio into the test group (45 cases) or the control group (45 cases), receiving surufatinib combined with camrelizumab and the AS regimen or standard first-line GN chemotherapy, until intolerable toxicity, disease progression, or death, to evaluate efficacy and safety.

This study is divided into three phases: screening phase, treatment phase, and follow-up phase. Every 6 weeks (±2 days), imaging methods will be used to assess the tumor status until disease progression (RECIST 1.1), death (during the patient’s treatment), or intolerable toxicity occurs, and record the tumor treatment and survival status after disease progression. Safety observations included: AEs, changes in laboratory values, vital signs, and changes in electrocardiograms, etc.

1. **Endpoint Indicators（Phase II）**
2. **Primary Study Endpoints**

• objective response rate (ORR)

1. **Secondary Study Endpoints**

• Progression-free survival (PFS)

• Disease control rate (DCR)

• Overall survival (OS)

• Duration of response (DOR)

• Time to response (TTR)

• Safety

1. **Sample Size Determination**

According to literature results, the rate in the test group is 0.49, and the rate in the control group is 0.23. With a superiority margin of 0, a one-sided alpha of 0.05, beta of 0.2, and a sample size ratio of 1 between the two groups (test group: control group), the calculated sample sizes are 40 cases in the test group and 40 cases in the control group, totaling 80 cases. Considering a 10% dropout rate, the total required sample size is 90 cases.

1. **Study population**

Intent-to-Treat Population (ITT Population): all randomized patients will be included for analysis according to the intention-to-treat principle. The ITT population is used for analyzing all efficacy endpoints.

Per-Protocol Analysis Population (PP Population): patients in the ITT Population who had no major protocol deviations affecting the efficacy assessment. The PP population is used for sensitivity analyses of OS and PFS.

Safety Population (Safety Set, SS Population): includes all randomized patients who received at least one dose of investigational product. The SS population is used for analyzing safety endpoints.

1. **Statistical Analysis**
2. **Statistical hypothesis and multiplicity considerations**
   1. **Statistical hypothesis**

This study is an exploratory study and will not involve formal hypothesis testing.

1. **Statistical analysis software**

All data summary/analysis will be performed using R 4.4.1 (or above).

1. **General statistical principles**
   - 1. **Descriptive statistics**

The descriptive statistical summaries will be based on the type of variable unless otherwise specified:

Quantitative variables will be summarized using descriptive statistics such as number of observations (n), mean, standard deviation, median, minimum, and maximum.

Qualitative or ordinal variables will be tabulated using frequency (N) and percent (%).

- - 1. **General Methods of Statistical Analysis**

All tests will be two-sided and the nominal level of statistical significance (α) will be 0.05 unless pre-defined, P-value ≤0.05 will be considered statistically significant for the tested hypothesis.

For parallel design and two treatment groups comparison:

- If the data are normally distributed and exhibit homogeneity of variance, independent t-test is used;
- If the data are normally distributed but does not exhibit homogeneity of variance, Satterthwaite t-test is used;
- For skewed data, independent Wilcoxon rank-sum test is used.

For qualitative data in parallel design and 2 group comparison:

- Chi-square test or Fisher's exact test will be used;
- For ordinal data, Wilcoxon rank-sum test will be used.

1. **Handling of Missing Data**
2. **Handling of missing values**

Descriptive summaries of endpoints, safety measures will be based on observed data only. No imputation of missing scores will be implemented.

1. **Statistical analysis content and methods**
2. **Subject Disposition**
   - The disposition of all subjects including the number of subjects with informed consents obtained and with screen failures will be summarized. All randomized subjects will be summarized by treatment and overall. The disposition summary will include the number and percentage of completers and non-completers, respectively, as well as the number and percentage of non-completers at by the primary discontinuation reason. In addition, the subject disposition for all enrolled subjects will be tabulated by study population.
   - Provide detailed listings of discontinued subjects.
   - Provide detailed listings of subjects excluded from any study population.
   - Incidence of protocol deviations/violations will be summarized by treatment and provide detailed listings of protocol deviations/violations.
   - Flowchart of subject distribution.
3. **Efficacy Analyses**

- **Primary Efficacy Analysis**

**Objective Response Rate (ORR)**

Objective response rate is defined as the proportion of patients with a best overall assessment of complete response or partial response. Clopper-Pearson method is used to calculate the 95% confidence intervals of the rates in each group and Newcombe Wilson is used to calculate 90% confidence intervals of the rates difference. The superiority of the experimental group versus the control group will be established if the lower bound of the two-sided 90% confidence interval (CI) for the difference between groups (experimental - control) exceeds 0.

Depending on the characteristics of the data, rates between groups are compared using chi-square test or Fisher exact probability method.

The OR is calculated by univariate logistic regression analysis. Using treatment group allocation as the independent variable and ORR as the dependent variable, a logistic regression model will be constructed to estimate the treatment effect.

- **Secondary Efficacy Analysis**

**Progression-free Survival (PFS)**

PFS is defined as the time (in days) from randomization to disease progression or death. Overall survival is compared between the test group and the control group using a log-rank test. The Kaplan-Meier method will be used to estimate the median survival time of both treatment groups, and Kaplan-Meier curves will be plotted to provide a visually intuitive description of the differences between treatment groups. The Cox proportional hazards model will be used to estimate the hazard ratio (HR) and corresponding 95% CIs.

**Overall Survival (OS)**

Overall survival (OS) is defined as the time from randomization to death from any cause. For patients who have not reported death at the time of analysis, the date of their last known survival will be taken as the censoring date. The analysis method is the same as for PFS.

**Disease Control Rate (DCR)**

Disease control rate is defined as the proportion of patients with a best overall assessment of complete response, partial response, or stable disease. For patients assessed as having stable disease, at least one lesion assessment meeting the SD criteria is required at least 6 weeks after starting medication. The analysis method is the same as that for ORR.

**Duration of response (DOR)**

Duration of response was defined as the time from the first documented CR or PR to disease progression or death. The Kaplan-Meier method will be used to estimate the median survival time of both treatment groups.

**Time to response (TTR)**

Time to response was defined as the time from randomization to the first occurrence of CR or PR. The Kaplan-Meier method will be used to estimate the median survival time of both treatment groups.

The comparison of ORR, PFS, OS, DCR, DOR and TTR between treatment arms will be based on ITT.

1. **Safety Analysis**

Safety will be evaluated by summaries of AEs, changes in laboratory results, and changes in vital signs.

All AEs will be graded according to NCI CTCAE (version 5.0). AEs will be coded using the Medical Dictionary for Regulatory Activities (MedDRA). The number and incidence of AEs will be summarized by human system organ class (SOC) with corresponding terms. All SAEs (including deaths) and AEs leading to dose reduction, dose interruption, and discontinuation of investigational product should be tabulated and summarized separately.

All laboratory assessments will be summarized and tabulated. Changes in laboratory test results will be graded according to NCI CTCAE (version 5.0), and comparison of baseline and post-treatment maximum toxicity will be presented in shift tables. Patients with laboratory abnormalities of CTCAE grade 3 or 4 will be tabulated separately.

Changes in physical examinations, vital signs, and ECOG Performance Status scores will be compared with baseline and will be analyzed descriptively.

Patients exhibiting abnormalities in electrocardiogram and echocardiogram will be tabulated. Shift tables will be used to summarize changes from baseline to abnormality for each treatment group.

1. **Discussion of Statistical Results**

When drafting the statistical analysis report, provide a concise description based on the statistical results from the "Statistical Analysis Tables/Figures/Listings".

1. **Amendment of statistical content from the protocol**

None.

1. **Appendix**

**Appendix** Statistical Analysis Tables/Figures/Listings

1. **Reference**
2. National Medical Products Administration. Guidelines for Biostatistics in Drug Clinical Trials. 2016.
3. National Medical Products Administration. Guidelines for Data Management and Statistical Analysis Plans in Drug Clinical Trials. 2021.
4. Feng Chen, Jielai Xia. Clinical Trial Statistics [M]. Beijing: People's Medical Publishing House. 2018.
5. ICH. E3: Structure and Content of Clinical Study Reports. 1995.
6. ICH. E9: Statistical Principles for Clinical Trials. 1998.
